# Supplementary material for: Seasonality, molecular epidemiology, and virulence of Respiratory Syncytial Virus (RSV): A perspective into the Brazilian Influenza Surveillance Program
Source: PLoS One. 2021 May 18;16(5):e0251361. doi: 10.1371/journal.pone.0251361 (PMC8130917; doi:10.1371/journal.pone.0251361)
Supplement: S1 Table — “F”, “R”, and “P”, represent the sequence of the forward and reverse primers, and the probe, respectively. A synthetic DNA fragment from RSV was included in a pMA-t vector. (DOCX) [file pone.0251361.s006.docx]

**S1 Table.**

| **Target** | **Sequence (3’-5’)** | **Reference** |
| --- | --- | --- |
| **Influenza A^1^** | F: GAC CRA TCC TGT CAC CTC TGA C | CDC, 2009 |
|  | R: AGG GCA TTY YGG ACA AAK CGT CTA |  |
|  | P: TGC AGT CCT CGC TCA CTG GGC ACG |  |
| **Influenza B^1^** | F: TCC TCA AYT CAC TCT TCG AGC G | CDC, 2009 |
|  | R: CGG TGC TCT TGA CCA AAT TGG |  |
|  | P: CCA ATT CGA GCA GCT GAA ACT GCG GTG |  |
|  | F: AGATTTGGACCTGCGAGCG |  |
| **RNase P^1^** | R: GAGCGGCTGTCTCCACAAGT | CDC, 2009 |
|  | P: TTCTGACCTGAAGGCTCTGCGCG |  |
| **RSV screening^1^** | F: GGC AAA TAT GGA AAC ATA CGT GAA | Fry *et al*., 2010 |
|  | R: TCT TTT TCT AGG ACA TTG RAY TGA ACA G |  |
|  | P: CTG TGT ATG TGG AGC CTT CGT GAA GCT |  |
|  | F: GAGCCATCTATTGCTTACATTTGCTTCTGA | Huang *et al*., 1989 |
| **β-globin^1^** | R: CCTGCCCAGGGCCTCACCACCAACTTCATC |  |
|  | P: CACGTTCACCTTGCCCCACAGG |  |
| **RSV-A^2^** | F: GCTCTTAGCAAAGTCAAGTTGAATGA | Hu *et al*. 2003 |
|  | R: AACATGCCACATAACTTATTGAT | de Paris *et al*., 2012 |
|  | P: ACACTCAACAAAGATCAACTTCTGTCATCCAGC |  |
| **RSV-B^2^** | F: GATGGCTCTTAGCAAAGTCAAGTTAA |  |
|  | R: TGTCAATATTATCTCCTGTACTACGTTGAA | Hu *et al*. 2003 |
|  | P: TGATACATTAAATAAGGATCAGCTGCTGTCATCCA |  |
| **RSV sequencing^3^** | F: AGAGACCCAAAAACACYAGCCAA | Zlateva, *et al*., 2005 |
|  | R: ACAGGGAACGAAGTTGAACACTTCA |  |
| **RSV fragment sequence** | GAGCCATCTATTGCTTACATTTGCTTCTGACACAACTGTGTTCACTAGCAACCTCAAACAGACACCATGGTGCACCTGACTCCTGAGGAGAAGTCTGCCGTTACTGCCCTGTGGGGCAAGGTGAACGTGGATGAAGTTGGTGGTGAGGCCCTGGGCAGG | - |
| **β-globin synthetic fragment** | GAGCCATCTATTGCTTACATTTGCTTCTGACACAACTGTGTTCACTAGCAACCTCAAACAGACACCATGGTGCACCTGACTCCTGAGGAGAAGTCTGCCGTTACTGCCCTGTGGGGCAAGGTGAACGTGGATGAAGTTGGTGGTGAGGCCCTGGGCAGG | - |

**PCR conditions:**

**^1^** 50°C/30 minutes, 95°C/2 minutes, 45 cycles of 95°C/15 seconds, and 55°C/30 seconds.

**^2^** 45°C/25 minutes, 95°C/2 minutes, 45 cycles of 95°C/15 seconds, and 55°C/30 seconds.

**^3^** 55°C/30 minutes, 40 cycles of 94°C/30 seconds, 60°C/1 minute, 72°C/1 minute, and a final extension at 72°C/10 minutes.

**References:**

Centers for Disease Control and Prevention Human Influenza Virus Real-time RT-PCR Detection, 2009

Fry AM, Chittaganpitch M, Baggett HC, Peret TCT, Dare RK, Sawatwong P, et al. The Burden of Hospitalized Lower Respiratory Tract Infection due to Respiratory Syncytial Virus in Rural Thailand. PLoS ONE. 2010;5: e15098. doi:10.1371/journal.pone.0015098

Huang S, Wong C, Antonarakis SE, Ro-lien T, Lo WHY, Kazazian HH. The same “TATA” box I -thalassemia mutation in Chinese and US blacks: another example of independent origins of mutation. Hum Genet. 1986;74: 162–164. doi:10.1007/BF00282081

Hu A, Colella M, Tam JS, Rappaport R, Cheng S-M. Simultaneous Detection, Subgrouping, and Quantitation of Respiratory Syncytial Virus A and B by Real-Time PCR. J Clin Microbiol. 2003;41: 149–154. doi:10.1128/JCM.41.1.149-154.2003

de-Paris F, Beck C, Machado ABMP, Paiva RM, da Silva Menezes D, de Souza Nunes L, et al. Optimization of one-step duplex real-time RT-PCR for detection of influenza and respiratory syncytial virus in nasopharyngeal aspirates. J Virol Methods. 2012;186: 189–192. doi:10.1016/j.jviromet.2012.07.008

Zlateva KT, Lemey P, Moes E, Vandamme A-M, Van Ranst M. Genetic Variability and Molecular Evolution of the Human Respiratory Syncytial Virus Subgroup B Attachment G Protein. J Virol. 2005;79: 9157–9167. doi:10.1128/JVI.79.14.9157-9167.2005
