# Supplementary material for: Seasonality, molecular epidemiology, and virulence of Respiratory Syncytial Virus (RSV): A perspective into the Brazilian Influenza Surveillance Program
Source: PLoS One. 2021 May 18;16(5):e0251361. doi: 10.1371/journal.pone.0251361 (PMC8130917; doi:10.1371/journal.pone.0251361)
Supplement: S2 Table — (DOCX) [file pone.0251361.s007.docx]

**S2 Table.**

| Subtype | Accession n° | Genotype | Origin |
| --- | --- | --- | --- |
| RSV-A | M74568 | A2 | ND |
|  | AF233902 | GA1 | USA |
|  | AF233914 | GA1 | USA |
|  | AF233917 | GA1 | USA |
|  | AF233923 | GA2 | USA |
|  | AF233900 | GA2 | USA |
|  | AF233915 | GA2 | USA |
|  | KJ130646 | ON1 | CHN |
|  | JN257693 | ON1 | CAN |
|  | MG062683 | ON1 | MEX |
|  | AF233911 | GA3 | CAN |
|  | AF233920 | GA3 | USA |
|  | AF233921 | GA3 | USA |
|  | AF065254 | GA4 | USA |
|  | AF233912 | GA5 | USA |
|  | AF233916 | GA5 | USA |
|  | AF233919 | GA5 | USA |
|  | AF233908 | GA5 | CAN |
|  | AF233909 | GA5 | USA |
|  | AF233901 | GA6 | USA |
|  | AF233918 | GA6 | USA |
|  | AF233904 | GA7 | CAN |
|  | AF233907 | GA7 | CAN |
|  | AF233910 | GA7 | USA |
|  | KC297351 | NA1 | CHN |
|  | KJ130616 | NA1 | CHN |
|  | AB470479 | NA2 | JPN |
|  | KC297260 | NA3 | CHN |
|  | KC297374 | NA4 | CHN |
|  | AF348808 | SAA1 | ZAF |
|  | AF348807 | SAA1 | ZAF |
|  | AF065255 | GA5 | USA |
|  | AF065256 | GA2 | USA |
|  | AF065257 | GA1 | USA |
|  | AF065258 | GA2 | USA |
| RSV-B | M17213 | GB1 | ND |
|  | AF065250 | GB1 | USA |
|  | AF013254 | GB1 | ND |
|  | M73540 | GB1 | USA |
|  | AF065251 | GB2 | USA |
|  | AF348811 | GB4 | ZAF |
|  | DQ171853 | GB4 | NZL |
|  | AY672698 | GB4 | ARG |
|  | AF233931 | GB4 | USA |
|  | AF348825 | SAB1 | ZAF |
|  | AY660682 | SAB1 | KEN |
|  | KC263042 | SAB1 | KEN |
|  | AF348821 | SAB2 | ZAF |
|  | AY488799 | SAB2 | URU |
|  | JX489439 | SAB4 | BRA |
|  | JX489422 | SAB4 | BRA |
|  | JN120007 | SAB4 | KHM |
|  | JN119987 | SAB4 | KHM |
|  | AY488804 | URU1 | URU |
|  | AY488805 | URU1 | URU |
|  | AY488794 | URU1 | URU |
|  | AB161413 | URU1 | JPN |
|  | AJ290205 | URU1 | GBR |
|  | AY333361 | URU2 | ARG |
|  | AY488802 | URU2 | URU |
|  | AY488806 | URU2 | URU |
|  | KC297462 | CB1 | CHN |
|  | KC297450 | CB1 | CHN |
|  | AY333364 | BA1 | ARG |
|  | DQ227380 | BA1 | ARG |
|  | GQ150687 | BA1 | ESP |
|  | HM459857 | BA2 | JPN |
|  | HM459856 | BA2 | JPN |
|  | AY751122 | BA2 | BEL |
|  | DQ227370 | BA2 | ARG |
|  | DQ227392 | BA3 | ARG |
|  | AB175820 | BA5 | JPN |
|  | AB603480 | BA5 | JPN |
|  | AY751105 | BA6 | BEL |
|  | AY751110 | BA6 | BEL |
|  | HM459864 | BA7 | JPN |
|  | KC297492 | BA7 | CHN |
|  | AB603476 | BA7 | JPN |
|  | HM459873 | BA8 | JPN |
|  | HM459872 | BA8 | JPN |
|  | HM459871 | BA8 | JPN |
|  | KC342347 | BA9 | THA |
|  | KY649190 | BA9 | IND |
|  | HM459891 | BA10 | JPN |
|  | HM459884 | BA10 | JPN |
|  | KC297426 | BA10 | CHN |
|  | KF301020 | BA10 | PAN |

^1^ND: not determined.
