# Supplementary material for: Seasonality, molecular epidemiology, and virulence of Respiratory Syncytial Virus (RSV): A perspective into the Brazilian Influenza Surveillance Program
Source: PLoS One. 2021 May 18;16(5):e0251361. doi: 10.1371/journal.pone.0251361 (PMC8130917; doi:10.1371/journal.pone.0251361)
Supplement: S3 Table — The collection date of some sequences was unavailable. (DOCX) [file pone.0251361.s008.docx]

**S3 Table.**

| Subtype | Accession n° | Genotype | Collection date | Origin |
| --- | --- | --- | --- | --- |
| RSV-A | KP792358 | NA1 | 2007 | ESP |
|  | Z33494 | GA5 | 1990 | URU |
|  | Z33455 | GA7 | 1992 | ESP |
|  | Z33427 | GA1 | 1990 | URU |
|  | Z33426 | GA3 | 1990 | URU |
|  | Z33422 | GA2 | 1989 | URU |
|  | Z33417 | GA7 | 1992 | ESP |
|  | Z33416 | GA3 | 1990 | ESP |
|  | Z33414 | GA3 | 1993 | ESP |
|  | M11486 | GA1 | 1985 | USA |
|  | KP258696 | SAA | 1986 | USA |
|  | KF300972 | NA1 | 2010 | PAN |
|  | KP792359 | NA1 | 2008 | ESP |
|  | KC297381 | NA4 | 2007 | CHN |
|  | KC297324 | NA4 | 2011 | CHN |
|  | KC297277 | NA3 | 2011 | CHN |
|  | KC297292 | NA3 | 2011 | CHN |
|  | AY114150 | GA5 | 2003 | SGP |
|  | DQ985132 | SAA | 1997 | BEL |
|  | JQ901453 | NA2 | 2002 | NLD |
|  | AF065254 | GA4 | 1998 | ND^1^ |
|  | AF065407 | GA1 | 1998 | USA |
|  | AF065255 | GA5 | 1998 | ND^1^ |
|  | AY114149 | GA2 | 2002 | SGP |
|  | DQ985132 | GA6 | 1986 | USA |
|  | AF065256 | GA2 | 1998 | USA |
|  | KC297260 | NA3 | 2011 | CHN |
|  | DQ985131 | GA6 | 1988 | BEL |
|  | KM042392 | ON1 | 2013 Feb | USA |
|  | MH760648 | ON1 | 2015 Jul | AUS |
|  | MH760647 | ON1 | 2015 Jun | AUS |
|  | MH760646 | ON1 | 2014 Ago | AUS |
|  | MH760643 | ON1 | 2014 Sep | AUS |
|  | MH760640 | ON1 | 2014 Aug | AUS |
|  | MH760632 | ON1 | 2016 Aug | AUS |
|  | MH760629 | ON1 | 2016 Aug | AUS |
|  | MH760628 | ON1 | 2016 Aug | AUS |
|  | MH760625 | ON1 | 2016 Aug | AUS |
|  | MH760624 | ON1 | 2016 Aug | AUS |
|  | MH760623 | ON1 | 2016 Aug | AUS |
|  | MH760617 | ON1 | 2016 Oct | AUS |
|  | MH760611 | ON1 | 2016 Nov | AUS |
|  | MH760610 | ON1 | 2016 Nov | AUS |
|  | MH760607 | ON1 | 2013 Jul | AUS |
|  | MH760602 | ON1 | 2012 Aug | AUS |
|  | MH760590 | ON1 | 2016 Jul | AUS |
|  | MH182025 | ON1 | 2014 Dec | KEN |
|  | MH181993 | ON1 | 2013 Oct | KEN |
|  | MH181990 | ON1 | 2013 Mar | KEN |
|  | MH181984 | ON1 | 2013 Jan | KEN |
|  | MH181975 | ON1 | 2012 Dec | KEN |
|  | MH181935 | ON1 | 2012 Oct | KEN |
|  | MH181924 | ON1 | 2012 Jun | KEN |
|  | MH181923 | ON1 | 2012 Jun | KEN |
|  | MH181908 | ON1 | 2012 | KEN |
|  | MG793382 | ON1 | 2015 | LBN |
|  | MG062688 | ON1 | 2011 | MEX |
|  | MG062687 | ON1 | 2013 | MEX |
|  | MG062685 | ON1 | 2011 | MEX |
|  | MG062683 | ON1 | 2009 | MEX |
|  | MF614947 | ON1 | 2013 | CHN |
|  | KY865205 | ON1 | 2011 | MEX |
|  | KY654512 | ON1 | 2012 | PHL |
|  | KX894805 | ON1 | 2013 | USA |
|  | KX894803 | ON1 | 2013 | USA |
|  | KX765970 | ON1 | 2014 | NZL |
|  | KX765939 | ON1 | 2013 | NZL |
|  | KX765932 | ON1 | 2015 | NZL |
|  | KX765926 | ON1 | 2012 | NZL |
|  | KX765925 | ON1 | 2015 | NZL |
|  | KX765915 | ON1 | 2014 | NZL |
|  | KX655644 | ON1 | 2012 | JOR |
|  | KX655626 | ON1 | 2013 | JOR |
|  | KU950677 | ON1 | 2012 | USA |
|  | KU950673 | ON1 | 2012 | USA |
|  | KU950628 | ON1 | 2013 | USA |
|  | KU950627 | ON1 | 2013 | USA |
|  | KU950615 | ON1 | 2012 | USA |
|  | KU950610 | ON1 | 2013 | USA |
|  | KU950592 | ON1 | 2012 | USA |
|  | KU950590 | ON1 | 2013 | USA |
|  | KU950583 | ON1 | 2013 | USA |
|  | KU950567 | ON1 | 2012 | USA |
|  | KU950550 | ON1 | 2012 | USA |
|  | KU950544 | ON1 | 2013 | USA |
|  | KU950531 | ON1 | 2013 | USA |
|  | KU950523 | ON1 | 2014 | USA |
|  | KU950521 | ON1 | 2012 | USA |
|  | KU950520 | ON1 | 2013 | USA |
|  | KU950513 | ON1 | 2012 | USA |
|  | KU950502 | ON1 | 2012 | USA |
|  | KU950493 | ON1 | 2012 | USA |
|  | KU950492 | ON1 | 2012 | USA |
|  | KU950486 | ON1 | 2012 | USA |
|  | KU950472 | ON1 | 2013 | USA |
|  | KU839637 | ON1 | 2014 | USA |
|  | KM042390 | ON1 | 2013 | USA |
|  | KJ672475 | ON1 | 2013 Jan | USA |
|  | KJ672471 | ON1 | 2013_Mar | USA |
|  | KJ672469 | ON1 | 2013 Feb | USA |
|  | KJ672465 | ON1 | 2012 Dec | USA |
|  | KJ672441 | ON1 | 2013 Mar | USA |
|  | KJ672433 | ON1 | 2013 Feb | USA |
|  | KJ672432 | ON1 | 2013 Mar | USA |
|  | KJ672429 | ON1 | 2013 Feb | USA |
|  | KJ672428 | ON1 | 2013 Jan | USA |
|  | KJ627264 | ON1 | 2012 May | PER |
|  | KC559440 | ON1 | 2012 Feb | CHN |
|  | KC342413 | ON1 | 2011 Aug | THA |
|  | JX627336 | ON1 | 2011 Dec | KOR |
|  | JN257693 | ON1 | 2010 Dec | CAN |
| RSV-B | AF065250 | GB1 | 1990-91 | USA |
|  | M73542 | GB1 | 1985 | USA |
|  | AF065251 | GB2 | 1992-93 | USA |
|  | KC297470 | GB2 | 2011 | CHN |
|  | AF065252 | GB3 | 1992-93 | USA |
|  | AF065253 | GB3 | 1992-93 | USA |
|  | AY333361 | GB4 | 1990 | MON |
|  | M73543 | GB4 | 1989 | ND^1^ |
|  | AY660682 | SAB1 | 2003 | KEN |
|  | JF704213 | SAB1 | 1998 | ZAF |
|  | AY327815 | SAB2 | 2000 | QAT |
|  | DQ171866 | SAB3 | 2001 | NZL |
|  | JF704216 | SAB3 | 1998 | ZAF |
|  | DQ270231 | SAB4 | ND^1^ | CHN |
|  | KC297430 | SAB4 | 2009 | CHN |
|  | AY333362 | BA | 1999 | ARG |
|  | AY333364 | BA | 1995-2001 | ARG |
|  | AY751108 | BA | 2001 | BEL |
|  | AY751110 | BA | 2003 | BEL |
|  | AY751117 | BA | 2002 | BEL |
|  | DQ227373 | BA | 2002 | ARG |
|  | DQ227381 | BA | 2002 | ARG |
|  | DQ227396 | BA | 2004 | ARG |
|  | DQ227389 | BA | 2003 | ARG |
|  | DQ227393 | BA | 2003 | ARG |
|  | KC297435 | BA | 2009 | CHN |
|  | KC297477 | BA | 2012 | CHN |
|  | KC297490 | BA | 2009 | CHN |
|  | KC297456 | BA | 2010 | CHN |
|  | DQ227395 | BA | 2004 | ARG |
|  | DQ985142 | BA | 2003 | BEL |
|  | KF300952 | BA | 2008 | PAN |
|  | KX371868 | BA | 2008 | PAN |
|  | KC297492 | BA | 2009 | CHN |
|  | KC297476 | BA | 1905 | CHN |
|  | KC710985 | BA | 2012 | NLD |
|  | KT781377 | BA | 2014 | CHN |
|  | KX765912 | BA | 2014 | NZL |
|  | KM586838 | BA | 2013 | CHN |
|  | KU950682 | BA | 2012 | USA |
|  | KX765959 | BA | 2013 | NZL |
|  | KM586835 | BA | 2012 | CHN |
|  | LC311394 | BA | 2013 | PHI |
|  | MH760724 | BA | 2013 | AUS |
|  | KM586840 | BA | 2013 | CHN |
|  | MH760666 | BA | 2013 | AUS |
|  | KT781376 | BA | 2014 | CHN |
|  | MF443156 | BA | 2014 | ESP |
|  | KC710996 | BA | 2012 | NLD |
|  | KX655648 | BA | 2013 | JOR |
|  | KT781363 | BA | 2014 | CHN |
|  | LC311396 | BA | 2013 | PHI |
|  | KU839625 | BA | 2014 | USA |
|  | KU950607 | BA | 2013 | USA |
|  | KY828376 | BA | 2015 | BRA |
|  | LC311365 | BA | 2015 | PHI |
|  | KX775817 | BA | 2015 | KEN |
|  | MH742858 | BA | 2016 | KEN |
|  | MH742865 | BA | 2016 | KEN |
|  | KT781370 | BA | 2014 | CHN |
|  | MH742866 | BA | 2016 | KEN |
|  | MH742869 | BA | 2017 | KEN |
|  | KM517573 | BA | 2013 | CHN |
|  | LC384997 | BA | 2014 | PHI |
|  | MH760725 | BA | 2014 | AUS |
|  | MH760729 | BA | 2015 | AUS |
|  | LC384999 | BA | 2014 | PHI |
|  | MH760672 | BA | 2016 | AUS |
|  | KY249660 | BA | 2013 | GBR |
|  | KY674984 | BA | 1905 | USA |
|  | LC311369 | BA | 2015 | PHI |
|  | MH760695 | BA | 2016 | AUS |
|  | MH760721 | BA | 2016 | AUS |
|  | MH760702 | BA | 2016 | AUS |
|  | MG839547 | BA | 2016 | ARG |
|  | KX765906 | BA | 2015 | NZL |
|  | MG773268 | BA | 2016 | ARG |
|  | MH760679 | BA | 2016 | AUS |
|  | MH742890 | BA | 2017 | KEN |
|  | MH742820 | BA | 2017 | KEN |
|  | MH760731 | BA | 2015 | AUS |
|  | KX775816 | BA | 2016 | KEN |
|  | MH742838 | BA | 2017 | KEN |
|  | MH760668 | BA | 2016 | AUS |
|  | MH760728 | BA | 2015 | AUS |
|  | MH760694 | BA | 2016 | AUS |
|  | KX775814 | BA | 2015 | KEN |
|  | MH742825 | BA | 2016 | KEN |
|  | MH742840 | BA | 2017 | KEN |
|  | MH742826 | BA | 2017 | KEN |
|  | MH760720 | BA | 2016 | AUS |
|  | KY249682 | BA | 2016 | GBR |
|  | KX765935 | BA | 2015 | NZL |
|  | KT781362 | BA | 2014 | CHN |
|  | KT781373 | BA | 2014 | CHN |
|  | MH760685 | BA | 2016 | AUS |
|  | KT781404 | BA | 2014 | CHN |
|  | MH760683 | BA | 2016 | AUS |
|  | MH760708 | BA | 2016 | AUS |
|  | BRA.ES/5 | BA | 7 | BRA |
|  | MG773266 | BA | 2016 | ARG |
|  | MH760687 | BA | 2016 | AUS |
|  | LC324678 | BA | 1905 | JPN |
|  | LC385006 | BA | 2016 | PHI |
|  | MH760671 | BA | 2016 | AUS |
|  | MH760722 | BA | 2016 | AUS |
|  | MH760673 | BA | 2016 | AUS |
|  | MH760680 | BA | 2016 | AUS |
|  | MH760714 | BA | 2016 | AUS |
|  | MH760675 | BA | 2016 | AUS |
|  | MH760710 | BA | 2016 | AUS |
|  | MH760723 | BA | 2016 | AUS |
|  | MH742920 | BA | 2016 | KEN |
|  | KT781360 | BA | 2014 | CHN |
|  | BRA.ES/5 | BA | 16 | 0/2 |
|  | KY249683 | BA | 2016 | GBR |
|  | MH760703 | BA | 2016 | AUS |
|  | MH742896 | BA | 2017 | KEN |
|  | MH742907 | BA | 2017 | KEN |
|  | MH760701 | BA | 2016 | AUS |
|  | MH760670 | BA | 2016 | AUS |
|  | MH760689 | BA | 2016 | AUS |
|  | JF714708 | BA | 2009 | SAU |
|  | KF246586 | BA | 2009 | IND |
|  | HQ699300 | BA | 2009 | KOR |
|  | HQ699304 | BA | 2009 | KOR |
|  | KF300951 | BA | 2011 | PAN |

^1^ND: not determined.
