# Supplementary material for: Seasonality, molecular epidemiology, and virulence of Respiratory Syncytial Virus (RSV): A perspective into the Brazilian Influenza Surveillance Program
Source: PLoS One. 2021 May 18;16(5):e0251361. doi: 10.1371/journal.pone.0251361 (PMC8130917; doi:10.1371/journal.pone.0251361)
Supplement: S5 Table — (DOCX) [file pone.0251361.s010.docx]

**S5 Table.**

| **Average** | | | | **Median** | | | |
| --- | --- | --- | --- | --- | --- | --- | --- |
| **Estimate** | **Standard error** | **Interval** | | **Estimate** | **Standard error** | **Interval** | |
|  |  | **LI** | **LS** |  |  | **LI** | **LS** |
| 8.18 | 0.45 | 7.30 | 9.05 | 7.00 | 0.56 | 5.91 | 8.09 |
