# Supplementary material for: Seasonality, molecular epidemiology, and virulence of Respiratory Syncytial Virus (RSV): A perspective into the Brazilian Influenza Surveillance Program
Source: PLoS One. 2021 May 18;16(5):e0251361. doi: 10.1371/journal.pone.0251361 (PMC8130917; doi:10.1371/journal.pone.0251361)
Supplement: S6 Table — (DOCX) [file pone.0251361.s011.docx]

**S6 Table.**

| **Parameter** | **ρ** | **Q** | **p-value** |
| --- | --- | --- | --- |
| Viral load | -0,009 | 0,003 | 0,95 |
