# Supplementary material for: Seasonality, molecular epidemiology, and virulence of Respiratory Syncytial Virus (RSV): A perspective into the Brazilian Influenza Surveillance Program
Source: PLoS One. 2021 May 18;16(5):e0251361. doi: 10.1371/journal.pone.0251361 (PMC8130917; doi:10.1371/journal.pone.0251361)
Supplement: S7 Table — (DOCX) [file pone.0251361.s012.docx]

**S7 Table.**

| Year | Onset | | | Peak | | | End | | | RSV+ | Duration | | Average precipitation  (mm) | Average temperature (°C) | Average humidity  (%) |
| --- | --- | --- | --- | --- | --- | --- | --- | --- | --- | --- | --- | --- | --- | --- | --- |
|  | **EW** | **Month** | **Season** | **EW** | **Month** | **Season** | **EW** | **Month** | **Season** |  | **Weeks** | **Months** |  |  |  |
| 2016 | 12 | March | Fall | 19 | May | Fall | 32 | August | Winter | 150 | 20 | 5 | 7.78 | 22.23 | 77.83 |
| 2017 | 12 | March | Fall | 16 20 | April May | Fall | 31 | July  August | Winter | 72 | 19 | 4.75 | 15.30 | 21.32 | 78.72 |
| 2018 | 3 | January | Summer | 14 | April | Fall | 27 | July | Fall | 115 | 24 | 6 | 29.24 | 23.13 | 78.36 |
