# Supplementary material for: Seasonality, molecular epidemiology, and virulence of Respiratory Syncytial Virus (RSV): A perspective into the Brazilian Influenza Surveillance Program
Source: PLoS One. 2021 May 18;16(5):e0251361. doi: 10.1371/journal.pone.0251361 (PMC8130917; doi:10.1371/journal.pone.0251361)
Supplement: Attachment — Submitted filename: PONE-D-20-30854_R1.pdf [file pone.0251361.s017.pdf]

PLOS ONE

Seasonality, molecular epidemiology and virulence of Respiratory Syncytial Virus (RSV): a perspective into the Brazilian Influenza Surveillance Program

--Manuscript Draft--

**Manuscript Number:**	PONE-D-20-30854R1
**Article Type:**	Research Article
**Full Title:**	Seasonality, molecular epidemiology and virulence of Respiratory Syncytial Virus (RSV): a perspective into the Brazilian Influenza Surveillance Program
**Short Title:**	Landscape of Respiratory Syncytial Virus (RSV)
**Corresponding Author:**	Lucas Alves Vianna, M.D<br>UFES: Universidade Federal do Espirito Santo<br>Vitoria, ES BRAZIL
**Keywords:**	RSV; Viral load; Genotypes; Severity; seasonality; phylogenetics.
**Abstract:**	Background: Respiratory Syncytial Virus (RSV) is the main cause of pediatric morbidity and mortality. The complex evolution of RSV creates a need for worldwide surveillance, which may assist in the understanding of multiple viral aspects.<br><br>Objectives: This study aimed to investigate RSV features under the Brazilian Influenza Surveillance Program, evaluating the role of viral load and genetic diversity in disease severity and the influence of climatic factors in viral seasonality.<br><br>Methodology: We have investigated the prevalence of RSV in children up to 3 years old with severe acute respiratory infection (SARI) in the Espirito Santo State (ES), Brazil, from 2016 to 2018. RT-qPCR allowed for viral detection and viral load quantification, to evaluate association with clinical features and mapping of local viral seasonality. Gene G sequencing and phylogenetic reconstruction demonstrated local genetic diversity.<br><br>Results: Of 632 evaluated cases, 56% were caused by RSV, with both subtypes A and B co-circulating throughout the years. A discrete inverse association between average temperature and viral circulation was observed. No correlation between viral load and severity was observed, but children infected with RSV-A presented higher clinical severity score (CSS) median, stayed longer in the hospital, required more intensive care and ventilatory support than those infected by RSV-B. Regarding RSV diversity, some local genetic groups were observed in the main genotypes circulation RSV-A ON1 and RSV-B BA, with strains showing modifications in the G gene amino acid chain.<br><br>Conclusion: Local RSV studies using the Brazilian Influenza Surveillance Program are relevant because they can reveal useful information, contributing to the global RSV surveillance. Understanding seasonality, virulence and genetic diversity can support the suitability of future antiviral drugs and vaccines and assist in the administration of prophylactic strategies.
**Order of Authors:**	Lucas Alves Vianna, M.D
	Marilda Mendonça Siqueira
	Lays Paula Bondi Volpini
	Iuri Drumond Louro
	Paola Cristina Resende
**Opposed Reviewers:**	
**Response to Reviewers:**	Editor comments:<br>Comment 1: Please ensure that your manuscript meets PLOS ONE's style requirements, including those for file naming. The PLOS ONE style templates can be found at:

Powered by Editorial Manager® and ProduXion Manager® from Aries Systems Corporation

https://journals.plos.org/plosone/s/file?id=wjVg/PLOSOne_formatting_sample_main_body.pdf and

https://journals.plos.org/plosone/s/file?id=ba62/PLOSOne_formatting_sample_title_authors_affiliations.pdf

Answer: After a careful review, we have modified the formatting of the headings and legends of the supplementary figures and tables to meet PLOS ONE's style requirements. We also corrected some tables that presented values highlighted in red, contrary to the rules of PLOS ONE. Finally, we increased the font size of the Materials and methods, Results and Discussion subheadings to 16 pt, according to rules.

Comment 2: We note that you included minors (age<18) in your study. Please provide additional details regarding minor's consent. In the ethics statement in the Methods and online submission information, please ensure that you have specified whether you obtained consent from parents or guardians. If the need for consent was waived by the ethics committee, please include this information."
Answer: We agree with this observation. The sentence "The need for parents or guardians' consent was waived by the ethics committee." was included in "Ethics Statement" section. Please, check the lines 207-208.

Reviewer #1 comments:
1.Title - Revise the title to reflect the key findings of the research.
Answer: To address this comment, we have changed the title to: "Seasonality, molecular epidemiology and virulence of Respiratory Syncytial Virus (RSV): a perspective into the Brazilian Influenza Surveillance Program". However, considering that this study addressed several aspects of RSV, the inclusion of key findings would make the title too large and we have opted to make it shorter and easier to read.

2.Introduction - Revise the introduction and make it shorter and its current form it distracts the reader.

Answer: We have changed the introduction and eliminated sentences that, although interesting, would not affect the understanding of the objectives. However, Introduction size reduction was small. It turns out that this study has addressed multiple aspects of RSV (e.g.: prevalence, association between severity and subtypes, viral load, seasonality and association with climatic factors and phylogenetic aspects), as we understand that these are important aspects to discuss.

3.Methodology - Organize the Methodology - Study population, experimental methods - RT-qPCR, sequencing etc. in a brief form that gives a good understanding of the sequence of events with relevant methodology.

Answer: We have changed the way methods are presented, in order to maintain the same pattern presented at the results.

Thus, methods are now as follows:

1. Population sampling, study period and location
2. RSV and Influenza detection and subtyping
3. Clinical and epidemiological data collection
4. Viral load quantification
5. Climate data collection
6. Partial amplification and sequencing of glycoprotein gene
7. RSV genotyping and gene G phylogenetic reconstruction
8. Statistical treatment.
9. Data availability
10. Ethics Statement

4.Results - They need to be organized in the order of appearance as in the Methodology. You do not have to replicate all the information given in the Tables in the texts. Redundancy also distracts the reader and I found it difficult to organize the results to understand the authors' way of cohesion.

Powered by Editorial Manager® and ProduXion Manager® from Aries Systems Corporation

Answer: We think this comment will make the manuscript easier to read. As recommended, we have reorganized the objectives in the same pattern presented in the results, and eliminated redundant information.

5.Discussion - Again follow the order of your results in Discussion.

Answer: We have organized the discussion in the same order presented in Methods and Results and created subsections in the discussion, in order to improve reading.

6.Overall - You must revise this manuscript shortening certain sections and organizing the manuscript from I to D. Otherwise the results produced cannot be understood by the authors.

Answer: To address this recommendation, we have reduced the text as much as possible, without interfering with data presentation quality and consistency. We have reduced redundancies in the results (data being presented in the text and table) and removed some excerpts throughout the manuscript that we consider less relevant.

7.Language must be clear, correct, and unambiguous. At its current form it is difficult to follow the authors. Please also look into typographical or grammatical errors when your revise the manuscript. You may ask a native speaker to read the manuscript after fixing all the issues indicated.

Answer: we have asked a native speaker to thoroughly review the manuscript.

8.Please follow the PLOS ONE formatting guidelines well before you submit after revision.

Answer: We have done so.

Reviewer #2 comments:
1.The authors present generalized conclusions that are not specific to the Brazilian aspect or timeframe on which this study is based. Key results to support the identified objectives are not highlighted in the abstract or the conclusion (e.g. the influence of climate factors on RSV seasonality and the role of genetic diversity of RSV on disease severity). Clinical severity scores referenced in the abstract and results to support interpretations of the role of viral load and genetic diversity of RSV on disease severity, should be presented in the main tables/figures of the manuscript as opposed to supplemental. The authors should revise these areas and sharpen the focus of their Discussion through reduction to improve readability and presentation of key messages of RSV surveillance in Brazil between 2016-2018 relative to previous observations in Brazil or other parts of the world during the similar timeframe.

Answer: We appreciate the observations. However, some results of this study are not related to a specific location or timeframe. The correlation analyzes between viral load, genetic differences and severity are examples. These results possibly transcends the time and place of the study and, therefore, are not specific to the Brazilian aspect or timeframe.

As recommended, we have included the key results in both abstract and conclusion. We also transformed supplementary table 4 into Table 3. Previous table 3, which presented data on viral load, is now part of Table 4.

Given the different approaches taken in the study, we chose to divide the discussion into topics, in the hope of improving the quality of reading and regarding the discussion length, we removed some less important passages in order to improve the readability and presentation of key messages of the study.

2.The current title (and abstract) fail to address the presented timeframe of RSV

Powered by Editorial Manager® and ProduXion Manager® from Aries Systems Corporation

surveillance or what aspects of “landscape” or “perspectives” the authors are referring to relative to their objectives and results. The authors should consider revision.

Answer: as changing the Title was also a recommendation of Reviewer 1, and to clarify which aspects of the “landscape” the study focused on, we have changed the title to: “Seasonality, molecular epidemiology and virulence of Respiratory Syncytial Virus (RSV): a perspective into the Brazilian Influenza Surveillance Program”. We hope this new title is suitable for both Reviewers.

3.Figure 2 and Figure 3 are out of focus and uninterruptable for review. The authors should revise.

Answer: Figures 2 and 3 were redone to improve quality and readability. We decided to change the size and save the file as .EPS.

4.Line 90-92, 130-133, and 270-276. The authors statement of “seasonal oscillation” (Line 90-92) is not supported by their main observations (Line 270-276) from Fig. 1, and in turn, their statement regarding “creating difficulties for determination of the most appropriate period to start prophylaxis” is not substantiated and is in contrast to their later statement of “recommends the administration of palivizumab from February to July” (Line 359). The authors state (Line 130-133) that “seasonality onset and end were defined as the first and last of 2 consecutive weeks, respectively, when the number of RSV cases exceeded 10% of the number detected during the RSV peak week” and reference Obando-Pacheco et al 2018 [21]. However, Obando-Pacheco et al 2018 states that “the onset of RSV season was defined as the first 2 consecutive weeks when >10% of the total tested samples for respiratory pathogens were positive for RSV. The end of the RSV season was defined similarly as when the proportion of positive RSV tests fell below 10% for 2 consecutive weeks.”. Given the impact of molecular testing on determining RSV seasonality, the authors should revise their analysis and adopt a more accepted threshold for seasonality assessment based on %RSV positive cases as opposed to the number of RSV cases to support a potential interpretation of “seasonal oscillation” (see also Midgley et al. 2017 JID 216(3):345-355).

Answer: We agree with the Reviewer, and, in fact, there was a misinterpretation of season beginning and end definition by Obando-Pacheco et al. (2018). Therefore, we reviewed the data and corrected the analysis. However, there were no changes in season onset in any year, but there were small changes in season end, as described below:

1. End in 2016: from EW 33 to EW 32.
2. End in 2017: from EW 30 to EW 31.
3. End in 2018: from EW 26 to EW 27.

Although the reviewer understood that the data do not support the claim that there was a fluctuation in season period during the study, we would like to point out that in 2016 and 2017 the RSV seasonal period started at epidemiological week (EW) 12 and ended at EW 32 and 31, respectively. In contrast, in 2018 the season was anticipated to EW 3, which is 9 weeks before the start in previous years. Season end was also anticipated to EW 27. There are the reasons why we understand there was an oscillation in the seasonal period during our study. As Palivizumab is administered in five consecutive monthly doses and considering that the first dose should be administered one month before season start, this oscillation may have an impact in the administration of prophylactic drugs.

In order to make this point clearer, we have restructured the discussion paragraphs.

5.Table 1: The authors should revise this Table to provide both numerators and denominators to allow for readability and logical follow with the main text. This will also allow the reader to appropriately follow the statistical assessment employed of relative proportions. In addition, Influenza prevalence is noted in the main text, but not in the corresponding Table 1. The authors should to revise the Table to include all relevant data for the reader.

Powered by Editorial Manager® and ProduXion Manager® from Aries Systems Corporation

Answer: we have revised Table 1, making the requested changes.

6.Line 225-232 and Table 2: The authors should rephrase their statement regarding “clinical features of patients affect by RSV” to better reflect clinical characteristics of patients with SARI, since clinical data are presented for the total 632 patients and the 327 patients with RSV (180 RSV-A and 147 RSV-B). The numbers and percentages in the main text reflect the total population (N=632) and not the population of patients with RSV disease (N=327). The authors should further revise this Table to provide both numerators and denominators to allow for readability and logical follow with the main text. This will also allow the reader to appropriately follow the statistical assessment employed of relative proportions and to distinguish between RSV and everything else. Finally, viral load data in Table 2 is out of place without a (%) and should be included in Table 3 where viral load values are presented.

Answer: Table 2 and the associated text present clinical data only of RSV infected patients. This table had an error in the "Sample number" field, which contained the total number of samples studied (632), however, the analyzes were performed only with the 352 RSV positive children. The error has been corrected. We took the opportunity to correct some fields containing three decimal places, standardizing the values to two decimal places. We have also relocated table 2 viral load data to table 3, as suggested. Finally, we included denominators to facilitate data interpretation.

Minor Comments for Author (Required)

7.Line 17 and 40. The authors are repetitive in their statements in the Background and Conclusion sections of their Abstract regarding “understanding seasonality, genetic features…may support antiviral and vaccine development. The authors should revise the abstract and clarify how the results of this study specifically support antiviral and vaccine development.

Answer: We have eliminated the redundant part from the "Background" and briefly discussed how seasonal period, virulence and genetic diversity can assist in the development and application of vaccines and antiviral drugs.

8.Lines 21, 38, 81, 83, 88, 339-440. Is the Brazilian Influenza Surveillance Program part of WHO’s Global Respiratory Syncytial Virus Surveillance Pilot and/or the Global Influenza Surveillance and Response System (GISRS)? The authors should consider revising for clarity; in particular Lines 338-340 at the start of the Discussion section where both programs are discussed in the context of the objectives of the current study. Recommend that the authors be consistent throughout the manuscript in their reference to the Influenza Surveillance Program as to which this study is based on (ie. National, Brazilian, or just Influenza Surveillance Program are used throughout the manuscript; pick one version and capitalize all words).

Answer: The Brazilian Influenza Surveillance Program is part of WHO’s Global Respiratory Syncytial Virus Surveillance. We chose to use the term “Brazilian Influenza Surveillance Program” with capital words, as suggested.

9.Line 30 and Line 105: What were the remaining 44% of case caused by, all influenza?

Answer: In this study, only RSV and Influenza were tested. In 56% of the cases RSV was detected, the influenza virus was found in 7% of the samples and the remaining 37% cases were undetermined.

10.Line 48: The authors should clarify in the text the source of the “Influenza and other respiratory virus epidemiological reports” as to whether these are from the Brazilian and/or National Influenza Surveillance Program.

Answer: We have followed previous Reviewers’ recommendations to shorten the introduction, and because of that we have removed this text, as explained to the other reviewer.

11.Line 57: The authors should explain the rationale as to why the previously observed significant association between viral load and disease severity should be more carefully studied in the Introduction. The authors later state in the Discussion that the correlation between viral load and disease severity remains controversial (Line 423). The authors are advised to further emphasize that one of the strengths of their study in finding of a lack of correlation between viral load and disease severity is the use of standardized methods for measuring viral load (see Lines 432-442)

Answer: To address that, we have rewritten the Introduction as follows:
"Some studies have evaluated the association between viral load and disease severity, with significant associations [6,7]. However, most of these studies did not use standardized methods of viral load measurement, therefore, this relationship must be more carefully evaluated."

12.Line 60: The authors should revise this sentence to clarify that the context by which "the treatment is based" in referring to RSV since this is new paragraph.

Answer: We have rephrased the sentence to: "RSV treatment is based only […]".

13.Line 72: The authors should supplement reference 15 with a reference that defines the multiple genotypes of RSV-B.
Answer: We have added the study by Trento et al. (2006) which was already mentioned in reference #17 (now reference #14, since due to the removal of some sections to reduce the text, the corresponding references were also removed).

14.Line 78: Reference 15 does not support the statement that understanding RSV genetic diversity will help designing antiviral drugs, diagnostic assays, and vaccines. The authors should revise.

Answer: It is possible to find in reference 15 (now reordered to reference 13) two excerpts that support this statement: "RSV diversity is an important factor that allows for reinfections to occur throughout life and also has implications for design of diagnostic assays, antiviral therapies, and preventive strategies (passive immunization and vaccines)". (in the introduction).
"Genotype classification and assignment is of importance in order to understand the evolution, epidemiology, and clinical presentation of this virus, and has implications regarding the development of vaccines and other preventive interventions." (in the discussion).

15.Fig 1: The y-axis and X-axis should be labeled within the figure.

Answer: Figure 1 has been edited, including caption for the two Y axes and the X axis. Caption is displayed in a text box.

16.Line 126-127: Location of INCAPER should be provided.

Answer: We have included the city, state and country of INCAPER. Please, check the line 145.

17.Line 143: The authors should define in Supplemental Table 1 or elsewhere in the main text what RSV gene the primers and probes used to subtype RSV-A and RSV-B were directed against.

Answer: In the methodology, we include the requested information as follows: "RSV positive samples (i.e. those with cycle threshold [CT] ≤ 40) were subtyped using specific primers and probes to N gene of RSV-A and RSV-B." Please, check the line 105.

18.Line 161: The authors should clarify what they mean by "partial amplification" and by RSV positive samples with Ct values between 30-40 were not subjected or attempted for sequencing.

Answer: Partial amplification in this case refers to the fact only part of the gene was

Powered by Editorial Manager® and ProduXion Manager® from Aries Systems Corporation

	amplified. We have included the approximate sequenced G gene fragment size, as follows:<br>“The partial gene G amplification (about 730 bp) was performed at LVRS/IOC/FIOCRUZ”<br>We have also included the following sentence in bold: “a) cycle threshold (ct) value less than 30, due to the difficulty in sequencing samples with higher ct than this;”<br><br>19.Line 179-180: The authors should provide a reference to the source of their reference sequences.<br><br>Answer: The requested data is already available in supplementary tables 2 and 3. All reference sequences were taken from NCBI Genbank. These supplementary tables contain access numbers, genotypes and collection locations of each sequence.<br><br>20.Line 37, 74, 194, 294, 334, 421, 444, 466: The authors should correct their documentation of the RSV B genotype from BA to BA1 per the accession number provided and documented.<br><br>Answer: The classification into the BA cluster is controversial. We prefer classify as BA. More studies are needed to standardize the RSV nomenclature of genotypes into BA and ON1.
**Additional Information:**	
**Question**	**Response**
**Financial Disclosure**<br><br>Enter a financial disclosure statement that describes the sources of funding for the work included in this submission. Review the submission guidelines for detailed requirements. View published research articles from *PLOS ONE* for specific examples.<br><br>This statement is required for submission and **will appear in the published article** if the submission is accepted. Please make sure it is accurate.	This work was funded by Espirito Santo Research and Innovation Support Foundation (FAPES; https://fapes.es.gov.br/), under project Fapes/CNPq nº 05/2017 and by INOVA Fiocruz Program (https://portal.fiocruz.br/programa-inova-fiocruz), under project VPPCB-008-FIO-18. The funders had no role in study design, data collection and analysis, decision to publish, or preparation of the manuscript.

**Unfunded studies**<br>Enter: *The author(s) received no specific funding for this work.*<br><br>**Funded studies**<br>Enter a statement with the following details:<br>• Initials of the authors who received each award<br>• Grant numbers awarded to each author<br>• The full name of each funder<br>• URL of each funder website<br>• Did the sponsors or funders play any role in the study design, data collection and analysis, decision to publish, or preparation of the manuscript?<br>• **NO** - Include this sentence at the end of your statement: *The funders had no role in study design, data collection and analysis, decision to publish, or preparation of the manuscript.*<br>• **YES** - Specify the role(s) played.<br><br>* typeset	
**Competing Interests**<br><br>Use the instructions below to enter a competing interest statement for this submission. On behalf of all authors, disclose any competing interests that could be perceived to bias this work—acknowledging all financial support and any other relevant financial or non-financial competing interests.<br><br>This statement **will appear in the published article** if the submission is accepted. Please make sure it is accurate. View published research articles from *PLOS ONE* for specific examples.	The authors have declared that no competing interests exist.

Powered by Editorial Manager® and ProduXion Manager® from Aries Systems Corporation

**NO authors have competing interests**<br><br>Enter: *The authors have declared that no competing interests exist.*<br><br>**Authors with competing interests**<br><br>Enter competing interest details beginning with this statement:<br><br>*I have read the journal's policy and the authors of this manuscript have the following competing interests: [insert competing interests here]*<br><br>* typeset	
**Ethics Statement**<br><br>Enter an ethics statement for this submission. This statement is required if the study involved:<br><br>• Human participants<br>• Human specimens or tissue<br>• Vertebrate animals or cephalopods<br>• Vertebrate embryos or tissues<br>• Field research<br><br>Write "N/A" if the submission does not require an ethics statement.<br><br>General guidance is provided below. Consult the submission guidelines for detailed instructions. **Make sure that all information entered here is included in the Methods section of the manuscript.**	This project was approved by the Human Research Ethics Committee of the Health Sciences Center of the Federal University of Espirito Santo (UFES), under the number: 018577/2018; CAAE: 84633518.1.0000.5060. The need for parents or guardians consent was waived by the ethics committee.

Powered by Editorial Manager® and ProduXion Manager® from Aries Systems Corporation

**Format for specific study types**<br><br>**Human Subject Research (involving human participants and/or tissue)**<br>• Give the name of the institutional review board or ethics committee that approved the study<br>• Include the approval number and/or a statement indicating approval of this research<br>• Indicate the form of consent obtained (written/oral) or the reason that consent was not obtained (e.g. the data were analyzed anonymously)<br><br>**Animal Research (involving vertebrate animals, embryos or tissues)**<br>• Provide the name of the Institutional Animal Care and Use Committee (IACUC) or other relevant ethics board that reviewed the study protocol, and indicate whether they approved this research or granted a formal waiver of ethical approval<br>• Include an approval number if one was obtained<br>• If the study involved *non-human primates*, add *additional details* about animal welfare and steps taken to ameliorate suffering<br>• If anesthesia, euthanasia, or any kind of animal sacrifice is part of the study, include briefly which substances and/or methods were applied<br><br>**Field Research**<br><br>Include the following details if this study involves the collection of plant, animal, or other materials from a natural setting:<br>• Field permit number<br>• Name of the institution or relevant body that granted permission	
**Data Availability**<br><br>Authors are required to make all data underlying the findings described fully available, without restriction, and from the time of publication. PLOS allows rare exceptions to address legal and ethical concerns. See the PLOS Data Policy and FAQ for detailed information.	Yes - all data are fully available without restriction

A Data Availability Statement describing where the data can be found is required at submission. Your answers to this question constitute the Data Availability Statement and **will be published in the article**, if accepted.<br><br>**Important:** Stating 'data available on request from the author' is not sufficient. If your data are only available upon request, select 'No' for the first question and explain your exceptional situation in the text box.<br><br>Do the authors confirm that all data underlying the findings described in their manuscript are fully available without restriction?	
**Describe where the data may be found in full sentences. If you are copying our sample text, replace any instances of XXX with the appropriate details.**<br><br>• If the data are **held or will be held in a public repository**, include URLs, accession numbers or DOIs. If this information will only be available after acceptance, indicate this by ticking the box below. For example: *All XXX files are available from the XXX database (accession number(s) XXX, XXX.*).<br>• If the data are all contained **within the manuscript and/or Supporting Information files**, enter the following: *All relevant data are within the manuscript and its Supporting Information files.*<br>• If neither of these applies but you are able to provide **details of access elsewhere**, with or without limitations, please do so. For example:<br><br>*Data cannot be shared publicly because of [XXX]. Data are available from the XXX Institutional Data Access / Ethics Committee (contact via XXX) for researchers who meet the criteria for access to confidential data.*<br><br>*The data underlying the results presented in the study are available from (include the name of the third party*	The sequences produced here were deposited on GenBank platform, under the accession<br>203 number MW026969–MW027004 and MW030961-MW030981, and in GISAID<br>204 platform, under the accession number EPI_ISL_549271- EPI_ISL_549327.

Powered by Editorial Manager® and ProduXion Manager® from Aries Systems Corporation

and contact information or URL).<br>• This text is appropriate if the data are owned by a third party and authors do not have permission to share the data.<br><br>* typeset	
Additional data availability information:	

Cover Letter

Universidade Federal do Espírito Santo (UFES)
Pró-Reitoria de Pesquisa e Pós-Graduação (PRPPG)
Programa de Pós-graduação em Biotecnologia (PPGBiotec)
Núcleo de Genética Humana e Molecular (NGHM)

Vitoria, January 25th, 2021.
To: Editor-in-chief of Plos One

Dear Editor,

After carefully addressing all observations made by the peer reviewers, we are happy to submit the revised version of our study now entitled: "Seasonality, molecular epidemiology and virulence of Respiratory Syncytial Virus (RSV): a perspective into the Brazilian Influenza Surveillance Program" for consideration by Plos One, as a primary research article. RSV leads the causes of respiratory infections in infants and small children, been responsible for millions of hospitalizations and thousands of deaths worldwide. In this article, we have used the Brazilian Influenza Surveillance Program to investigate the prevalence of RSV in children under 3 years old in Espírito Santo State, Southeast Brazil, between 2016 and 2018. We explored RSV seasonality over time and investigated whether climatic factors influence RSV circulation. We have also sequenced G gene to explore the circulating genotypes from both subtypes (RSVA and RSVB). G gene phylogenies allowed us to understand how the strains found in this study are related to those that circulate worldwide. Clinical data from patients enabled us to infer that RSVA is responsible for the development of a more severe disease than RSVB. Finally, we performed a normalized viral load study that demonstrated an absence of association between severity and viral titer. The implementation of RSV surveillance at a global level has become one of the World Health Organization's priorities, which, in 2017, started the pilot project to assess the suitability of the use of the Global Influenza Surveillance and Response System in RSV surveillance. We believe that our study enriches the knowledge regarding multiple aspects of RSV and may help to establish a global surveillance network.

We are hopeful that the manuscript is now suitable for publication.

Sincerely,

Lucas Alves Vianna
E-mail: lucasavianna@gmail.com

Manuscript Click here to access/download;Manuscript;Manuscript.docx

Full Title: Seasonality, molecular epidemiology and virulence of Respiratory Syncytial Virus (RSV): a perspective into the Brazilian Influenza Surveillance Program

Short Title: Landscape of Respiratory Syncytial Virus (RSV)

Lucas A. Vianna[1,2], Marilda M. Siqueira[3], Lays P. B. Volpini[4], Iuri D. Louro[2], Paola C. Resende[3]

[1] Central Laboratory of Public Health of the State of Espirito Santo, Vitoria, Espirito Santo, Brazil.

[2] Nucleus of Human and Molecular Genetics/ Federal University of Espirito Santo/ UFES, Vitoria, Espirito Santo, Brazil.

[3] Laboratory of Respiratory Viruses and Measles, National Influenza Center (NIC)/ World Health Organization (WHO), Oswaldo Cruz Institute, Oswaldo Cruz Foundation, Rio de Janeiro, Rio de Janeiro, Brazil.

[4] Virology & Infectious Gastroenteritis Laboratory / Federal University of Espirito Santo /UFES, Vitoria, Espirito Santo, Brazil.

Abstract

Background: Respiratory Syncytial Virus (RSV) is the main cause of pediatric morbidity and mortality. The complex evolution of RSV creates a need for worldwide surveillance, which may assist in the understanding of multiple viral aspects.

Objectives: This study aimed to investigate RSV features under the Brazilian Influenza Surveillance Program, evaluating the role of viral load and genetic diversity in disease severity and the influence of climatic factors in viral seasonality.

Methodology: We have investigated the prevalence of RSV in children up to 3 years old with severe acute respiratory infection (SARI) in the Espirito Santo State (ES), Brazil,

from 2016 to 2018. RT-qPCR allowed for viral detection and viral load quantification, to evaluate association with clinical features and mapping of local viral seasonality. Gene G sequencing and phylogenetic reconstruction demonstrated local genetic diversity.

Results: Of 632 evaluated cases, 56% were caused by RSV, with both subtypes A and B co-circulating throughout the years. A discrete inverse association between average temperature and viral circulation was observed. No correlation between viral load and severity was observed, but children infected with RSV-A presented higher clinical severity score (CSS) median, stayed longer in the hospital, required more intensive care and ventilatory support than those infected by RSV-B. Regarding RSV diversity, some local genetic groups were observed in the main genotypes circulation RSV-A ON1 and RSV-B BA, with strains showing modifications in the G gene amino acid chain.

Conclusion: Local RSV studies using the Brazilian Influenza Surveillance Program are relevant because they can reveal useful information, contributing to the global RSV surveillance. Understanding seasonality, virulence and genetic diversity can support the suitability of future antiviral drugs and vaccines and assist in the administration of prophylactic strategies.

Introduction

Respiratory Syncytial Virus (RSV) is the most common pathogen associated with acute respiratory tract infections (ARTI), being the main cause of bronchiolitis and pneumonia in infants and small children [1].

RSV infection can cause a range of symptoms, varying from mild upper respiratory tract illness to severe lower respiratory tract infection [2]. The reason for different outcomes is still unclear, however it can be related to the underline conditions, genetic or acquired host factors, and/or viral characteristics [3,4]. Some studies have

evaluated the association between viral load and disease severity with significant associations [4,5]. However, most of these studies did not use standardized methods of viral load measurement, therefore, this relationship must be more carefully evaluated. The understanding of RSV infection viral load may be a tool to establish its relationship with disease progression, severity, clinical outcome and drug intervention timeframe [6].

RSV treatment is based only in supportive care and infection prevention is limited to passive immunoprophylaxis (Palivizumab) and case isolation [2]. No licensed RSV vaccine is available, but some promising candidates are currently in development and in advanced clinical trial phases [7].

RSV strains can be classified into two serogroups: RSV-A and RSV-B [8]. The potential virulence attributed to a specific group remains controversial: some authors have pointed RSV-A [9,10] or RSV-B [11] as the most virulent subtype, other studies have not found significant differences between them [12].

Multiple genotypes were described for RSV-A and RSV-B, based on gene G second hypervariable region (HVR-2) [13,14]. In the past two decades important shifts occurred with the emergence of new RSV-A and RSV-B genotypes: RSV-A ON1 containing a duplication of 72 nucleotides, and RSV-B BA with a duplication of 60 nucleotides in the HVR-2 gene G [14,15]. These genotypes replaced previous ones and have spread globally. Understanding their genetic diversity may reveal the virus's ability to cause re-infections throughout life, and help designing antiviral drugs, diagnostic assays and vaccines [13].

In 2017, the World Health Organization (WHO) launched the Global Respiratory Syncytial Virus Surveillance Pilot in order to test the feasibility of using the Global Influenza Surveillance and Response System (GISRS) for RSV surveillance without adversely affecting influenza surveillance [16]. This pilot study results from the global

concern about RSV impact on public health. Brazil, one of four countries in the Americas included in the pilot, has a remarkable respiratory virus surveillance program, however, more data are required for a better understanding of factors such as RSV circulation, evolution, and pathogenicity. In this study, we used the Brazilian Influenza Surveillance Program to analyze the local prevalence of RSV in children with SARI and to evaluate which factors are potentially associated with disease severity. We also explored the viral seasonality and investigate the influence of climatic factors in the circulation. Finally, we conducted a phylogenetic study to understand how the local genetic diversity of RSV behaves with that observed in the rest of the world.

Materials and methods

Population sampling, study period and location

This study is a retrospective investigation of respiratory samples (nasopharyngeal secretions, tracheal and bronchoalveolar aspirate and bronchoalveolar lavage) collected from the Brazilian Influenza Surveillance Program during 34 months (March 7th, 2016, to December 14th, 2018). A total of 632 samples collected from pediatric patients (from 0 to 36 months old) classified as SARI, residents of 60 municipalities in the ES State, were enrolled in this study. ES state is located in southeastern Brazil and it has a territory of 46.074,447 km^2, with a population of approximately 4.1 million inhabitants [17]. These samples were screened by real-time RT-qPCR for RSV and Influenza A/B at the ES Central Public Health Laboratory (LACEN/ES), one of 26 Brazilian laboratories that integrate the Brazilian Ministry of Health Influenza Surveillance Program.

RSV and Influenza detection and subtyping

Nucleic acids were extracted from respiratory samples using the PureLink™ Viral RNA/DNA Mini Kit (Invitrogen®, Thermo Fisher Scientific©), according to manufacturer's protocol. All samples were tested initially for Influenza A and B in a TaqMan® one-step real time RT-PCR (RT-qPCR) assay with primers and probes specific for influenza (CDC, USA), according to manufacturer's recommendations. Additionally, RT-qPCR assay was performed to identify positive RSV cases using GoTaq® Probe 1-Step RT-qPCR Kit (Promega, Madison, WI, EUA). RSV positive samples (*i.e.* those with cycle threshold [CT] ≤ 40) were subtyped using specific primers and probes to RSV-A and RSV-B N gene. In parallel, Ribonuclease P RNA (RNase P) was tested as an internal control for each sample and all batches had an RNA extraction negative control (MOCK) and a PCR negative control (NTC). All primers and probes are described in the **S1 Table**.

Clinical and epidemiological data collection

Clinical and epidemiological data were retrieved mainly from the Information System of Diseases of Compulsory Declaration of Notification Illness (SINAN) and, in some cases – where the SINAN file was incomplete – we assessed patients' Medical Records to fill missing information. The main information recovered from SINAN were: 1) clinical outcome (recovered or death); 2) hospitalization length; 3) oxygen administration need and type (invasive or not invasive), 4) intensive care unit (ICU) need and length; 5) clinical characteristics (fever, cough, dyspnea, O_2 saturation, respiratory distress, comorbidities) and 6) epidemiological and demographical features (age, race, town or area of residence).

We have used the Brazilian Ministry of Health definition of SARI, that is: hospitalized patient with fever and cough or sore throat and presenting dyspnea or O_2 saturation <95%, or respiratory distress [18]. A Clinical Severity Score (CSS) was adapted from Martinello

et al. [19]. The scale ranged from 0 to 5 points, where 0 was the mildest condition and 5 the most severe. ICU admission, hospitalization length ≥5 days, oxygen saturation ≤95% and oxygen therapy noninvasive methods accounted for 1 point each. Two points were assigned for mechanical ventilation.

Viral load quantification

RSV viral load was determined by RT-qPCR using a protocol adapted from Álvarez-Argüelles *et al*. [20], including a synthetic *β-globin* dsDNA as a template. To quantify the RSV copy number, expressed in copies per cell (c/c), we designed a dsDNA containing the annealing regions of RSV primers and probe, as well as the upstream and downstream regions (150 bp). This synthetic DNA was incorporated into a pMA-T plasmid, which was used in the RT-qPCR. Standard curves for absolute quantification of RSV and *β-globin* gene were generated by 10-fold serial dilutions (10^6-10^1 copies of genome), in triplicate. RSV primers, probe and the thermal cycling protocol used were the same used in the diagnostic phase. *β-globin* primers and probe are listed in **S1 Table**. All amplification assays were carried out in the ABI 7500 equipment (Applied Biosystems, Foster City, CA, USA). Viral load status was compared with different clinical features and epidemiological data.

Climate data collection

Climate data (precipitation, temperature and humidity) of five cities – representatives from the different geographic regions of the state – were daily collected and kindly provided by the Capixaba Institute of Research, Technical Assistance and Rural Extension (INCAPER), Vitoria, Espirito Santo, Brazil. The weekly average was accessed by assembling daily data from all collection sites for each epidemiological week (EW).

The definition of RSV epidemic period was based on a previously described protocol [21], which considers RSV outbreak onset, peak and end. Seasonality onset was defined as the first of 2 consecutive weeks when ≥10% of tested samples for respiratory pathogens were positive for RSV. Similarly, RSV season end was defined when the proportion of positive RSV tests fell below 10% for two consecutive weeks. Peak was determined as the week when the maximum number of RSV positive cases occurred [21].

Partial amplification and sequencing of glycoprotein gene

RSV-A and RSV-B positive samples were selected to be sequenced based on the following criteria: a) cycle threshold (ct) value less than 30, due to the difficulty in sequencing samples with ct higher than this; b) representativeness by collection date; c) distinct clinical outcomes; and d) different viral load values.

The partial gene G amplification (about 730 bp) was performed at LVRS/IOC/FIOCRUZ, the National Influenza Center, by conventional RT-PCR, using the QIAGEN OneStep RT-PCR Kit (Qiagen) and a pair of primers (**S1 Table**) for each subtype. The reverse transcription was performed at 55°C for 30 minutes and the cDNA was amplified by PCR (40 cycles of 94°C/30 seconds, 60°C /1 minute, 72°C/1 minute and a final extension at 72°C/10 minutes). Amplification was confirmed in a 1% agarose gel. DNA was purified using ExoSap-IT Kit (Affymetrix, Inc., USA) and submitted to sequence reaction using BigDye™ Terminator v3.1 Cycle Sequencing Kit (Applied Biosystems, Foster City, CA, USA) and primers at 3.2 µmolar. The reads were obtained in the ABI 3130XL Genetic Analyzer (Applied Biosystems). Consensus sequences were built from electropherograms comparison with a reference sequence in the software Sequencher 5.1 [22]. The adopted nomenclature pattern hereon was "hRSV subtype/country/ES-sample number/year."

RSV genotyping and gene G phylogenetic reconstruction

RSV-A and RSV-B gene G DNA sequences (711 bp and 726 bp, respectively) were used to reconstruct phylogenetic relationships. Genotyping was based on gene G HVR-2, using RSV-A and RSV-B sequences (336 bp and 318 bp, respectively). Reference sequences of previously described genotypes are shown in **S2 Table**. Additionally, to place our sequences in a global context we performed a BLAST search (Basic Local Alignment Search Tool), available at https://blast.ncbi.nlm.nih.gov/Blast.cgi. These sequences (**S3 Table**) were labeled with country of origin and collection year, and those with more than 99.5% genetic similarity using CD-HIT tool (http://weizhongli-lab.org/cd-hit/servers.php) were removed from the final dataset. Alignments were conducted using Muscle algorithm, via MEGA 6.0 software [23] and when necessary they were adjusted manually. The phylogenetic trees were constructed using the Maximum Likelihood (ML) method, complete deletion for gap or missing data treatment and 1000 replicates of bootstrap probabilities tools integrated within Mega 6.0. General Time Reversible + Gamma (GTR+G) was the nucleotide substitution model elected for all analysis on JModelTest software, with an exception for RSV-A, which Tamura-Nei + Gamma (TrN+G) was the substitution model nucleotide indicated for the analysis [24]. Average pairwise distance (p-distance) was calculated in Mega 6.0. Amino acid comparisons with the reference sequences of ON1 (JN257693) and BA (AY333364) were performed using MEGA 6.0 software to map the changes in Brazilian samples amino acids.

Statistical treatment

Statistical analyses were performed using SPSS 20.0 (SPSS, Inc., Chicago, IL) and R v.3.4.4 software. Chi-square, Fisher exact, Mann–Whitney and Kruskal Wallis were used

whenever appropriated. To test the association between climate data and RSV circulation we performed the Spearman correlation test. A p-value of less than 0.05 was considered statistically significant.

Data availability

The sequences produced here were deposited on GenBank platform, under the accession number MW026969–MW027004 and MW030961-MW030981, and in GISAID platform, under the accession number EPI_ISL_549271- EPI_ISL_549327.

Ethics Statement

This project was approved by the Human Research Ethics Committee of the Health Sciences Center of the Federal University of Espirito Santo (UFES), under the number: 018577/2018; CAAE: 84633518.1.0000.5060. The need for parents or guardians consent was waived by the ethics committee.

Results

RSV clinical and epidemiological data

A total of 632 respiratory samples collected from children under 3 years-old were tested by RT-qPCR for Influenza A, Influenza B and RSV~~, being RSV~~ the most prevalent pathogen found in these samples (56%; 352/632) (**Table 1**). From RSV positive cases, ~~54~~% (180/352) were RSV-A, 147 (~~44~~%) were RSV-B, co-detections with both subtypes were found in 5 cases (~~1.5~~%). Twenty samples could not be subtyped. Influenza frequency was 7.4% (47/632), being 74.4% (35/47) Influenza A H1N1 pdm09, 14.9%

(7/47) H3N2 and 10.6% (5/47) Influenza B. Male gender was slightly more affected by RSV (n=182; 52%) and median age was 4 months old (1-11.0 interquartile range; IQR). Of positive cases, 99.7% (351/352) were classified as SARI and 14 deaths (4%) were reported. Most children were brown (54%), according to the self-racial classification filled out by children's legal guardian.

Table 1. Number of tested samples, RSV positivity, subtype prevalence and demographic data from each year and the whole study period. Statistical tests were performed to verify the statistical significance of data differences among years. Statistically significant values are highlighted in bold.

	**2016 n (%)**	**2017 n (%)**	**2018 n (%)**	***p-value***	**2016-18 n (%)**
**General data**					
Sample n°	251/632 (0.40)	135/632 (0.21)	246/632 (0.39)	-	632/632 (1)
RSV +	155/251 (0.62)	80/135 (0.59)	117/246 (0.48)	**0.003**	352/632 (0.56)
RSV -	96/251 (0.38)	55/135 (0.41)	129/246 (0.52)		280/632 (0.44)
Flu +	27/632 (0.11)	6/135 (0.04)	14/246 (0.06)	-	47/632 (0.07)
RSV+ deaths	6/251 (0.04)	5/135 (0.06)	3/246 (0.03)	0.42[1]	14/352 (0.04)
Subtyped samples	141/251 (0.91)	78/135 (0.98)	113/246 (0.97)	-	332/352 (0.94)
**Subtypes**					
RSV-A	58/141 (0.41)	14/78 (0.18)	108/113 (0.96)	**<0.001[1]**	180/332 (0.54)
RSV-B	80/141 (0.57)	63/78 (0.81)	4/113 (0.04)		147/332 (0.44)
RSV-A and RSV-B	3/141 (0.02)	1/78 (0.01)	1/113 (0.01)		5/332 (0.02)
**Demographic data (RSV+)**					
Median age (months)	4 (1-12.0)	4 (1-10.5)	3 (1-8.0)	0.793	4 (1-11.0)
**Gender**					
Male	72/155 (0.46)	49/80 (0.61)	61/117 (0.52)	0.098	182/352 (0.52)
Female	83/155 (0.54)	31/80 (0.39)	56/117 (0.48)		170/352 (0.48)
**Race**					
White	61/122 (0.50)	26/66 (0.39)	31/97 (0.32)	**0.039[1]**	118/285 (0.41)
Brown	53/122 (0.43)	38/66 (0.58)	64/97 (0.66)		155/285 (0.54)

Black	7/122 (0.06)	1/66 (0.02)	2/97 (0.02)		10/285 (0.04)
Yellow	1/122 (0.01)	1/66 (0.02)	0		2/285 (0.01)
Undeclared	33	14	19		66

[1] Fisher's exact test.

Table 2 shows patients' clinical features by RSV+ and subtypes. The most frequent symptom reported was cough (93%, 318/341), followed by respiratory distress (88%, 269/307), and fever (86%, 288/336). 74% (252/342) of children needed oxygen therapy and 38% (95/252) of these required mechanical ventilation. The median hospitalization time was 8 (6-14 IQR) days. Intensive care was needed for 61% (202/333) of patients and the median number of days in ICU was 6 (3-10 IQR).

Table 2. Summary of clinical and epidemiological data by RSV+ and each subtype. Statistically significant values are highlighted in bold.

	**RSV+ 2016-2018**	**Subtypes**		
	**n (%)**	**RSV-A**	**RSV-B**	***p-value***
	**Demographic profile**			
Sample number	352	180	147	
Deaths RSV+	14/352 (0.04)	3/180 (0.02)	8/147 (0.05)	0.07[1]
**Age**				
Median age: months (IQR)[2]	4 (1-11)	4 (1-10.0)	4 (1-12.5)	0.78
**Gender**				
Male (%)	182/352 (0.52)	92/180 (0.51)	78/147 (0.53)	0.725
Female (%)	170/352 (0.48)	88/180 (0.49)	69/147 (0.47)	
**Clinical profile**				
Fever	288/336 (0.86)	147/174 (0.84)	124/139 (0.89)	0.223
Cough	318/341 (0.93)	162/176 (0.92)	134/142 (0.94)	0.418
Dyspnea	251/331 (0.76)	135/172 (0.78)	97/136 (0.71)	0.148
O_2 saturation ≤ 95%	169/277 (0.61)	101/150 (0.67)	56/109 (0.51)	**0.009**
Respiratory distress	269/307 (0.88)	154/167 (0.92)	96/120 (0.80)	**0.002**

O_2 Therapy	252/342 (0.74)	138/177 (0.78)	98/143 (0.68)	
Invasive	95/252 (0.38)	56/138 (0.41)	33/98 (0.34)	0.092
Noninvasive	157/252 (0.62)	82/138 (0.59)	65/98 (0.66)	
Intensive Care	202/333 (0.61)	113/168 (0.67)	78/142 (0.55)	**0.03**
Median hospitalization days	8 (6-14)	9 (6-15)	8 (5-14.0)	0.15
Median days in Intensive Care	6 (3-10)	7 (4-11.0)	6 (3-9)	0.13

[1] Fisher's exact test.

[2] IQR: interquartil range.

When compared to RSV-B, patients affected by RSV-A showed a higher frequency of respiratory distress (92% vs 80%, p=0.002), more often manifested O_2 saturation ≤95% (67% vs 51%, p=0.009) and higher requirement for intensive care (67% vs 55%, p=0.03). Our data also indicate that patients affected by RSV-A stayed one day longer in hospital and in intensive care units than those affected by RSV-B, however these data were not statistically significant. Lastly, RSV-A viral load showed more than twice the number of virus copies per cell (median=57.41 copies/cell) than RSV-B (median=27.35 copies/cell). RSV-A CSS median was 4 and RSV-B's was 3, and children infected by RSV-A were most frequently classified in higher severity scores than those infected by RSV-B (**Table 3**). S4 Table shows the difference in severity by ethnicity.

Table 3. Clinical Severity Score (CSS): score varied between 0 and 5. Higher values represented more severe illness. Need for ICU, O_2≤95%, length in hospital >5 days and requirement of O_2 therapy accounted for 1 point each. Need for mechanical ventilation accounted for 2 points. Patients infected with RSV-A were most commonly classified into the most severe scores. Difference between viral loads was not related to severity, but there was no statistical significance.

Clinical Severity Score (CSS)

**CSS**	**RSV-A n (%)**	**RSV-B n (%)**	***p-value***	**Viral load median (IQR)**	**n**	***p-value***
**0**	1 (1%)	10 (15%)		54.06 (6.12-603.61)	8	
**1**	8 (8%)	8 (12%)	**0,003**	217.41 (96.38-370.56)	9	0.089
**2**	19 (20%)	11 (17%)		41.18 (6.53-112.59)	16	

**3**	14 (15%)	15 (23%)		17.31 (6.33-125.40)	14
**4**	26 (27%)	9 (14%)		12.05 (4.32-36.63)	9
**5**	28 (29%)	13 (20%)		11.81 (1.14-54.24)	18

Viral load

A total of 156 (44%) samples were submitted to the viral load analysis (**Table 4**). According to age, median viral load was higher in children with 4 to 6 months old (63.0 cop/cell, p=0.007). Regarding patient clinical conditions, we found lower viral load in patients with fever (26.15 cop/cell) than those without it (111.29 cop/cell; p=0.00) and higher viral load (70.24 cop/cell) in patients without need for oxygen therapy (22.69 cop/cell; p=0.02). Deceased patients had lower viral load (2.80 cop/cell; p=0.02) in comparison to the others (37.96 cop/cell). Although lacking statistical support (p=0.089), a noteworthy observation is the tendency of lower viral load in patients with elevated CSS. The viral load analysis was performed regardless of time between symptom onset and date of collection, which, in theory, could alter the interpretation. However, of 156 samples used to measure viral titers, only 26 (16%) were collected 7 days after symptoms onset. A segmented analysis revealed very similar results when only samples collected until the 7th day of symptom onset were used. Therefore, we prefer to keep late collection patients in the analysis.

Table 4. Comparison of viral load values between gender, race, age, outcome and clinical condition. Statistically significant p-values are highlighted in bold.

**Demographic data**				
**Parameter**		N	**Median (IQR)**	***p-value***
**Gender**	Male	78	51.40 (8.13-265.31)	0.08
	Female	78	24.63 (4.46-88.29)	
**Race**	White	52	54.30 (7.38-207.94)	0.09[1]
	Brown	63	16.70 (4.78-84.83)	
	Black	4	52.83 (0.30-241.27)	
**Age (months)**	0-3	86	51.40 (6.12-152.90)	**0.007[1]**
	4-6	22	63.09 (32.12-211.67)	

	7-12	21	39.29 (2.32-236.91)	
	>12	26	7.77 (1.72-36.92)	
**Outcome**	Recovery	130	37.96 (6.72-122.71)	**0.02**
	Death	7	2.80 (0.04-21.49)	
**Clinical data**				
**Fever**	Yes	121	26.15 (4.33-104.46)	**0.00**
	No	27	111.29 (51.80-408.21)	
**Cough**	Yes	144	41.53 (4.86-148.15)	0.59
	No	7	11.52 (7.98-106.29)	
**Dyspnea**	Yes	106	37.96 (3.91-154.88)	0.69
	No	40	42.05 (8.58-120.16)	
**O_2 saturation ≤ 95%**	Yes	71	26.41 (3.95-150.65)	0.40
	No	51	50.16 (8.36-196.81)	
**Respiratory distress**	Yes	115	39.29 (4.78-150.13)	0.27
	No	18	75.69 (12.66-214.26)	
**Days of hospitalization**	1-4	20	79.36 (11.10-245.08)	0.20[1]
	5-8	49	39.45 (11.89-176.21)	
	>8	54	24.42 (4.08-78.04)	
**Ventilatory support**	*No*	48	70.24 (11.41-342.96)	**0.02**
	Yes (total)		22.69	
	Yes - noninvasive	65	26.41 (6.26-105.11)	0.35
	Yes - invasive	40	17.31 (3.95-68.70)	
**Intensive Care**	Yes	82	30.01 (4.41-113.44)	0.73
	No	67	39.29 (6.90-154.61)	
**Days of Intensive Care**	1-4	20	34.74 (3.60-226.28)	0.547[1]
	5-8	16	16.27 (2.09-106.22)	
	>8	24	36.24 (9.10-106.65)	
**Days of symptom until collect**	0-3	51	36.63 (5.99-220.48)	0.19[1]
	4-6	67	39.98 (7.65-135.24)	
	7-9	24	19.98 (0.53-77.39)	
	>9	12	10.45 (3.92-50.98)	
**Subtype**	RSV-A	64	57.41	**0.03**
	RSV-B	76	27.35	

[1] Kruskal-Wallis test.

[2] IQR: interquartil range.

Viral seasonality and climatic analysis

In 2016 and 2017, RSV season started in the 12th EW (March, early fall season), peaked between the 16th–20th EW and ended in the winter season, between the 31th–32th EW (**Fig 1**; **S5 Table**). In 2018, the beginning of RSV seasonality was anticipated, with the first cases occurring in 3th EW, (January, in the middle of summer). Peak happened in

14th EW and the end occurred in 27th EW. Thus, the RSV seasonal period in 2016, 2017 and 2018 lasted 20, 19 and 24 weeks, respectively.

Fig 1. Circulation of RSV-A and RSV-B between 2016 and 2018 in Espirito Santo State. The X-axis shows the epidemiological weeks (EW) for each year. The primary Y axis displays the number of positive cases for each of the subtypes and the secondary Y axis shows the values of the climatic variables. The gray zone indicates the total number of samples tested in each EW.

Precipitation rate and relative humidity percentage have not been shown to influence the distribution of RSV cases by Spearman's correlation test (p=0.55 and 0.11, respectively). The mean temperature, however, showed a minor and inverse correlation with RSV infections (-0.16; p=0.05).

Although RSV-A and RSV-B co-circulated in each year, it is noteworthy that the subtype distribution changed over the years. In 2016, RSV-B predominated (n=80; 58%) over RSV-A (n=58; 42%). In 2017 this difference increased, and RSV-B was responsible for 82% of the cases (n=63). Finally, in 2018, there was a shift in this pattern and almost all RSV cases were caused by RSV-A (n=108; 96%).

Phylogeny of RSV and genetic analysis.

The phylogenetic reconstructions revealed 36 RSV-A classified such as GA2. ON1 genotype, and 21 RSV-B, BA genotype, based on 2nd HVR (**S1 and S2 Figs**). Some local genetic groups of both genotypes and a slightly higher diversity among the RSV-A strains (p-distance=1.8%) were observed in comparison to RSV-B (p-distance=1.6%) (**Figs 2 and 3**).

Fig 2. RSV-A phylogenetic tree. The tree was built using maximum likelihood method on MEGA 6.0 software from a MUSCLE alignment of G gene sequences of 711 bp. Previously published sequences from known genotypes were retrieved from NCBI database. The ES strains that were sequenced in this work are highlighted in bold. Numbers from 1 to 5 within the squares indicate the patients' CSS. The cross indicates

patients who died due to RSV infection. The stars indicate viral load, categorized by color (in copies per cell).

Fig 3. RSV-B phylogenetic tree. The tree was built using maximum likelihood method on MEGA 6.0 software from a MUSCLE alignment of G gene sequences of 726 bp. Previously published sequences from known genotypes were retrieved from NCBI database. The ES strains that were sequenced in this work are highlighted in bold. Numbers from 1 to 5 within the squares indicate the patients' CSS. The cross indicates patients who died due to RSV infection. The stars indicate viral load, categorized by color (in copies per cell).

RSV-A ES Brazilian strains, from 2016 to 2018, are clustered with strains that circulated in North America, South America, Asia, Africa and Oceania, from 2011 to 2018. A Brazilian main local cluster BR.1 (L142S, L274P, Y304H and T320A) was observed circulating from 2016 to 2018 in ES state. However two new subclusters, BR.1.1 (E106G,) and BR.1.2 (N103T, S144I, E224V, S270P and/or P298L) were detected co-circulating in ES state in 2018. Amino acid substitutions compared with the RSV-A GA2.ON1 reference strain (JN257693) can be observed in the **S6 Table**. The average CSS inside BR.1 cluster was 2.84, while the average in the rest of BR strains was 3.78, showing that BR.1 cluster may be associated with lower severity disease than the other strains. Viral load seemed to be higher on BR.1 strains when compared to other Brazilian strains.

RSV-B gene G phylogenetic reconstruction (**Fig 3**) revealed that Brazilian strains from 2016 to 2018 belonged to a cluster containing global strains circulating from 1999 to 2018. ES Brazilian strains were distributed through this main cluster and presented punctual amino acid substitutions, some of them with potential loss of O-glycosylation, such as T229N and/or S287F (strains from 2017). Inside the main cluster, some local subclusters were observed, such as BR.1 (S101G loss glycosylation site, P217L and T248A loss glycosylation site) e BR.2 (G136S and S269P) in samples from 2016, reveling the large diversity among RSV-B virus circulating in the ES State during that year. Additionally two strains from 2017 presented an insertion of tree nucleotides at

codon 228. All these amino acid substitutions compared with the RSV-B BA reference strain (AY333364) are described in **S7 Table**. CSS and viral load data were unavailable for most of RSV-B sequences, therefore, we could not compare those data with the genetic strains observed.

Discussion

In this paper we investigated RSV features using Brazilian Influenza Surveillance Program and addressed some RSV issues listed in the WHO global RSV surveillance pilot objectives [16], such as RSV burden in hospitalized children and mapping of local seasonality. Additionally, we describe the molecular characteristics of gene G and it revealed RSV-A and RSV-B local clusters co-circulating in Brazil.

RSV is prevalent in Brazilian children with SARI.

RSV prevalence in different Brazilian regions is highly diverse, ranging from 7.7% to 77.6% [25–27]. In the ES state, from 2016 to 2018, the prevalence in hospitalized children up to 3 years-old was 56%. These differences are probably related to the use of diverse methods of RSV detection (*e.g.* RT-PCR or immunofluorescence) or patient inclusion criteria (*e.g.* age, symptoms, period of the year). During 1997-98 season, Checon *et al.* found a prevalence of 28% in the capital of ES State [27]. This lower prevalence in comparison to our study can be attributed to the less sensitive method used (immunofluorescence) and a broader target population age (children ≤ 5 years old).

In our study, the median age of four months in hospitalized children with RSV confirms the higher prevalence in children younger than one year old [2], which justifies why RSV vaccine candidates are aiming to protect, primarily, infants and young children [7].

The subtype but not the viral load appears to be associated with disease severity.

RSV infection can cause a range of clinical outcomes [2], but factors attributed to a worst outcome remain unclear [3,4]. Several studies have shown that male gender is a risk factor for a RSV infection [2], while others have not observed such a connection [28]. Although without statistical significance, we observed that male children were slightly more affected than female, which could support the hypothesis that male children are at higher risk. Nevertheless, the CSS median was three for both genders.

Some evidences suggest that Afro-descendant children are more resistant to RSV infection than white children. [28]. In contrast, in our study, brown and black children stayed longer periods in hospital and ICU, had lower oxygen saturation and used oxygen therapy and mechanical ventilation more often than white children (**S4 Table**). In addition, the prevalence of SARI caused by RSV was higher in Brazilian brown children (54%). However, it is important to consider that miscegenation of Brazilian population shows a high degree of ancestry heterogeneity both for mitochondrial and genomic DNA [29]. According to the Brazilian Institute of Geography and Statistics (IBGE), blacks and browns make up for the majority of the poverty group in Brazil [30]. RSV lethality appears to be, at least in part, associated with socio-economic conditions, since the lethality of RSV infection in developing countries is seven fold the rate in industrialized countries [31]. Low income implies less access to basic conditions and health services, which may explain our findings.

Although some authors have found no correlation between subtypes and disease severity [32,33], many others revealed RSV-A as the most virulent subtype [9,10,12,34,35]. We have found that children hospitalized due to SARI with RSV-A

infection revealed a higher clinical score index (CSS median=4) – therefore, a more severe disease – when compared to those with RSV-B (CSS median=3). Children infected by RSV-A required O_2 therapy more often than those infected by RSV-B and, of all children who needed O_2 therapy, those affected by subgroup A needed mechanical ventilation more frequently. Although these data did not have statistical support, other studies found the same connection [9,10]. Our data also shows that children infected by subgroup A required ICU more often (p=0.03) and remained hospitalized and in ICU a day longer, on average, when compared to those infected by RSV-B, which is agreement with previous studies [35,36]. Notwithstanding, we highlight that only one genotype was found for each subtype (ON1 and BA), thus, those differences in severity could be a consequence of differences in genotypes virulence, rather than subtypes.

The correlation between disease severity and viral load remains controversial. While several authors have shown that the severity of the infection follows the viral load [37,4,5,38], others have not [7,12,33]. Some studies found associations between viral load and symptom frequency , but not severity itself [39,40]. Viral load measurement methods are widely variable between studies: some authors use plaque assay [4] or semi-quantitative analyses, such as ct [5,7,32], others use quantitative methods [38–41]. Moreover, most studies that use quantitative methods do not normalize the measurements. Respiratory samples are naturally heterogeneous and the collection technique can influence viral genome concentration [38].

In this study we used a standardized method for measuring viral load. Interestingly, we found lower viral load in patients with fever (p=0.00), with need of ventilatory support (p=0.02) and in those who died (p=0.02). Our data are in conflict with previous studies that demonstrated a positive association between viral load and the presence of cough, fever [39] and the need for intubation [37]. However, two recent

studies observed higher viral load in less severe RSV disease [42,43]. Piedra *et al.* observed a positive correlation between viral load and mucosal concentration of proinflammatory cytokines that may suggest that high RSV loads can protect from disease progression due to the promotion of an early robust innate immune response [42,43]. Conflicting results between studies could be attributed to the different methods used to calculate viral load, various study designs and indicators of disease severity.

The seasonal period of RSV may fluctuate and its circulation is slightly associated with temperature.

In temperate countries, RSV peak activity occurs in the winter and several studies have shown the connection between cold temperatures and viral circulation [44]. In contrast, in tropical countries there is a wide range of variability in the timing and duration of epidemics and the correlation between climatic factors and viral activity is controversial [21,45]. Although in the Southern Hemisphere RSV wave usually starts between March and June and decreases between August and October [21], in Brazil, a continental country with five geographic regions, a wide variation in the seasonality is seen, such as those observed in northeastern [46] and southern [47] regions.

Here we showed that RSV's activity were very similar between 2016 and 2017 seasons, with the circulation onset occurring in March (EW 12) and end in July/August (EW 31-32), during the winter season. These data are in accord with the Brazilian Society of Pediatrics, which recommends the administration of Palivizumab from February to July [48]. Nonetheless, in 2018, we observed an anticipation of the seasonality onset by nine weeks, with the beginning of circulation occurring in January (summer season) and with the end taking place in the Fall instead of Winter.

In the southeastern region it was observed that RSV peak usually happens in early April [49]. Our data shows that in 2016 the RSV peak occurred in May, suggesting subtle differences even inside the same geographical region. In 2018 there was an extension of RSV's seasonality duration by 4.5 weeks when compared to the average in 2016-2017. Those observations are especially worrisome, since major variations could make a preventive measure harder to implement. Understanding local epidemics is important in managing time of prophylaxis, to support vaccine development and to follow morbidity and mortality caused by RSV infection [44], thus, establishing RSV surveillance in real time may allow for the identification of patterns and possible variation in prophylaxis time. RSV seasonality usually lasts five to six months [21]. In our study, the longest seasonal period occurred in 2018 (6 months), followed by 2016 (5 months) and 2017 (4.75 months). Interestingly, the prevalence of RSV-A was high in 2018 (96%), medium in 2016 (41%) and low in 2017 (18%). These data reinforce the theory that RSV-A may lengthen the seasonality [50].

Climatic factors, such as humidity, rainfall and temperature have been assumed to impact RSV seasonality [44,51]. However, this association remains controversial. An inverted correlation between RSV circulation, temperature and humidity was observed in a Brazilian study, carried out in São Paulo State [52]. In this study, a minor correlation was found between temperature decrease and case number increase. However, no correlation was found concerning humidity or precipitation.

ON1 and BA were the only genotypes detected.

All RSV-A isolates were ON1 genotype and all RSV-B were BA, which confirm the fast-global dissemination of RSV with nucleotide duplication. These findings are consistent

with recent published reports performed in other countries, such as Philippines [53], Kenya [54], Italy [55], USA and Puerto Rico [56].

Overall p-distance during the study period in RSV-A was 1.8%. A recent study observed an overall p-distance of 1.4% within ON1 [13]. A noteworthy observation is the fact that in 2017 we found the lowest prevalence of RSV-A in ES (18%), and, still, the highest genetic diversity. Phylogeny showed that 2017 strains were distributed in almost all genetic clusters, which showed high diversity that year. RSV-A phylogenetic analysis revealed ongoing genetic changes, with BR.1 grouping most recent strains, suggesting that BR.1 strains may be under positive selective pressure. Changes in the circulation of RSV strains have been considered a mechanism for evading immune response generated by previous strains, which possibly allows for re-infections to occur [57].

As demonstrated, in 2018 RSV-B was responsible for only 4% of cases. Therefore, phylogenetic analysis did not include any RSV-B samples from that year. Older strains, from 2009 to 2014, are positioned at the base of the BA cluster, however, sample strains collected between 2015 and 2018 did not form genetic groups related to the year of collection. This observation may suggest an absence of positive pressure.

Although we found clusters composed exclusively of ES samples, it is necessary to expand the sequencing of RSV samples globally in order to verify if there is in fact the formation of local genetic groups or if the observation is caused by a sample bias.

Previous studies show that a large part of the genetic variability between RSV strains comes from changes in O-glycosylation profile and that this may be associated with an evolutionary mechanism of immune response evasion [59]. Here, we investigated and listed strain amino acid substitutions and also those shared within and between clusters. However, we did not carry out in-depth analysis in order to understand the role of these mutations, therefore, our objective was purely observational. Among the

mutations found, one of the most interesting was the insertion of tree nucleotides at codon 228 in RSV-B. Further studies are essential to understand virus evolution and pathogenicity mutation consequences.

Limitations of this study include the fact that the majority of patients had an acute infection, thus, the prevalence found refers only to SARI, and the absence of a mild infection group prevents further analysis of severity influencing factors. Lastly, clinical data were taken from notification forms, which often show inconsistencies and missing data. Despite those caveats, we believe the data provide valuable epidemiological, genetic and clinical information on RSV.

Conclusion

In this study we observed a high prevalence of RSV in children under three years old even when using the Brazilian Influenza Surveillance Program. This result is important because it shows that the establishment of global RSV surveillance within the Influenza surveillance system allows for the detection of a large number of cases. Our data suggest that RSV-A is, in fact, more virulent than RSV-B. Notably, no correlation between viral load and disease severity was observed. The observation of an important anticipation of the seasonal period is worrisome, since this can make it difficult to administer prophylactic measures at the right time, however, it is necessary to expand the historical series of seasonality in Espirito Santo. The average temperature was the only climatic factor to show interference with the viral circulation. Our data show the annual co-circulation of RSV-A and RSV-B, however, with considerable fluctuations in the prevalence of subtypes. ON1 and BA were the only ones found in the studied period, which corroborates with a series of recent studies. The establishment of a global and

standardized real time RSV surveillance may allow for the collection of data that will help understanding the complex mechanisms of viral evolution and will facilitate the development of future vaccines and antiviral drugs. RSV continues to lead the cause of hospitalizations for pneumonia in children worldwide, being responsible for a large fraction of morbidity and mortality in the pediatric population.

Acknowledgments

We would like to thank Liliana Cruz Spano for her significant theoretical and experimental support to this work, all researchers who upload genetic sequences in the public genetic database – GenBank, patients, parents and guardians, the Espirito Santo State Health Department and the Brazilian Ministry of Health, represented by the Influenza Technical Group.

References

1. Hall CB, Weinberg GA, Iwane MK, Blumkin AK, Edwards KM, Staat MA, et al. The Burden of Respiratory Syncytial Virus Infection in Young Children. N Engl J Med. 2009;360: 588–598. doi:10.1056/NEJMoa0804877

2. Borchers AT, Chang C, Gershwin ME, Gershwin LJ. Respiratory Syncytial Virus —A Comprehensive Review. Clin Rev Allergy Immunol. 2013;45: 331–379. doi:10.1007/s12016-013-8368-9

3. Collins PL, Graham BS. Viral and Host Factors in Human Respiratory Syncytial Virus Pathogenesis. J Virol. 2008;82: 2040–2055. doi:10.1128/JVI.01625-07

4. El Saleeby CM, Bush AJ, Harrison LM, Aitken JA, DeVincenzo JP. Respiratory Syncytial Virus Load, Viral Dynamics, and Disease Severity in Previously Healthy Naturally Infected Children. J Infect Dis. 2011;204: 996–1002. doi:10.1093/infdis/jir494

5. Hasegawa K, Jartti T, Mansbach JM, Laham FR, Jewell AM, Espinola JA, et al. Respiratory Syncytial Virus Genomic Load and Disease Severity Among Children

Hospitalized With Bronchiolitis: Multicenter Cohort Studies in the United States and Finland. J Infect Dis. 2015;211: 1550–1559. doi:10.1093/infdis/jiu658

6. Resa C, Magro S, Marechal P, Barranger C, Joannes M, Miszczak F, et al. Development of an efficient qRT-PCR assay for quality control and cellular quantification of respiratory samples. J Clin Virol. 2014;60: 270–275. doi:10.1016/j.jcv.2014.03.019

7. Mazur NI, Higgins D, Nunes MC, Melero JA, Langedijk AC, Horsley N, et al. The respiratory syncytial virus vaccine landscape: lessons from the graveyard and promising candidates. Lancet Infect Dis. 2018;18: e295–e311. doi:10.1016/S1473-3099(18)30292-5

8. Mufson MA, Orvell C, Rafnar B, Norrby E. Two Distinct Subtypes of Human Respiratory Syncytial Virus. J Gen Virol. 1985;66: 2111–2124. doi:https://doi.org/10.1099/0022-1317-66-10-2111

9. McConnochie KM, Hall CB, Walsh EE, Roghmann KJ. Variation in severity of respiratory syncytial virus infections with subtype. J Pediatr. 1990;117: 52–62. doi:10.1016/S0022-3476(05)82443-6

10. Walsh EE, McConnochie KM, Long CE, Hall CB. Severity of Respiratory Syncytial Virus Infection Is Related to Virus Strain. J Infect Dis. 1997;175: 814–820. doi:10.1086/513976

11. Hornsleth A, Klug B, Nir M, Johansen J, Hansen K, Christensen L, et al. Severity of respiratory syncytial virus disease related to type and genotype of virus and to cytokine values in nasopharyngeal secretions. Pediatr Infect Dis J. 1998;17: 1114–1121. doi:10.1097/00006454-199812000-00003

12. Kim Y-I, Murphy R, Majumdar S, Harrison LG, Aitken J, DeVincenzo JP. Relating plaque morphology to respiratory syncytial virus subgroup, viral load, and disease severity in children. Pediatr Res. 2015;78: 380–388. doi:10.1038/pr.2015.122

13. Muñoz-Escalante JC, Comas-García A, Bernal-Silva S, Robles-Espinoza CD, Gómez-Leal G, Noyola DE. Respiratory syncytial virus A genotype classification based on systematic intergenotypic and intragenotypic sequence analysis. Sci Rep. 2019;9: 20097. doi:10.1038/s41598-019-56552-2

14. Trento A, Viegas M, Galiano M, Videla C, Carballal G, Mistchenko AS, et al. Natural History of Human Respiratory Syncytial Virus Inferred from Phylogenetic Analysis of the Attachment (G) Glycoprotein with a 60-Nucleotide Duplication. J Virol. 2006;80: 975–984. doi:10.1128/JVI.80.2.975-984.2006

15. Eshaghi A, Duvvuri VR, Lai R, Nadarajah JT, Li A, Patel SN, et al. Genetic Variability of Human Respiratory Syncytial Virus A Strains Circulating in Ontario: A Novel Genotype with a 72 Nucleotide G Gene Duplication. PLoS ONE. 2012;7: e32807. doi:10.1371/journal.pone.0032807

16. WHO strategy to pilot global respiratory syncytial virus surveillance based on the Global Influenza Surveillance and Response System (GISRS). Geneva: World Health Organization; 2017. Licence: CC BY-NC-SA 3.0 IGO.

17. Brasil. Panorama do Espírito Santo [cited 27 September 2020]. In Instituto Brasileiro de Geografia e Estatística (IBGE) [Internet]. Available from: https://cidades.ibge.gov.br/brasil/es/panorama.

18. Brasil. Gripe (influenza): causas, sintomas, tratamento, diagnóstico e prevenção. [cited 08 September 2020]. In: Brazilian Ministry of Health [Internet]. Available from: https://antigo.saude.gov.br/saude-de-a-z/gripe/#boletins.

19. Martinello RA, Chen MD, Weibel C, Kahn JS. Correlation between Respiratory Syncytial Virus Genotype and Severity of Illness. J Infect Dis. 2002;186: 839–842. doi:10.1086/342414

20. Álvarez-Argüelles ME, Oña-Navarro M de, Rojo-Alba S, Torrens-Muns M, Junquera-Llaneza ML, Antonio-Boga J, et al. Quantification of human papilloma virus (HPV) DNA using the Cobas 4800 system in women with and without pathological alterations attributable to the virus. J Virol Methods. 2015;222: 95–102. doi:10.1016/j.jviromet.2015.05.016

21. Obando-Pacheco P, Justicia-Grande AJ, Rivero-Calle I, Rodríguez-Tenreiro C, Sly P, Ramilo O, et al. Respiratory Syncytial Virus Seasonality: A Global Overview. 2018; 217(9): 1356-1364. doi:10.1093/infdis/jiy056.

22. Sequencher® - DNA sequence analysis software. Ann Arbor, MI USA: Gene Codes Corporation;

23. Tamura K, Stecher G, Peterson D, Filipski A, Kumar S. MEGA6: Molecular Evolutionary Genetics Analysis Version 6.0. Mol Biol Evol. 2013;30: 2725–2729. doi:10.1093/molbev/mst197

24. Guindon S, Gascuel O. A Simple, Fast, and Accurate Algorithm to Estimate Large Phylogenies by Maximum Likelihood. Syst Biol. 2003;52: 696–704. doi:10.1080/10635150390235520

25. Gardinassi L, Simas P, Gomes D, Bonfim C, Nogueira F, Garcia G, et al. Diversity and Adaptation of Human Respiratory Syncytial Virus Genotypes Circulating in Two Distinct Communities: Public Hospital and Day Care Center. Viruses. 2012;4: 2432–2447. doi:10.3390/v4112432

26. Vieira SE, Thomazelli LM, de Paulis M, Ferronato AE, Oliveira DB, Martinez MB, et al. Infections Caused by HRSV A ON1 Are Predominant among Hospitalized Infants with Bronchiolitis in São Paulo City. BioMed Res Int. 2017;2017: 1–7. doi:10.1155/2017/3459785

27. Checon RE, Siqueira MM, Lugon AK, Portes S, Dietze R. Short report: seasonal pattern of respiratory syncytial virus in a region with a tropical climate in southeastern Brazil. Am J Trop Med Hyg. 2002;67: 490–491. doi:10.4269/ajtmh.2002.67.490

28. Bradley JP, Bacharier LB, Bonfiglio J, Schechtman KB, Strunk R, Storch G, et al. Severity of Respiratory Syncytial Virus Bronchiolitis Is Affected by Cigarette Smoke Exposure and Atopy. Pediatrics. 2005;115: e7–e14. doi:10.1542/peds.2004-0059

29. Cardena MMSG, Ribeiro-dos-Santos Â, Santos S, Mansur AJ, Pereira AC, Fridman C. Assessment of the Relationship between Self-Declared Ethnicity, Mitochondrial Haplogroups and Genomic Ancestry in Brazilian Individuals. PLoS ONE. 2013;8: e62005. doi:10.1371/journal.pone.0062005

30. Brasil. Síntese de Indicadores Sociais: uma análise das condições de vida da população brasileira [cited 27 September 2020]. In Instituto Brasileiro de Geografia e Estatística (IBGE) [Internet]. Available from: https://biblioteca.ibge.gov.br/visualizacao/livros/liv101629.pdf.

31. Nair H, Nokes DJ, Gessner BD, Dherani M, Madhi SA, Singleton RJ, et al. Global burden of acute lower respiratory infections due to respiratory syncytial virus in young children: a systematic review and meta-analysis. Lancet. 2010;375: 1545–1555. doi:10.1016/S0140-6736(10)60206-1

32. Espinosa Y, San Martín C, Torres A, Farfán M, Torres J, Avadhanula V, et al. Genomic Loads and Genotypes of Respiratory Syncytial Virus: Viral Factors during Lower Respiratory Tract Infection in Chilean Hospitalized Infants. Int J Mol Sci. 2017;18: 654. doi:10.3390/ijms18030654

33. Rodriguez-Fernandez R, Tapia LI, Yang C-F, Torres JP, Chavez-Bueno S, Garcia C, et al. Respiratory Syncytial Virus Genotypes, Host Immune Profiles, and Disease Severity in Young Children Hospitalized With Bronchiolitis. J Infect Dis. 2018;217: 24–34. doi:10.1093/infdis/jix543

34. Tran DN, Pham TMH, Ha MT, Tran TTL, Dang TKH, Yoshida L-M, et al. Molecular Epidemiology and Disease Severity of Human Respiratory Syncytial Virus in Vietnam. PLoS ONE. 2013;8: e45436. doi:10.1371/journal.pone.0045436

35. Hall CB, Walsh EE, Schnabel KC, Long CE, McConnochie KM, Hildreth SW, et al. Occurrence of Groups A and B of Respiratory Syncytial Virus over 15 Years: Associated Epidemiologic and Clinical Characteristics in Hospitalized and Ambulatory Children. J Infect Dis. 1990;162: 1283–1290. doi:10.1093/infdis/162.6.1283

36. Jafri HS, Wu X, Makari D, Henrickson KJ. Distribution of Respiratory Syncytial Virus Subtypes A and B Among Infants Presenting to the Emergency Department With Lower Respiratory Tract Infection or Apnea. Pediatr Infect Dis J. 2013;32: 335–340. doi:10.1097/INF.0b013e318282603a

37. DeVincenzo JP, Wilkinson T, Vaishnaw A, Cehelsky J, Meyers R, Nochur S, et al. Viral Load Drives Disease in Humans Experimentally Infected with Respiratory Syncytial Virus. Am J Respir Crit Care Med. 2010;182: 1305–1314. doi:10.1164/rccm.201002-0221OC

38. Gómez- Novo M, Boga JA, Álvarez- Argüelles ME, Rojo- Alba S, Fernández A, Menéndez MJ, et al. Human respiratory syncytial virus load normalized by cell

quantification as predictor of acute respiratory tract infection. J Med Virol. 2018;90: 861–866. doi:10.1002/jmv.25020

39. Hijano DR, Brazelton de Cardenas J, Maron G, Garner CD, Ferrolino JA, Dallas RH, et al. Clinical correlation of influenza and respiratory syncytial virus load measured by digital PCR. PLoS ONE. 2019;14: e0220908. doi:10.1371/journal.pone.0220908

40. Moreira FB, Rosario CS, Santos JS, Avanzi VM, Nogueira MB, Vidal LR, et al. Molecular characterization and clinical epidemiology of human respiratory syncytial virus (HRSV) A and B in hospitalized children, Southern Brazil. J Med Virol. 2017;89: 1489–1493. doi:10.1002/jmv.24795

41. Fodha I, Vabret A, Ghedira L, Seboui H, Chouchane S, Dewar J, et al. Respiratory syncytial virus infections in hospitalized infants: Association between viral load, virus subgroup, and disease severity. J Med Virol. 2007;79: 1951–1958. doi:10.1002/jmv.21026

42. Garcia-Mauriño C, Moore-Clingenpeel M, Thomas J, Mertz S, Cohen DM, Ramilo O, et al. Viral Load Dynamics and Clinical Disease Severity in Infants With Respiratory Syncytial Virus Infection. J Infect Dis. 2019;219: 1207–1215. doi:10.1093/infdis/jiy655

43. Piedra F-A, Mei M, Avadhanula V, Mehta R, Aideyan L, Garofalo RP, et al. The interdependencies of viral load, the innate immune response, and clinical outcome in children presenting to the emergency department with respiratory syncytial virus-associated bronchiolitis. PLoS ONE. 2017;12: e0172953. doi:10.1371/journal.pone.0172953

44. Haynes AK, Manangan AP, Iwane MK, Sturm-Ramirez K, Homaira N, Brooks WA, et al. Respiratory Syncytial Virus Circulation in Seven Countries With Global Disease Detection Regional Centers. J Infect Dis. 2013;208: S246–S254. doi:10.1093/infdis/jit515

45. Bloom-Feshbach K, Alonso WJ, Charu V, Tamerius J, Simonsen L, Miller MA, et al. Latitudinal Variations in Seasonal Activity of Influenza and Respiratory Syncytial Virus (RSV): A Global Comparative Review. PLoS ONE. 2013;8: e54445. doi:10.1371/journal.pone.0054445

46. Moura FEA, Borges LC, Portes SAR, Ramos EAG, Siqueira MM. Respiratory syncytial virus infections during an epidemic period in Salvador, Brazil: viral antigenic group analysis and description of clinical and epidemiological aspects. Mem Inst Oswaldo Cruz. 2003;98: 739–743. doi:10.1590/S0074-02762003000600005

47. Straliotto SM, Siqueira MM, Muller RL, Fischer GB, Cunha MLT, Nestor SM. Viral etiology of acute respiratory infections among children in Porto Alegre, RS, Brazil. Rev Soc Bras Med Trop. 2002;35: 283–291. doi:10.1590/S0037-86822002000400002

48. Kfouri RA, Sadeck LSR, Moura AA, Bresolin AC, Miralha AL, Pimentel AM, et al. Diretrizes para o manejo da infecção causada pelo Vírus Sincicial Respiratório (VSR). Soc Bras Pediatr. 2017; 1–20.

49. Freitas ARR, Donalisio MR. Respiratory syncytial virus seasonality in Brazil: implications for the immunisation policy for at-risk populations. Mem Inst Oswaldo Cruz. 2016;111: 294–301. doi:10.1590/0074-02760150341

50. Yu J, Liu C, Xiao Y, Xiang Z, Zhou H, Chen L, et al. Respiratory Syncytial Virus Seasonality, Beijing, China, 2007–2015. Emerg Infect Dis. 2019;25: 1127–1135. doi:10.3201/eid2506.180532

51. Zhang H, Wen S, Zheng J, Chen X, Lv F, Liu L. Meteorological factors affecting respiratory syncytial virus infection: A time- series analysis. Pediatr Pulmonol. 2020;55: 713–718. doi:10.1002/ppul.24629

52. Gardinassi LG, Simas PVM, Salomão JB, Durigon EL, Trevisan DMZ, Cordeiro JA, et al. Seasonality of viral respiratory infections in Southeast of Brazil: the influence of temperature and air humidity. Braz J Microbiol. 2012;43: 98–108. doi:10.1590/S1517-83822012000100011

53. Okamoto M, Dapat CP, Sandagon AMD, Batangan-Nacion LP, Lirio IC, Tamaki R, et al. Molecular Characterization of Respiratory Syncytial Virus in Children With Repeated Infections With Subgroup B in the Philippines. J Infect Dis. 2018;218: 1045–1053. doi:10.1093/infdis/jiy256

54. Otieno JR, Kamau EM, Oketch JW, Ngoi JM, Gichuki AM, Binter Š, et al. Whole genome analysis of local Kenyan and global sequences unravels the epidemiological and molecular evolutionary dynamics of RSV genotype ON1 strains. Virus Evol. 2018;4. doi:10.1093/ve/vey027

55. Esposito S, Piralla A, Zampiero A, Bianchini S, Di Pietro G, Scala A, et al. Characteristics and Their Clinical Relevance of Respiratory Syncytial Virus Types and Genotypes Circulating in Northern Italy in Five Consecutive Winter Seasons. PLoS ONE. 2015;10: e0129369. doi:10.1371/journal.pone.0129369

56. Bin Lu, Liu H, Tabor DE, Tovchigrechko A, Qi Y, Ruzin A, et al. Emergence of new antigenic epitopes in the glycoproteins of human respiratory syncytial virus collected from a US surveillance study, 2015–17. Sci Rep. 2019;9: 3898. doi:10.1038/s41598-019-40387-y

57. Hall CB. Respiratory Syncytial Virus and Parainfluenza Virus. N Engl J Med. 2001;344: 1917–1928. doi:10.1056/NEJM200106213442507

58. Elawar F, Griffiths CD, Zhu D, Bilawchuk LM, Jensen LD, Forss L, et al. A Virological and Phylogenetic Analysis of the Emergence of New Clades of Respiratory Syncytial Virus. Sci Rep. 2017;7: 12232. doi:10.1038/s41598-017-12001-6

59. Leemans A, Boeren M, Van der Gucht W, Martinet W, Caljon G, Maes L, et al. Characterization of the role of N-glycosylation sites in the respiratory syncytial

virus fusion protein in virus replication, syncytium formation and antigenicity. Virus Res. 2019;266: 58–68. doi:10.1016/j.virusres.2019.04.006

Supporting information

S1 Fig. RSV-A phylogenetic tree based on 336 bp of the HVR-2 of G gene. The tree was built using maximum likelihood method on MEGA 6.0 software from a MUSCLE alignment, with some manual editions. Reference sequences from each described genotype were downloaded from NCBI GenBank and used in the phylogenetic reconstruction. The genotypes were classified by colors and all ES strains grouped within the ON1 genotype.

S2 Fig. RSV-A phylogenetic tree based on 318 bp of the HVR-2 of G gene. The tree was built using maximum likelihood method on MEGA 6.0 software from a MUSCLE alignment, with some manual editions. Reference sequences from each described genotype were downloaded from NCBI GenBank and used in the phylogenetic reconstruction. The genotypes were classified by colors and all ES strains grouped within the BA genotype.

S1 Table. Primers, probes and DNA fragments used in the study. “F”, “R” and “P”, represent the sequence of the forward and reverse primers and the probe, respectively. Synthetic DNA fragment from RSV was included in a pMA-t vector.

S2 Table. List of the sequences used to build the phylogeny based on HVR-2 of gene G for both subtypes RSA-A and RSV-B.

S3 Table. List of the sequences used to build the phylogeny based on gene G for both subtypes RSV-A and RSV-B. Collection date of some sequences were unavailable.

S4 Table. Differences in severity among ethnicities showing that children classified as black or brown showed O_2 saturation $\leq$ 95% and respiratory distress more often than those classified as white. Also they required ventilatory support more frequently and stand more time in hospital and in ICU.

S5 Table. Duration and climatic characteristics of RSV seasonality in the years studied.

S6 Table. List of amino acid changes in RSV-A. Residues in blue and red show potential losses and gains of O-glycosylation sites, respectively.

S7 Table. List of amino acid changes in RSV-B. Residues in blue and red show potential losses and gains of O-glycosylation sites, respectively.

Figure 1

Click here to access/download;Figure;Fig1.tif

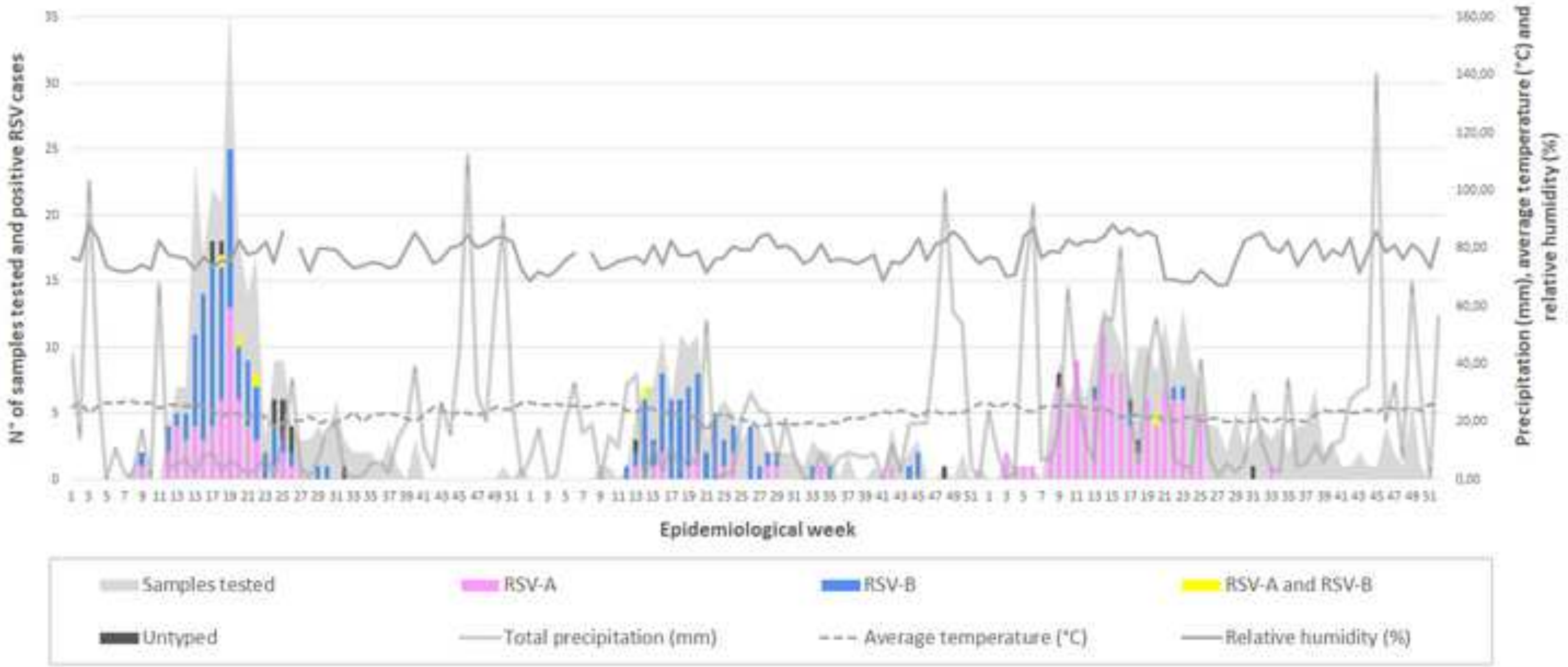

Figure 2

Click here to access/download;Figure;Fig 2.eps

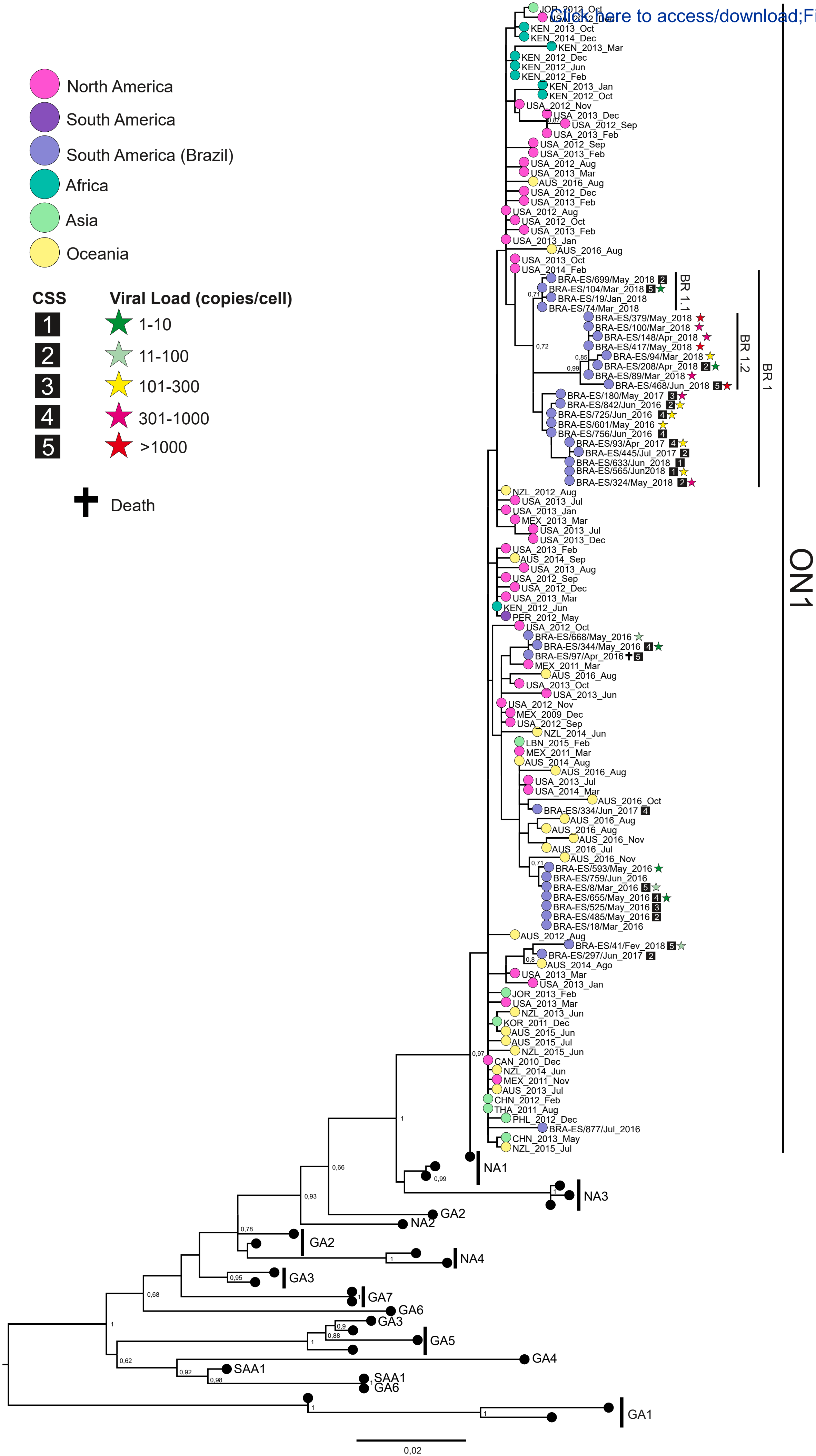

Figure 3

Click here to access/download;Figure;Fig 3.eps

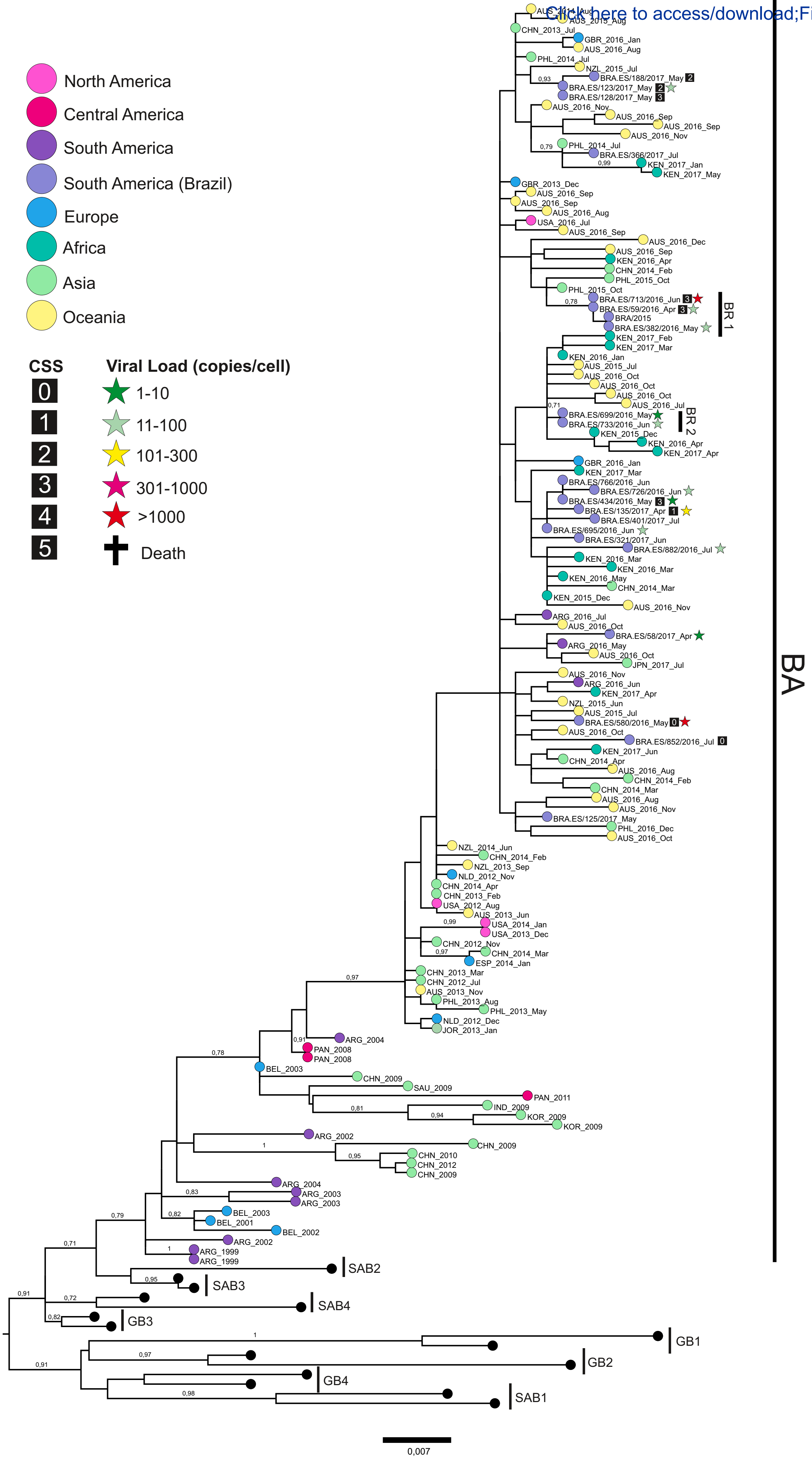

S1 Fig

Click here to access/download
Supporting Information
S1 Fig.tif

S2 Fig

Click here to access/download
Supporting Information
S2 Fig.tif

S1 Table

Click here to access/download
Supporting Information
S1_Table.docx

S2 Table

Click here to access/download
Supporting Information
S2_Table.docx

S3 Table

Click here to access/download
Supporting Information
S3_Table.docx

S4 Table

Click here to access/download
Supporting Information
S4_Table.docx

S5 Table

Click here to access/download
Supporting Information
S5_Table.docx

S6 Table

Click here to access/download
Supporting Information
S6_Table.xlsx

S7 Table

Click here to access/download
Supporting Information
S7_Table.xlsx

Full Title: Seasonality, molecular epidemiology and virulence~~Landscape~~ of Respiratory Syncytial Virus (RSV): a perspective into the Brazilian Influenza Surveillance Program

Short Title: Landscape of Respiratory Syncytial Virus (RSV)

Lucas A. Vianna[1,2], Marilda M. Siqueira[3], Lays P. B. Volpini[4], Iuri D. Louro[2], Paola C. Resende[3]

[1] Central Laboratory of Public Health of the State of Espirito Santo, Vitoria, Espirito Santo, Brazil.

[2] Nucleus of Human and Molecular Genetics/ Federal University of Espirito Santo/ UFES, Vitoria, Espirito Santo, Brazil.

[3] Laboratory of Respiratory Viruses and Measles, National Influenza Center (NIC)/ World Health Organization (WHO), Oswaldo Cruz Institute, Oswaldo Cruz Foundation, Rio de Janeiro, Rio de Janeiro, Brazil.

[4] Virology & Infectious Gastroenteritis Laboratory / Federal University of Espirito Santo /UFES, Vitoria, Espirito Santo, Brazil.

Abstract

Background: Respiratory Syncytial Virus (RSV) is the main cause of pediatric morbidity and mortality~~in children~~. The complex evolution of RSV creates a need for worldwide surveillance, which may assist in the understanding of multiple viral aspects.~~Understanding RSV's seasonality, clinical features, contribution of viral load and genetic diversity in Brazil may contribute to improve the global surveillance and the development of vaccines and antiviral drugs.~~

Objectives: This study aimed to investigate RSV features under the ~~perspective of the~~ Brazilian Influenza Surveillance Program, evaluating the role of viral load and genetic

Formatted: Font: (Default) Times New Roman, Font color: Auto

Formatted: Not Superscript/ Subscript

Formatted: Not Superscript/ Subscript

Formatted: Not Superscript/ Subscript

Formatted: Font: 18 pt

diversity in ~~the~~ disease severity and the influence of climatic factors in ~~the~~ viral seasonality.

Methodology: ~~From 2016 to 2018, w~~We have investigated the prevalence of RSV in children up to 3 years old with severe acute respiratory infection (SARI) in the Espirito Santo State (ES), Brazil, from 2016 to 2018. ~~Testing by~~ RT-qPCR allowed for ~~the~~ viral detection and ~~measure of~~ viral load quantification, ~~in order~~ to evaluate association with clinical features, and~~as well as~~ mapping of local viral seasonality. Gene G sequencing and phylogenetic reconstruction ~~showed the~~demonstrated local genetic diversity.

Results: Of 632 evaluated cases, 56% were caused by RSV, with both subtypes A and B co-circulating throughout the years. A discrete inverse association between average temperature and viral circulation was observed. No correlation between viral load and severity was observed, but children infected with RSV-A presented higher clinical severity score (CSS) median, stayed longer in the hospital, required more ~~often~~ intensive care and ventilatory support than those infected by RSV-B. Regarding ~~the~~ RSV diversity, some local genetic groups were observed in the main genotypes circulation RSV-A ON1 and RSV-B BA~~,~~, with strains showing modifications in the G gene amino acid chain. ~~associated new reported amino acid changes.~~

Conclusion: Local RSV studies using~~of~~ the Brazilian Influenza Surveillance Program are relevant because they can reveal useful information, contributing to the global RSV surveillance. Understanding seasonality, virulence and~~,~~ genetic diversity can ~~guarantee~~support the suitability of future antiviral drugs and vaccines ~~to circulating strains~~ and assist in the ~~most opportune time of the~~ administration of prophylactic strategies.~~can support antiviral and vaccine development.~~

Formatted: Not Highlight

Formatted: English (United States)

Formatted: Highlight

Introduction

Formatted: Font: 18 pt

Respiratory Syncytial Virus (RSV) is the most common pathogen associated with acute respiratory tract infections (ARTI), being the main cause of bronchiolitis and pneumonia in infants and small children [1]. ~~In 2015, RSV was responsible for approximately 33.1 million cases of acute lower respiratory infections (ALRI) in children under 5 years old and more than 59.000 deaths worldwide [2]. In Brazil, according to the Influenza and other respiratory viruses epidemiological reports, RSV has shown an important role on severe acute respiratory infections (SARI), ranging from 9.75 to 17.3% between 2016 and 2019 [3]. In 2019, 5% of the deaths due to SARI were caused by RSV [3].~~

RSV infection can cause a ~~diverse~~ range of symptoms, varying from ~~a~~ mild upper respiratory tract illness to~~ a~~ severe lower respiratory tract infection [2]. The reason for different outcomes is still unclear, however it can be related to the underline conditions, genetic or acquired host factors, and/or viral characteristics [3,4]. Some studies have evaluated the association between viral load and disease severity, with significant associations [4,5]. However, most of these studies did not use standardized methods of ~~measuring~~ viral load measurement, therefore~~us~~, this relationship must be more carefully evaluated. The understanding of RSV infection viral load may be a tool to establish its relationship with disease progression, severity, clinical outcome and~~or the moment to~~ drug intervention timeframe [6].

~~The~~ RSV treatment is based only in supportive care and infection prevention is limited to passive immunoprophylaxis (Palivizumab) and case isolation [2]. No licensed RSV vaccine is available, but some promising candidates are currently in development and in advanced clinical trial phases [7].

~~Based on the reactivity of monoclonal antibodies,~~ RSV strains can be classified into two serogroups: RSV-A and RSV-B [8]. The potential virulence attributed to a

specific group remains controversial: some authors have pointed RSV-A [9,10] or RSV-B [11] as the most virulent subtype, other studies have not found~~did not find~~ significant differences between them [12].

~~As a typical RNA virus, RSV evolution is complex and dynamic, presenting remarkable changes over the time, with the emergence of new genotypes and extinction of others [13].~~ Multiple genotypes were described for RSV-A and RSV-B, based on ~~the~~ gene G second hypervariable region (HVR-2) ~~of gene G~~ [13,14]. In the past two decades important shifts occurred with the emergence of new RSV-A and RSV-B genotypes: RSV-A~~,~~ ON1 containing a duplication of 72 nucleotides, and RSV-B BA with a duplication of 60 nucleotides in the HVR-2 gene G [14,15]. These genotypes replaced ~~the~~ previous ones and have spread globally. Understanding their genetic diversity may reveal the virus's ability to cause re-infections throughout life, and help designing antiviral drugs, diagnostic assays and vaccines [13].

In 2017, the World Health Organization (WHO) launched the Global Respiratory Syncytial Virus Surveillance Pilot in order to test the feasibility of using the Global Influenza Surveillance and Response System (GISRS) for RSV surveillance without adversely affecting influenza surveillance [16]. This pilot study results from the global concern about RSV impact on public health. Brazil, one of ~~the~~ four countries in the Americas ~~to be~~ included in the pilot, has a remarkable respiratory virus surveillance program, however, more data are required for a better understanding of factors such as RSV circulation, evolution, and pathogenicity. ~~In the light of this perspective, w~~ In this study, we used the Brazilian Influenza Surveillance Program to analyze the local prevalence of RSV in children with SARI and to evaluate which factors are potentially associated with disease severity. We also explored the viral seasonality and investigate the influence of climatic factors in the circulation. Finally, we conducted a phylogenetic

Formatted: English (United States)

Formatted: English (United States)

Formatted: English (United States)

study to understand how the local genetic diversity of RSV behaves with that observed in the rest of the world.We have performed an RSV molecular and epidemiological study from 2016 to 2018 in the Espirito Santo State (ES), southeastern Brazil. Sampling was obtained from the National INFLUENZA SURVEILLANCE PROGRAM of children diagnosed with SARI. Here we highlight the high prevalence of RSV in children with SARI and its possible association with severe disease. Additionally, we have shown the seasonality oscillation in the ES state, creating difficulties for the determination of the most appropriate period to start prophylaxis and control actions to mitigate viral circulation.

Formatted: Highlight

Materials and methods

Formatted: Font: 18 pt

Ethics Statement. This project was approved by the Human Research Ethics Committee of the Health Sciences Center of the Federal University of Espirito Santo (UFES), under the number: 018577/2018; CAAE: 84633518.1.0000.5060.

Population sampling, study period and location Sampling.

Formatted: Font: 16 pt

This study is a retrospective investigation ofn respiratory samples (nasopharyngeal secretions, tracheal and bronchoalveolar aspirate and bronchoalveolar lavage) collected from the Brazilian Influenza Surveillance Program during 34 months (March 7th, 2016, to December 14th, 2018). A total of Up to 632 samples collected from pediatric patients (from 0 to 36 months old) classified as SARI, residents of 60 municipalities in the ES State, were enrolled in this study. ES state is located in southeastern Brazil and it has a territory of 46.074,447 km^2, with a population of approximately 4.1 million inhabitants [17]. These samples were screened by real-time RT-qPCR for RSV and Influenza A/B at the ES Central Public Health Laboratory of Public Health of Espirito Santo (LACEN/ES),

one of 26 Brazilian laboratories that integrate the ~~National~~ Brazilian Ministry of Health Influenza Surveillance Program~~ of the Brazilian Ministry of Health~~.

RSV and Influenza detection and subtyping

Formatted: Font: 16 pt

~~The~~ ~~n~~Nucleic acids were extracted from ~~the~~ respiratory samples using the PureLink™ Viral RNA/DNA Mini Kit (Invitrogen®, Thermo Fisher Scientific©), according to ~~the~~ manufacturer's protocol. All samples were tested initially for~~by~~ Influenza A and B in a TaqMan® one-step real time RT-PCR (RT-qPCR) assay with primers and probes specific for influenza (CDC, USA), according to manufacturer's recommendations. Additionally, RT-qPCR assay was performed to identify positive RSV cases ~~of RSV~~ using GoTaq® Probe 1-Step RT-qPCR Kit (Promega, Madison, WI, EUA). RSV positive samples (*i.e.* those with cycle threshold [CT] ≤ 40) were subtyped using specific primers and probes to RSV-A and RSV-B N gene~~of RSV-A and RSV-B~~. In parallel, Ribonuclease P RNA (RNase P) was ~~also~~ tested as an internal control for each sample and all batches ~~tested~~ had an RNA extraction negative control (MOCK) and a PCR negative control (NTC). All primers and probes are described ~~description are listed~~ in the **S1 Table**.

Clinical and epidemiological data collection~~.~~

Formatted: Font: 16 pt

Clinical and epidemiological data were retrieved mainly from the Information System of~~n~~ Diseases of Compulsory Declaration of Notification Illness (SINAN) and, in some cases – where the SINAN file was incomplete – we assessed ~~the~~ patients' Medical Records ~~from the patients~~ to fill missing information. The main information recovered from SINAN were: 1) clinical outcome (recovered or death); 2) ~~length of~~ hospitalization length ~~stay~~; 3) ~~necessity and type of~~ oxygen administration need and type (invasive or not invasive), 4) ~~necessity and length of~~ intensive care unit (ICU) need and length; 5) clinical

characteristics (fever, cough, dyspnea, O_2 saturation, respiratory distress, comorbidities) and 6) epidemiological and demographical features (age, race, town or area of residence). Here wWe have used the Brazilian Ministry of Health definition of SARI, that is: hospitalized patient with fever and cough or sore throat and presenting dyspnea or O2 saturation <95%, or respiratory distress [18]. A Clinical Severity Score (CSS) was adapted from Martinello *et al*. [19]. The scale ranged from 0 to 5 points, where 0 wasis the mildest condition and 5 is the most severe case. ICU admission, hospitalization length ≥5 days, oxygen saturation ≤95% and oxygen therapy noninvasive methods of oxygen therapy accounted for 1 point each. Two points wereas assigned for if patient required mechanical ventilation.

Viral load measurementquantification.

RSV viral load was determined by RT-qPCR using a protocol adapted from Álvarez-Argüelles *et al*. [21], including a synthetic *β-globin* dsDNA as a template. To quantify the RSV copy number, expressed in copies per cell (c/c), we designed a dsDNA containing the annealing regions of RSV primers and probe, as well as the upstream and downstream regions (, up to a total of 150 bp). This synthetic DNA was incorporated into a pMA-T plasmid, which was used in the RT-qPCR. Standard curves for absolute quantification of RSV and *β-globin* gene were generated by 10-fold serial dilutions (10^6-10^1 copies of genome), in triplicate. RSV primers, probe and the thermal cycling protocol used were the same used in the diagnostic phase. *β-globin* primers and probe are listed in **S1upplementary Table 1**. All amplification assays were carried out in the ABI 7500 equipment (Applied Biosystems, Foster City, CA, USA). Viral load status was compared with different clinical features and epidemiological data.

Formatted: Font: 16 pt

Climate data collection~~.~~

Formatted: Font: 16 pt

Climate data (precipitation, temperature and humidity) of five cities – representatives from the different geographic regions of the state – were daily collected and kindly provided by the Capixaba Institute of Research, Technical Assistance and Rural Extension (INCAPER), Vitoria, Espirito Santo, Brazil. The weekly average was accessed by assembling ~~the~~ daily data from all collection sites for each epidemiological week (EW). The definition of RSV epidemic period was based on a previously described protocol [20], which considers RSV outbreak onset, peak and end. Seasonality onset ~~and end were~~was defined as the first ~~and last~~ of 2 consecutive weeks, ~~respectively,~~ when ≥10% ~~or higher~~ of ~~the total~~ tested samples for respiratory pathogens were positive for RSV~~the number of RSV cases exceeded 10% of the number detected during the RSV peak week~~. Similarly, ~~the end of the~~ RSV season~~ality~~ end was defined when the proportion of positive RSV tests fell below 10% for two consecutive weeks. ~~The p~~Peak was determined as the week when the maximum number of RSV positive cases occurred [20].

Formatted: English (United States)

~~**RSV detection and subtyping**. The nucleic acids were extracted from the respiratory samples using the PureLink™ Viral RNA/DNA Mini Kit (Invitrogen®, Thermo Fisher Scientific©), according to the manufacturer's protocol. All samples were tested initially by Influenza A and B in a TaqMan® one-step real-time RT-PCR (RT-qPCR) assay with primers and probes specific for influenza (CDC, USA), according to manufacturer's recommendations. Additionally, RT-qPCR assay was performed to identify positive cases of RSV using GoTaq® Probe 1-Step RT-qPCR Kit (Promega, Madison, WI, EUA). RSV positive samples (*i.e.* those with cycle threshold [CT] ≤ 40) were subtyped using specific primers and probes to RSV-A and RSV-B. In parallel, Ribonuclease P RNA (RNase P) was also tested as an internal control for each sample and all batches tested had an RNA~~

extraction negative control (MOCK) and a PCR negative control (NTC). All primers and probes description are listed in the **Supplementary Table 1**.

Viral load measurement. RSV viral load was determined by RT-qPCR using a protocol adapted from Álvarez-Argüelles *et al*. [21], including a synthetic *β-globin* dsDNA as a template. To quantify the RSV copy number, expressed in copies per cell (c/c), we designed a dsDNA containing the annealing regions of RSV primers and probe, as well as the upstream and downstream regions, up to a total of 150 bp. This synthetic DNA was incorporated into a pMA-T plasmid, which was used in the RT-qPCR. Standard curves for absolute quantification of RSV and *β-globin* gene were generated by 10-fold serial dilutions (10^6-10^1 copies of genome), in triplicate. RSV primers, probe and the thermal cycling protocol used were the same used in the diagnostic phase. *β-globin* primers and probe are listed in **Supplementary Table 1**. All amplification assays were carried out in the ABI 7500 equipment (Applied Biosystems, Foster City, CA, USA). Viral load status was compared with different clinical features and epidemiological data.

Partial amplification and sequencing of glycoprotein gene

Formatted: Font: 16 pt

. RSV-A and RSV-B positive samples were selected to be sequenced based on the following criteria: a) cycle threshold (ct) value less than 30, due to the difficulty in sequencing samples with ct higher than this; b) representativeness by collection date; c) distinct clinical outcomes; and d) different viral load values.

The partial gene G amplification (about 730 bp) was performed at LVRS/IOC/FIOCRUZ, the National Influenza Center, by conventional RT-PCR, using the QIAGEN OneStep RT-PCR Kit (Qiagen) and a pair of primers (**S1upplementary Table** 1) for each subtype. The reverse transcription was performed at 55°C for 30 minutes and the cDNA was amplified by PCR (40 cycles of 94°C/30 seconds, 60°C /1

minute, 72°C/1 minute and a final extension at 72°C/10 minutes). Amplification was confirmed in a 1% agarose gel. DNA was purified using ExoSap-IT Kit (Affymetrix, Inc., USA) and submitted to sequence reaction using BigDye™ Terminator v3.1 Cycle Sequencing Kit (Applied Biosystems, Foster City, CA, USA) and primers at 3.2 µmolar. The reads were obtained in the ABI 3130XL Genetic Analyzer (Applied Biosystems). Consensus sequences were built from electropherograms comparison with a reference sequence in the software Sequencher 5.1 [22]. The adopted nomenclature pattern here~~i~~on was "hRSV subtype/country/ES-sample number/year."

RSV genotyping and gene G phylogenetic reconstruction

~~.~~RSV-A and RSV-B gene G DNA sequences (~~of~~ 711 bp and 726 bp, respectively)~~,~~ were used to reconstruct phylogenetic relationships. Genotyping was based on gene G HVR-2, using RSV-A and RSV-B sequences (~~of~~ 336 bp and 318 bp ~~in length~~, respectively). Reference sequences of previously described genotypes are shown in **S2~~upplementary~~ Table~~ 2~~**. Additionally, to place our sequences in a global context we performed a BLAST search (Basic Local Alignment Search Tool), available at https://blast.ncbi.nlm.nih.gov/Blast.cgi. These sequences (**S3~~upplementary~~ Table~~ 3~~**) were labeled with country of origin and collection year, and those with more than 99.5% genetic similarity using CD-HIT tool (http://weizhongli-lab.org/cd-hit/servers.php) were removed from the final dataset. Alignments were conducted using Muscle algorithm, via MEGA 6.0 software [23] and when necessary they were adjusted manually. The phylogenetic trees were constructed using the Maximum Likelihood (ML) method, complete deletion for gap or missing data treatment and 1000 replicates of bootstrap probabilities tools integrated within Mega 6.0. General Time Reversible + Gamma (GTR+G) was the nucleotide substitution model elected for all analysis on JModelTest

Formatted: Font: 16 pt

Field Code Changed

Field Code Changed

software, with an exception for RSV-A, which Tamura-Nei + Gamma (TrN+G) was the substitution model nucleotide indicated for the analysis [24]. Average pairwise distance (p-distance) was calculated in Mega 6.0. Amino acid comparisons with the reference sequences of ON1 (JN257693) and BA (AY333364) were performed using MEGA 6.0 software to map the changes in ~~amino acids in~~ Brazilian samples amino acids.

Statistical treatment

Formatted: Font: 16 pt

Statistical analyses were performed using SPSS 20.0 (SPSS, Inc., Chicago, IL) and R v.3.4.4 software. Chi-square, Fisher exact, Mann–Whitney and Kruskal Wallis were used whenever appropriated. To test the association between climate data and RSV circulation we performed the Spearman correlation test. A p-value of less than 0.05 was considered statistically significant.

Data availability

Formatted: Font: 16 pt

The sequences produced here were deposited on GenBank platform, under the accession number MW026969– MW027004 and MW030961-MW030981, and in GISAID platform, under the accession number EPI_ISL_549271- EPI_ISL_549327.

Ethics Statement

Formatted: Font: 16 pt

This project was approved by the Human Research Ethics Committee of the Health Sciences Center of the Federal University of Espirito Santo (UFES), under the number: 018577/2018; CAAE: 84633518.1.0000.5060. The need for parents or guardians consent was waived by the ethics committee.

Results

RSV clinical and epidemiological data.

A total of Up to 632 respiratory samples collected from children under 3 years-old were tested by RT-qPCR for Influenza A, Influenza B and RSV, being RSV the most prevalent pathogen found in these samples (56%; 352/632) (**Table 1**). From RSV positive cases, 54% (180/352) were RSV-A, 147 (44%) were RSV-B, co-detections with both subtypes were found in 5 cases (1.5%). Twenty samples could not be subtyped. TheInfluenza frequency prevalence of Influenza was only 7.4% (47/632), being and of these 10.6% (5/47) were Influenza B, 74.4% (35/47) were Influenza A H1N1 pdm09 and 14.9% (7/47) were H3N2, 14.9% (7/47) H3N2 and 10.6% (5/47) Influenza B.. Male gender was slightly more affected by RSV (n=182; 52%) and median age was 4 months old (1-11.0 interquartile range; IQR). OfFrom positive cases, , being 66.2% between 0 and 7 months old. 99.7% (351/352) of positive cases were classified as SARI and 14 deaths (4%) were reported. Most children of this study were brown (54%), according to the self--racial classification carriedfilled out by declaration of children's legal guardianresponsible.

Racial classification was carried out by declaration of those responsible for the children, being 54% declared brown, 41% white, and black /yellow represented 4%.

Table 1. Number of tested samples, RSV positivity, subtype prevalence and demographic data from each year and from the whole study period of the study. Statistical tests were performed to verify the statistical significance of the data differences amongbetween the years. Statistically significant values are highlighted in redbold.

	**2016 n (%)**	**2017 n (%)**	**2018 n (%)**	***p-value***	**2016-18 n (%)**
**General data**					
Sample n°	251/632 (0.40)	135/632 (0.21)	246/632 (0.39)	-	632/632 (1)
RSV +	155/251 (0.62)	80/135 (0.59)	117/246 (0.48)	**0.003**	352/632 (0.56)
RSV -	96/251 (0.38)	55/135 (0.41)	129/246 (0.52)		280/632 (0.44)

Formatted: Font: 18 pt
Formatted: Font: 16 pt
Formatted: Indent: First line: 0"
Formatted: Font color: Red
Formatted: Font color: Red
Formatted: Font color: Auto
Formatted: Font: 12 pt
Formatted: Font: 11 pt
Formatted: Indent: First line: 0"
Formatted: English (United States)
Formatted: Font: Bold, Font color: Auto
Formatted: Font: Bold

Flu +	27/632 (0.11)	6/135 (0.04)	14/246 (0.06)	-	47/632 (0.07)
RSV+ deaths	6/251 (0.04)	5/135 (0.06)	3/246 (0.03)	0.42[1]	14/352 (0.04)
Subtyped samples	141/251 (0.91)	78/135 (0.98)	113/246 (0.97)	-	332/352 (0.94)
**Subtypes**					
RSV-A	58/141 (0.41)	14/78 (0.18)	108/113 (0.96)	**<0.001[1]**	180/332 (0.54)
RSV-B	80/141 (0.57)	63/78 (0.81)	4/113 (0.04)		147/332 (0.44)
RSV-A and RSV-B	3/141 (0.02)	1/78 (0.01)	1/113 (0.01)		5/332 (0.02)
**Demographic data (RSV+)**					
Median age (months)	4 (1-12.0)	4 (1-10.5)	3 (1-8.0)	0.793	4 (1-11.0)
**Gender**					
Male	72/155 (0.46)	49/80 (0.61)	61/117 (0.52)	0.098	182/352 (0.52)
Female	83/155 (0.54)	31/80 (0.39)	56/117 (0.48)		170/352 (0.48)
**Race**					
White	61/122 (0.50)	26/66 (0.39)	31/97 (0.32)	**0.039[1]**	118/285 (0.41)
Brown	53/122 (0.43)	38/66 (0.58)	64/97 (0.66)		155/285 (0.54)
Black	7/122 (0.06)	1/66 (0.02)	2/97 (0.02)		10/285 (0.04)
Yellow	1/122 (0.01)	1/66 (0.02)	0		2/285 (0.01)
Undeclared	33	14	19		66

[1] Fisher's exact test.

Table 2 shows patients' clinical features by RSV+ and subtypes.~~The Table 2 shows the RSV affected patients' clinical features of patients affected by RSV and the comparison between subtypes.~~ ~~Regarding the clinical features of patients affected by RSV (**Table 2**), cough was~~ T~~t~~he most frequent symptom reported was cough (93%, 318/341), followed by respiratory distress (88%, 269/307), and fever (86%, 288/336).~~, dyspnea (76%, 251/331) and oxygen saturation ≤ 95% (61%, 169/277). Absence of fever was reported in 14.3% (48/336) of cases;~~ ~~.~~74% (252/342) of children needed oxygen therapy and 38% (95/252) of these required mechanical ventilation. The median hospitalization

Formatted: Font: Bold, Font color: Auto
Formatted: Font: Bold
Formatted: Font: Bold, Font color: Auto
Formatted: Font: Bold
Formatted: Font: Not Bold

time was 8 (6-14 IQR) days. Intensive care was needed for 61% (202/333) of patients and the median number of days in ICU was 6 (3-10 IQR).

Table 2. Summary of clinical and epidemiological data by RSV+ and each subtype.~~Summary of clinical and epidemiological data by subtype: RSV-A and RSV-B.~~ Statistically significant values are highlighted in ~~red~~bold.

	RSV+ 2016-2018	Subtypes		
	**n (%)**	**RSV-A**	**RSV-B**	***p-value***
**Demographic profile**				
Sample number	~~632~~ 352~~(1)~~	180	147	
Deaths RSV+	14/352 (0.04)	3/180 (0.02~~17~~)	8/147 (0.05~~4~~)	0.07[1]
**Age**				
Median age: months (IQR)[2]	4 (1-11)	4 (1-10.0)	4 (1-12.5)	0.78
**Gender**				
Male ~~n~~ (%)	182/352 (0.52)	92/180 (0.51)	78/147 (0.53)	0.725
Female ~~n~~ (%)	170/352 (0.48)	88/180 (0.49)	69/147 (0.47)	
**Clinical profile**				
Fever	288/336 (0.86)	147/174 (0.84)	124/139 (0.89)	0.223
Cough	318/341 (0.93)	162/176 (0.92)	134/142 (0.94)	0.418
Dyspnea	251/331 (0.76)	135/172 (0.78)	97/136 (0.71)	0.148
O_2 saturation ≤ 95%	169/277 (0.61)	101/150 (0.67)	56/109 (0.51)	**0.009**
Respiratory distress	269/307 (0.88)	154/167 (0.92)	96/120 (0.80)	**0.002**
O_2 Therapy	252/342 (0.74)	138/177 (0.78)	98/143 (0.68)	
Invasive	95/252 (0.38)	56/138 (0.41)	33/98 (0.34)	0.092
Noninvasive	157/252~~90~~ (0.62)	82/138 (0.59)	65/98 (0.66)	
Intensive Care	202/333 (0.61)	113/168 (0.67)	78/142 (0.55)	**0.03**
Median hospitalization days	8 (6-14)	9 (6-15)	8 (5-14.0)	0.15
Median days in Intensive Care	6 (3-10)	7 (4-11.0)	6 (3-9)	0.13
~~Viral Load Median~~	-	~~57.41~~	~~27.35~~	~~0.03~~

[1] Fisher's exact test.
[2] IQR: interquartil range.

Formatted: Font: Bold, Font color: Auto
Formatted: Font: Bold, Font color: Auto
Formatted: Font: Bold, Font color: Auto
Formatted: Font: Bold

Table 2 shows the clinical data comparison between subtypes. When compared to RSV-B, Ppatients affected by RSV-A showed a higher frequency of respiratory distress (n=154; 92% vs 80%, p= 0.002), and more often manifested O_2 saturation ≤95% (n=101; 67% vs 51%, p= 0.009) when compared to RSV-B (O_2 saturation ≤95%: n=56; 51% and respiratory distress: n=96; 80%), and higher requirement for intensive care. The need for intensive care was higher in patients with RSV-A (n=113; 67% vs 55%, p= 0.03) than for those with RSV-B (78; 55%). Our data also indicate that patients affected by RSV-A, in median, stayed one day longer in hospital and in intensive care units than those affected by RSV-B, howeveryet, these data were not statistically significant. Lastly, RSV-A viral load showed more than twice the number of virus copies per cell (median = 57.41 copies/cell) thaen RSV-B (median=27.35 copies/cell). RSV-A CSS median was 4 and RSV-B's was 3, and children infected by RSV-A were most frequently were classified in higher severity scores than those infected by RSV-B (**Supplementary Table 34**.). The S4 Table shows the difference in severity by ethnicity.

Table 3. Clinical Severity Score (CSS): the score varied between 0 and 5. Higher values were assumed to represented more severe illness. Need for ICU, O2≤95%, length in hospital>5 days and requirement of O2 therapy accounted for 1 point each. Need for mechanical ventilation accounted for 2 points. Patients infected with RSV-A were most commonly classified into the most severe scores. The dDifference between viral loads was not related to severity, but there was no statistical significance.

Clinical Severity Score (CSS)

CSS	RSV-A n (%)	RSV-B n (%)	*p-value*	Viral load median (IQR)	n	*p-value*
0	1 (1%)	10 (15%)	0,003	54.06 (6.12-603.61)	8	0.089
1	8 (8%)	8 (12%)		217.41 (96.38-370.56)	9	
2	19 (20%)	11 (17%)		41.18 (6.53-112.59)	16	
3	14 (15%)	15 (23%)		17.31 (6.33-125.40)	14	
4	26 (27%)	9 (14%)		12.05 (4.32-36.63)	9	
5	28 (29%)	13 (20%)		11.81 (1.14-54.24)	18	

Formatted: English (United States)

Formatted: Font: Bold

Formatted: English (United States)

Formatted: English (United States)

Formatted: English (United States), Not Superscript/ Subscript

Formatted: English (United States)

Formatted: English (United States), Not Superscript/ Subscript

Formatted: English (United States)

Formatted: English (United States)

Formatted Table

Formatted: Font: Bold, Font color: Auto

Viral load~~.~~

~~Up to~~ A total of 156 (44%) samples were submitted to the viral load analysis (**Table ~~3~~4**). According to age, ~~the~~ median viral load was higher in children with 4 to 6 months old (63.0 cop/cell, p= 0.007) ~~than 0 to 3 (51.41 cop/cell), 7 to 12 (39.29 cop/cell), and 12 to 36 months old (7.77), with 0.007 p-value=~~. ~~Comparing the RSV viral load and~~ Regarding ~~the~~ patient clinical conditions, we found lower viral load in patients with fever (26.15 cop/cell) than those without it (111.29 cop/cell; p=0.00) and~~.~~ higher viral load (70.24 cop/cell) ~~In addition,~~ in patients ~~that had no~~without need for oxygen therapy ~~presented higher viral load (70.24 cop/cell) than those who needed this therapy~~ (22.69; p=0.02). Deceased patients had lower viral load (2.80 cop/cell; p=0.02) in comparison to the others (37.96 cop/cell). Although lacking statistical support (p=0.089), a noteworthy observation is the tendency of lower viral load in patients with elevated CSS. The viral load analysis was performed regardless of ~~the~~ time between symptom onset and date of collection, which, in theory, could ~~disturb~~ alter the interpretation. However, of 156 samples used to measure viral titers, only 26 (16%) were collected 7 days after symptoms onset. A segmented analysis revealed very similar results when only samples collected until the 7th day of symptom onset were used. Therefore, we prefer to keep late collection patients in the analysis.

Table ~~3~~4. Comparison of viral load values between gender, race, age, outcome and clinical condition. Statistically significant p-values are highlighted in ~~red~~bold.

**Demographic data**				
**Parameter**		N	**Median (IQR)**	***p-value***
**Gender**	Male	78	51.40 (8.13-265.31)	0.08
	Female	78	24.63 (4.46-88.29)	
**Race**	White	52	54.30 (7.38-207.94)	0.09[1]
	Brown	63	16.70 (4.78-84.83)	
	Black	4	52.83 (0.30-241.27)	
**Age (months)**	0-3	86	51.40 (6.12-152.90)	**0.007[1]**

Formatted: Font: 16 pt

Formatted: Indent: First line: 0"

Formatted: Indent: First line: 0.49"

Formatted: Font: Bold, Font color: Auto

	4-6	22	63.09 (32.12-211.67)	
	7-12	21	39.29 (2.32-236.91)	
	>12	26	7.77 (1.72-36.92)	
**Outcome**	Recovery	130	37.96 (6.72-122.71)	**0.02**
	Death	7	2.80 (0.04-21.49)	
**Clinical data**				
**Fever**	Yes	121	26.15 (4.33-104.46)	**0.00**
	No	27	111.29 (51.80-408.21)	
**Cough**	Yes	144	41.53 (4.86-148.15)	0.59
	No	7	11.52 (7.98-106.29)	
**Dyspnea**	Yes	106	37.96 (3.91-154.88)	0.69
	No	40	42.05 (8.58-120.16)	
**O_2 saturation ≤ 95%**	Yes	71	26.41 (3.95-150.65)	0.40
	No	51	50.16 (8.36-196.81)	
**Respiratory distress**	Yes	115	39.29 (4.78-150.13)	0.27
	No	18	75.69 (12.66-214.26)	
**Days of hospitalization**	1-4	20	79.36 (11.10-245.08)	0.20[1]
	5-8	49	39.45 (11.89-176.21)	
	>8	54	24.42 (4.08-78.04)	
**Ventilatory support**	*No*	48	70.24 (11.41-342.96)	**0.02**
	Yes (total)		22.69	
	Yes - noninvasive	65	26.41 (6.26-105.11)	0.35
	Yes - invasive	40	17.31 (3.95-68.70)	
**Intensive Care**	Yes	82	30.01 (4.41-113.44)	0.73
	No	67	39.29 (6.90-154.61)	
**Days of Intensive Care**	1-4	20	34.74 (3.60-226.28)	0.547[1]
	5-8	16	16.27 (2.09-106.22)	
	>8	24	36.24 (9.10-106.65)	
**Days of symptom until collect**	0-3	51	36.63 (5.99-220.48)	0.19[1]
	4-6	67	39.98 (7.65-135.24)	
	7-9	24	19.98 (0.53-77.39)	
	>9	12	10.45 (3.92-50.98)	
**Subtype**	RSV-A	64	57.41	**0.03**
	RSV-B	76	27.35	

[1] Kruskal-Wallis test.
[2] IQR: interquartil range.

Viral seasonality and climatic analysis.

- In 2016 and 2017, the RSV season started in the 12th EW (March, early fall season), peaked between the 16th–20th EW and the seasonality reached its ended in the winter season, between the 310th–323th EW (**Figure 1**; **Supplementary S5 Table 6**). In

Formatted: Font: Bold, Font color: Auto
Formatted: Font: Bold
Formatted: Font: Bold, Font color: Auto
Formatted: Font: Bold, Font color: Auto
Formatted: Font: Bold
Formatted: English (United States)
Formatted: English (United States)
Formatted: English (United States)
Formatted: Font: Bold, Font color: Auto
Formatted: Centered
Formatted: Font: Bold, English (United States)
Formatted Table
Formatted: Font: 16 pt
Formatted: Indent: First line: 0"

2018, the beginning of RSV seasonality was anticipated, with the first cases occurring in 3rd EW, (January, in the middle of summer). ~~The p~~Peak happened in 14th EW and the end ~~of seasonality~~ occurred in 2~~7~~6th EW. Thus, the RSV seasonal period in 2016, 2017 and 2018 lasted ~~21~~20, ~~18~~ 19 and ~~23~~ 24 weeks, respectively.

Fig 1. Circulation of RSV-A and RSV-B between 2016 and 2018 in Espirito Santo State. The X-axis shows the epidemiological weeks (EW) for each year. The primary Y axis displays the number of positive cases for each of the subtypes and the secondary Y axis shows the values of the climatic variables. The gray zone indicates the total number of samples tested in each EW.

Precipitation rate and relative humidity percentage have not been shown to influence the distribution of RSV cases by Spearman's correlation test (p = 0.55 and 0.11, respectively). The mean temperature, however, showed a minor and inverse correlation with RSV infections (-0.16; p = 0.05).

Although RSV-A and RSV-B co-circulated in each year, it is noteworthy that~~how~~ the subtype distribution changed over the years. In 2016, RSV-B predominated (n=80; 58%) over RSV-A (n=58; 42%). In 2017 this difference increased, and RSV-B was responsible for 82% of the cases (n=63). Finally, in 2018, there was a shift in this pattern and almost all RSV cases were caused by RSV-A (n=108; 96%). Only four cases were related to RSV-B.

Phylogeny of RSV and genetic analysis.

The phylogenetic reconstructions revealed 36 RSV-A classified such as GA2.ON1 genotype, and 21 RSV-B, BA genotype, based on 2nd HVR (**S1 and S2 Figs**~~**upplementary Figure 1 and Supplementary Figure 2**~~). Some local genetic groups of both genotypes and a slightly higher diversity among the RSV-A strains (p-distance =

Formatted: Font: 16 pt, Bold

Formatted: Indent: First line: 0"

Formatted: Font: 16 pt

Formatted: Indent: First line: 0.49"

1.8%) were observed in comparison to RSV-B (p-distance = 1.6%) (**Figs~~ure~~ 2 and ~~Figure~~ 3**).

Fig 2. RSV-A phylogenetic tree. The tree was built using maximum likelihood method on MEGA 6.0 software from a MUSCLE alignment of G gene sequences of 711 bp. Previously published sequences from known genotypes were retrieved from NCBI database. The ES strains that were sequenced in this work are highlighted in bold. Numbers from 1 to 5 within the squares indicate the patients' CSS. The cross indicates patients who died due to RSV infection. The stars indicate viral load, categorized by color (in copies per cell).

Fig 3. RSV-B phylogenetic tree. The tree was built using maximum likelihood method on MEGA 6.0 software from a MUSCLE alignment of G gene sequences of 726 bp. Previously published sequences from known genotypes were retrieved from NCBI database. The ES strains that were sequenced in this work are highlighted in bold. Numbers from 1 to 5 within the squares indicate the patients' CSS. The cross indicates patients who died due to RSV infection. The stars indicate viral load, categorized by color (in copies per cell).

RSV-A ES Brazilian strains~~ from ES stat~~e, from 2016 to 2018, are clustered with strains that circulated in North America, South America, Asia, Africa and Oceania, from 2011 to 2018. A Brazilian main local cluster BR.1 (L142S, L274P, Y304H and T320A) was observed circulating from 2016 to 2018 in ES state. However two new subclusters, BR.1.1 (E106G,) and BR.1.2 (N103T, S144I, E224V, S270P and/or P298L) were detected co-circulating in ES state in 2018. Amino acid substitutions compared with the RSV-A GA2.ON1 reference strain (JN257693) can be observed in the **S6~~upplementary~~ Table~~ 7~~**. The average CSS inside BR.1 cluster was 2.84, while the average in the rest of BR strains was 3.78, showing that BR.1 cluster may be associated with lower severity disease than the other strains. ~~The v~~Viral load~~, on the other hand,~~ seemed to be higher on ~~the~~ BR.1 strains when compared to other~~the rest of~~ Brazilian strains.

~~The~~ RSV-B gene G phylogenetic reconstruction ~~of~~ ~~RSV-B gene G~~ (**Fig~~ure~~ 3**) revealed that Brazilian strains from 2016 to 2018 belonged to a cluster containing global strains circulating from 1999 to 2018. ES Brazilian strains ~~from ES state~~ were distributed through this main cluster~~,~~ and ~~they~~ presented punctual amino acid substitutions, some of

them with potential loss of O-glycosylation, such as T229N and/or S287F (strains from 2017). InsideInto this the main cluster, some local subclusters were observed, such as BR.1 (S101G loss glycosylation site, P217L and T248A loss glycosylation site) e BR.2 (G136S and S269P) in samples from 2016, reveling the large diversity among the RSV-B virus circulating in the ES State during that year. Additionally two strains from 2017 presented an insertion of tree nucleotides at codon 228. All these amino acid substitutions compared with the RSV-B BA reference strain (AY333364) are described in **S7upplementary Table 8**. CSS and viral load data were unavailable for most of RSV-B sequences, thereforeus, we could not compare those data with the genetic strains observed.

Discussion

In this paper we investigated RSV features of using Brazilian Influenza Surveillance Programthe Brazilian Influenza Surveillance System and addressed some RSV issues listed in the WHO global RSV surveillance pilot objectives [16], such as RSV burden in hospitalized children and mapping of local seasonality. Additionally, we describe the molecular characteristics of gene G and it revealed RSV-A and RSV-B local clusters co-circulating in Brazil.

RSV is prevalent in Brazilian children with SARI.

RSV prevalence in different Brazilian regions is highly diverse, ranging from 7.7% to 77.6% [25–27]. In the ES state, from 2016 to 2018, the prevalence in hospitalized children up to 3 years-old was 56%. These differences are probably related to the use of diverse methods of RSV detection (*e.g.* RT-PCR or immunofluorescence) or patient inclusion criteria (*e.g.* age, symptoms, period of the year). During 1997-98 season, Checon *et al.*

Formatted: Font: 18 pt

Formatted: Font: 16 pt, Bold

Formatted: Font: 16 pt

Formatted: Font: 16 pt, Bold

Formatted: Indent: First line: 0"

Formatted: Font: 16 pt

found a prevalence of 28% in the capital of ES State [27]. This lower prevalence in comparison to our study can be attributed to the less sensitive method used (immunofluorescence) and a broader target population age (children ≤ 5 years old).

In our study, the median age of four months in hospitalized children with RSV confirms the higher prevalence in children younger than one year old [2], which justifies why RSV vaccine candidates are aiming to protect, primarily, infants and young children [7].

The subtype but not the viral load appears to be associated with the severity of the disease.

Formatted: Font: 16 pt, Bold

Formatted: Font: 16 pt

~~Regarding~~ RSV infection~~, it~~ can cause a range of clinical outcomes [2], but ~~the~~ factors attributed to a worst outcome -remain unclear [3,4]. Several studies have shown that male gender is a risk factor for a RSV infection [2], while others have not observed such a connection [37]. Although without statistical significance, we observed that male children were slightly more affected than female, which could support the hypothesis that male children are at higher risk. Nevertheless, the CSS median was three for both genders.

Some evidences suggest that Afro-descendant children are more resistant to RSV infection than white children. [37]. In contrast, in our study, brown and black children stayed longer periods in hospital and ICU, had lower oxygen saturation and used oxygen therapy and mechanical ventilation more often than white children (**S4~~upplementary~~ Table~~ 5~~**). In addition, the prevalence of SARI caused by RSV was higher in Brazilian brown children (54%). However, it is important to consider that miscegenation of Brazilian population shows a high degree of ancestry heterogeneity both for mitochondrial and genomic DNA [38]. According to the Brazilian Institute of Geography and Statistics (IBGE), blacks and browns make up for the majority of the poverty group

in Brazil [39]. RSV lethality appears to be, at least in part, associated with socio-economic conditions, since the lethality of RSV infection in developing countries is seven fold the rate in industrialized countries [40]. Low income implies less access to basic conditions and health services, which may explain our findings.

Although some authors have found no correlation between subtypes and disease severity [41,42], many others revealed RSV-A as the most virulent subtype [9,10,12,43,44]. ~~Here, w~~We have found that children hospitalized due to SARI with RSV-A infection revealed a higher clinical score index (CSS median-=-4) – therefore, a more severe disease – when compared to those with RSV-B (CSS median–=–3). Children infected by RSV-A required O_2 therapy more often than those infected by RSV-B and, of all children who needed O_2 therapy, those affected by subgroup A needed mechanical ventilation more frequently. Although these data did not have statistical support, other studies found the same connection [9,10]. Our data also shows that children infected by subgroup A required ICU more often (p=0.03) and remained hospitalized and in ICU a day longer, on average, when compared to those infected by RSV-B, which is agreement with previous studies [44,45]. Notwithstanding, we highlight that only one genotype was found for each subtype (ON1 and BA), thus, those differences in severity could be a consequence of differences in genotypes virulence, rather than subtypes.

The correlation between disease severity and viral load remains controversial. While several authors have shown that the severity of the infection follows the viral load [46,4,5,47], others have not [7,12,42]. Some studies found associations between viral load and symptom frequency , but not severity itself [48,49]. Viral load measurement methods are widely variable between studies: some authors use plaque assay [4] or semi-quantitative analyses, such as ct [5,7,41], others use quantitative methods [47–50]. Moreover, most studies that use quantitative methods do not normalize the measurements.

Respiratory samples are naturally heterogeneous and the collection technique can influence viral genome concentration [47].

In this study ~~W~~we used a standardized method for measuring viral load. Interestingly, we found lower viral load in patients with fever (p=0.00), with need of ventilatory support (p=0.02) and in those who died (p=0.02). Our data are in conflict with previous studies that demonstrated a positive association between viral load and the presence of cough, fever [48] and the need for intubation [46]. However, ~~supporting our results,~~ two recent studies observed higher viral load in less severe RSV disease [51,52]. Piedra *et al.* observed a positive correlation between viral load and mucosal concentration of proinflammatory cytokines that may suggest that high RSV loads can protect from disease progression due to the promotion of an early robust innate immune response [51,52]. Conflicting results between studies could be attributed to the different methods used to calculate viral load, various study designs and indicators of disease severity.

The seasonal period of RSV may fluctuate and its circulation is slightly associated with temperature.

~~Regarding the RSV seasonality,~~ ~~I~~In temperate countries, RSV peak activity occurs in the winter and several studies have shown the connection between cold temperatures and ~~widespread RSV~~viral circulation [29]. In contrast, in tropical countries there is a wide range of variability in the timing and duration of epidemics and the correlation between climatic factors and viral activity is controversial [20,30]. Although in the Southern Hemisphere RSV wave usually starts between March and June and decreases between August and October [20], in Brazil, a continental country with five geographic regions, a wide variation in the seasonality is seen, such as those observed in northeastern [31] and southern [32] regions.

Formatted: Font: 16 pt, Bold

Formatted: Indent: First line: 0"

Formatted: Font: Bold

Here we showed that RSV's activity were very similar between 2016 and 2017 seasons, with the circulation onset occurring in March (EW 12), at early fall~~, peak in May (EW 16-20)~~ and end in July/August (EW ~~30~~ 31~~-and 33~~32), during the winter season. These data are in accord with the Brazilian Society of Pediatrics, which recommends the administration of Palivizumab from February to July [28]. ~~Notwithstanding~~Nonetheless, in 2018, we observed an anticipation of the seasonality onset by nine weeks, with the beginning of circulation occurring in January (S~~s~~ummer season) and with the end taking place in the ~~f~~Fall instead of W~~w~~inter.

~~In temperate countries, RSV peak activity occurs in the winter and several studies have shown the connection between cold temperatures and widespread RSV circulation [29]. In contrast, in tropical countries there is a wide range of variability in the timing and duration of epidemics and the correlation between climatic factors and viral activity is controversial [20,30]. Although in the Southern Hemisphere RSV wave usually starts between March and June and decreases between August and October [20], in Brazil, a continental country with five geographic regions, a wide variation in the seasonality is seen, such as those observed in northeastern [31] and southern [32] regions.~~ In the southeastern region it was observed that RSV ~~the~~ peak ~~of RSV~~ usually happens in early April [33]. Our data shows~~point~~ that in 2016~~-2017~~ the RSV peak occurred in May, ~~which shows~~ suggesting subtle differences even inside the same geographical region. In 2018 there was an extension of RSV'a seasonality duration ~~In 2018, we observed an anticipation of the seasonality onset by nine weeks, with the beginning of circulation occurring in January (summer season), extending the duration of RSV season~~by ~~3~~4.5 weeks when compared to the ~~RSV circulation~~ average in 2016-2017. ~~This~~ Those observations are ~~is~~ especially worrisome, since major variations could make a preventive measure harder to implement. Understanding local epidemics is important -in managing

Formatted: English (United States)

Formatted: Not Highlight

Formatted: Not Highlight

Formatted: Not Highlight

time of prophylaxis~~ at the right time~~, to support vaccine development and to follow morbidity and mortality caused by RSV infection [29], thus, establishing RSV surveillance in real time may allow for the identification of patterns and possible variation in ~~opportune ~~prophylaxis time. RSV seasonality usually lasts five to six months [20]. In our study, the longest seasonal period occurred in 2018 (~~5.75~~6 months), followed by 2016 (5~~.25 ~~ months) and 2017 (4.75 months). Interestingly, the prevalence of RSV-A was high in 2018 (96%), medium in 2016 (41%) and low in 2017 (18%). These data reinforce the theory that RSV-A may lengthen the seasonality [34].

Climatic factors, such as humidity, rainfall and temperature have been assumed to impact RSV seasonality [29,35]. However, this association remains controversial. An inverted correlation between RSV circulation, temperature and humidity was observed in a Brazilian study, carried out in São Paulo State [36]. In this study, a minor correlation was found between ~~the~~temperature decrease ~~in temperature ~~and case number ~~the~~ increase ~~in the number of cases~~. However, no correlation was found concerning humidity or precipitation.

~~Regarding RSV infection, it can cause a range of clinical outcomes [2], but the factors attributed to a worst outcome remain unclear [3,4]. Several studies have shown that male gender is a risk factor for a RSV infection [2], while others have not observed such a connection [37]. Although without statistical significance, we observed that male children were slightly more affected than female, which could support the hypothesis that male children are at higher risk. Nevertheless, the CSS median was three for both genders. Some evidences suggest that Afro-descendant children are more resistant to RSV infection than white children. [37]. In contrast, in our study, brown and black children stayed longer periods in hospital and ICU, had lower oxygen saturation and used oxygen therapy and mechanical ventilation more often than white children (**Supplementary**~~

Formatted: HTML Preformatted, Indent: First line: 0", Adjust space between Latin and Asian text, Adjust space between Asian text and numbers

Table 5). In addition, the prevalence of SARI caused by RSV was higher in Brazilian brown children (54%). However, it is important to consider that miscegenation of Brazilian population shows a high degree of ancestry heterogeneity both for mitochondrial and genomic DNA [38]. According to the Brazilian Institute of Geography and Statistics (IBGE), blacks and browns make up for the majority of the poverty group in Brazil [39]. RSV lethality appears to be, at least in part, associated with socio-economic conditions, since the lethality of RSV infection in developing countries is seven fold the rate in industrialized countries [40]. Low income implies less access to basic conditions and health services, which may explain our findings.

Although some authors have found no correlation between subtypes and disease severity [41,42], many others revealed RSV-A as the most virulent subtype [9,10,12,43,44]. Here, we found that children hospitalized due SARI with RSV-A infection revealed a higher clinical score index (CSS median = 4) – therefore, a more severe disease – when compared to those with RSV-B (CSS median = 3). Children infected by RSV-A required O_2-therapy more often than those infected by RSV-B and, of all children who needed O_2-therapy, those affected by subgroup A needed mechanical ventilation more frequently. Although these data did not have statistical support, other studies found the same connection [9,10]. Our data also shows that children infected by subgroup A required ICU more often (p=0.03) and remained hospitalized and in ICU a day longer, on average, when compared to those infected by RSV-B, which is agreement with previous studies [44,45]. Notwithstanding, we highlight that only one genotype was found for each subtype (ON1 and BA), thus, those differences in severity could be a consequence of differences in genotypes virulence, rather than subtypes.

The correlation between disease severity and viral load remains controversial. While several authors have shown that the severity of the infection follows the viral load

~~[46,4,5,47], others have not [7,12,42]. Some studies found associations between viral load and symptom frequency , but not severity itself [48,49]. Viral load measurement methods are widely variable between studies: some authors use plaque assay [4] or semi-quantitative analyses, such as ct [5,7,41], others use quantitative methods [47–50]. Moreover, most studies that use quantitative methods do not normalize the measurements. Respiratory samples are naturally heterogeneous and the collection technique can influence viral genome concentration [47].~~

~~We used a standardized method for measuring viral load. Interestingly, we found lower viral load in patients with fever (p=0.00), with need of ventilatory support (p=0.02) and in those who died (p=0.02). Our data are in conflict with previous studies that demonstrated a positive association between viral load and the presence of cough, fever [48] and the need for intubation [46]. However, supporting our results, two recent studies observed higher viral load in less severe RSV disease [51,52]. Piedra *et al.* observed a positive correlation between viral load and mucosal concentration of proinflammatory cytokines that may suggest that high RSV loads can protect from disease progression due to the promotion of an early robust innate immune response [51,52]. Conflicting results between studies could be attributed to the different methods used to calculate viral load, various study designs and indicators of disease severity.~~

ON1 and BA were the only genotypes detected.

~~Regarding molecular characterization, a~~All RSV-A isolates were ON1 genotype and all RSV-B were BA, which confirm the fast-global dissemination of RSV with nucleotide duplication. These findings are ~~and are~~ consistent with recent published reports performed in other countries, such as Philippines [53], Kenya [54], Italy [55], USA and Puerto Rico [56].

Formatted: English (United States)

Formatted: English (United States)

Formatted: Font: 16 pt

Formatted: Indent: First line: 0"

Formatted: English (United States)

Overall p-distance during the study period in RSV-A was 1.8%. A recent study observed an overall p-distance of 1.4% within ON1 [13]. A noteworthy observation is the fact that in 2017 we found the lowest prevalence of RSV-A in ES (18%), and, still, the highest genetic diversity. Phylogeny showed that 2017 strains were distributed in almost all genetic clusters, which showed high diversity that year. RSV-A phylogenetic analysis revealed ongoing genetic changes, with BR.1 grouping most recent strains, suggesting that BR.1 strains may be under positive selective pressure. Changes in the circulation of RSV strains have been considered a mechanism for evading immune response generated by previous strains, which possibly allows for re-infections to occur [57].

~~Some evidences show that the replicative ability and, thereforethus, the viral load, areis driven by viral genetic factors, which explains why some genetic groups exhibit low viral loads while others show high viral loads [58]. We noticed that BR.1 grouped many strains that showed moderate/high viral titer, while others exhibit lower viral loads. The lower CSS found in BR.1, compared to other strains, strengthens the inversely proportional link between severity and viral load and suggests that 2018 circulating strains were less virulent. However, more studies with a larger sample size are needed to confirm this hypothesis.~~

As demonstrated, in 2018 RSV-B was responsible for only 4% of cases. Therefore, phylogenetic analysis did not include any RSV-B samples from that year. Older strains, from 2009 to 2014, are positioned at the base of the BA cluster, however, sample strains collected between 2015 and 2018 did not form genetic groups related to the year of collection. This observation may suggest an absence of positive pressure. ~~Since few strains sequenced from RSV-B had their viral load measured, it was not possible to analyze the distribution of viral titer among clades.~~

Although we found clusters composed exclusively of ES samples, it is necessary to expand the sequencing of RSV samples globally in order to verify if there is in fact the formation of local genetic groups or if the observation is caused by a sample bias.

Previous studies show that a large part of the genetic variability between RSV strains comes from changes in O-glycosylation profile and that this may be associated with an evolutionary mechanism of immune response evasion [59]. Here, we investigated and listed strain amino acid substitutions and also those shared within and between clusters. However, we did not carry out in-depth analysis in order to understand the role of these mutations, therefore, our objective was purely observational. Among the mutations found, one of the most interesting was the insertion of tree nucleotides at codon 228 in RSV-B. Further studies are essential to understand virus evolution and pathogenicity mutation consequences.

Limitations of this study include the fact that the majority of patients had an acute infection, thus, the prevalence found refers only to SARI, and the absence of a mild infection group prevents further analysis of severity influencing factors. Lastly, clinical data were taken from notification forms, which often show inconsistencies and missing data. Despite those caveats, we believe the data provide valuable epidemiological, genetic and clinical information on RSV.

Conclusion

Formatted: Font: 18 pt

In this study we observed a high prevalence of RSV in children under three years old even when using the Brazilian Influenza ~~surveillance~~ Surveillance ~~system~~Program. This result is important because it shows that the establishment of global RSV surveillance within the Influenza surveillance system allows for the detection of a large

number of cases.- Our data suggest that RSV-A is, in fact, more virulent than RSV-B. Notably, no correlation between viral load and disease severity was observed. The observation of an important anticipation of the seasonal period is worrisome, since this can make it difficult to administer prophylactic measures at the right time, however, it is necessary to expand the historical series of seasonality in Espirito Santo. The average temperature was the only climatic factor to show interference with the viral circulation. Our data show the annual co-circulation of RSV-A and RSV-B, however, with considerable fluctuations in the prevalence of subtypes. ON1 and BA were the only ones found in the studied period, which corroborates with a series of recent studies. The establishment of a global and standardized real time RSV surveillance may allow for the collection of data that will help understanding the complex mechanisms of viral evolution and will facilitate the development of future vaccines and antiviral drugs. RSV continues to lead the cause of hospitalizations for pneumonia in children worldwide, being responsible for a large fraction of morbidity and mortality in the pediatric population.

Acknowledgments

Formatted: Font: 18 pt

We would like to thank Liliana Cruz Spano for her significant theoretical and experimental support to this work, all researchers who upload genetic sequences in the public genetic database – GenBank, patients, parents and guardians, the Espirito Santo State Health Department and the Brazilian Ministry of Health, represented by the Influenza Technical Group.

References

Formatted: Font: 18 pt

1. Hall CB, Weinberg GA, Iwane MK, Blumkin AK, Edwards KM, Staat MA, et al. The Burden of Respiratory Syncytial Virus Infection in Young Children. N Engl J Med. 2009;360: 588–598. doi:10.1056/NEJMoa0804877

2. Borchers AT, Chang C, Gershwin ME, Gershwin LJ. Respiratory Syncytial Virus—A Comprehensive Review. Clin Rev Allergy Immunol. 2013;45: 331–379. doi:10.1007/s12016-013-8368-9

3. Collins PL, Graham BS. Viral and Host Factors in Human Respiratory Syncytial Virus Pathogenesis. J Virol. 2008;82: 2040–2055. doi:10.1128/JVI.01625-07

4. El Saleeby CM, Bush AJ, Harrison LM, Aitken JA, DeVincenzo JP. Respiratory Syncytial Virus Load, Viral Dynamics, and Disease Severity in Previously Healthy Naturally Infected Children. J Infect Dis. 2011;204: 996–1002. doi:10.1093/infdis/jir494

5. Hasegawa K, Jartti T, Mansbach JM, Laham FR, Jewell AM, Espinola JA, et al. Respiratory Syncytial Virus Genomic Load and Disease Severity Among Children Hospitalized With Bronchiolitis: Multicenter Cohort Studies in the United States and Finland. J Infect Dis. 2015;211: 1550–1559. doi:10.1093/infdis/jiu658

6. Resa C, Magro S, Marechal P, Barranger C, Joannes M, Miszczak F, et al. Development of an efficient qRT-PCR assay for quality control and cellular quantification of respiratory samples. J Clin Virol. 2014;60: 270–275. doi:10.1016/j.jcv.2014.03.019

7. Mazur NI, Higgins D, Nunes MC, Melero JA, Langedijk AC, Horsley N, et al. The respiratory syncytial virus vaccine landscape: lessons from the graveyard and promising candidates. Lancet Infect Dis. 2018;18: e295–e311. doi:10.1016/S1473-3099(18)30292-5

8. Mufson MA, Orvell C, Rafnar B, Norrby E. Two Distinct Subtypes of Human Respiratory Syncytial Virus. J Gen Virol. 1985;66: 2111–2124. doi:https://doi.org/10.1099/0022-1317-66-10-2111

9. McConnochie KM, Hall CB, Walsh EE, Roghmann KJ. Variation in severity of respiratory syncytial virus infections with subtype. J Pediatr. 1990;117: 52–62. doi:10.1016/S0022-3476(05)82443-6

10. Walsh EE, McConnochie KM, Long CE, Hall CB. Severity of Respiratory Syncytial Virus Infection Is Related to Virus Strain. J Infect Dis. 1997;175: 814–820. doi:10.1086/513976

11. Hornsleth A, Klug B, Nir M, Johansen J, Hansen K, Christensen L, et al. Severity of respiratory syncytial virus disease related to type and genotype of virus and to cytokine values in nasopharyngeal secretions. Pediatr Infect Dis J. 1998;17: 1114–1121. doi:10.1097/00006454-199812000-00003

12. Kim Y-I, Murphy R, Majumdar S, Harrison LG, Aitken J, DeVincenzo JP. Relating plaque morphology to respiratory syncytial virus subgroup, viral load, and disease severity in children. Pediatr Res. 2015;78: 380–388. doi:10.1038/pr.2015.122

13. Muñoz-Escalante JC, Comas-García A, Bernal-Silva S, Robles-Espinoza CD, Gómez-Leal G, Noyola DE. Respiratory syncytial virus A genotype classification based on systematic intergenotypic and intragenotypic sequence analysis. Sci Rep. 2019;9: 20097. doi:10.1038/s41598-019-56552-2

14. Trento A, Viegas M, Galiano M, Videla C, Carballal G, Mistchenko AS, et al. Natural History of Human Respiratory Syncytial Virus Inferred from Phylogenetic Analysis of the Attachment (G) Glycoprotein with a 60-Nucleotide Duplication. J Virol. 2006;80: 975–984. doi:10.1128/JVI.80.2.975-984.2006

15. Eshaghi A, Duvvuri VR, Lai R, Nadarajah JT, Li A, Patel SN, et al. Genetic Variability of Human Respiratory Syncytial Virus A Strains Circulating in Ontario: A Novel Genotype with a 72 Nucleotide G Gene Duplication. PLoS ONE. 2012;7: e32807. doi:10.1371/journal.pone.0032807

16. WHO strategy to pilot global respiratory syncytial virus surveillance based on the Global Influenza Surveillance and Response System (GISRS). Geneva: World Health Organization; 2017. Licence: CC BY-NC-SA 3.0 IGO.

17. Brasil. Panorama do Espírito Santo [cited 27 September 2020]. In Instituto Brasileiro de Geografia e Estatística (IBGE) [Internet]. Available from: https://cidades.ibge.gov.br/brasil/es/panorama.

18. Brasil. Gripe (influenza): causas, sintomas, tratamento, diagnóstico e prevenção. [cited 08 September 2020]. In: Brazilian Ministry of Health [Internet]. Available from: https://antigo.saude.gov.br/saude-de-a-z/gripe/#boletins.

19. Martinello RA, Chen MD, Weibel C, Kahn JS. Correlation between Respiratory Syncytial Virus Genotype and Severity of Illness. J Infect Dis. 2002;186: 839–842. doi:10.1086/342414

21. Obando-Pacheco P, Justicia-Grande AJ, Rivero-Calle I, Rodríguez-Tenreiro C, Sly P, Ramilo O, et al. Respiratory Syncytial Virus Seasonality: A Global Overview. 2018; 217(9): 1356-1364. doi:10.1093/infdis/jiy056.

21. Álvarez-Argüelles ME, Oña-Navarro M de, Rojo-Alba S, Torrens-Muns M, Junquera-Llaneza ML, Antonio-Boga J, et al. Quantification of human papilloma virus (HPV) DNA using the Cobas 4800 system in women with and without pathological alterations attributable to the virus. J Virol Methods. 2015;222: 95–102. doi:10.1016/j.jviromet.2015.05.016

22. Sequencher® - DNA sequence analysis software. Ann Arbor, MI USA: Gene Codes Corporation;

23. Tamura K, Stecher G, Peterson D, Filipski A, Kumar S. MEGA6: Molecular Evolutionary Genetics Analysis Version 6.0. Mol Biol Evol. 2013;30: 2725–2729. doi:10.1093/molbev/mst197

24. Guindon S, Gascuel O. A Simple, Fast, and Accurate Algorithm to Estimate Large Phylogenies by Maximum Likelihood. Syst Biol. 2003;52: 696–704. doi:10.1080/10635150390235520

25. Gardinassi L, Simas P, Gomes D, Bonfim C, Nogueira F, Garcia G, et al. Diversity and Adaptation of Human Respiratory Syncytial Virus Genotypes Circulating in Two Distinct Communities: Public Hospital and Day Care Center. Viruses. 2012;4: 2432–2447. doi:10.3390/v4112432

26. Vieira SE, Thomazelli LM, de Paulis M, Ferronato AE, Oliveira DB, Martinez MB, et al. Infections Caused by HRSV A ON1 Are Predominant among Hospitalized Infants with Bronchiolitis in São Paulo City. BioMed Res Int. 2017;2017: 1–7. doi:10.1155/2017/3459785

27. Checon RE, Siqueira MM, Lugon AK, Portes S, Dietze R. Short report: seasonal pattern of respiratory syncytial virus in a region with a tropical climate in southeastern Brazil. Am J Trop Med Hyg. 2002;67: 490–491. doi:10.4269/ajtmh.2002.67.490

28. Kfouri RA, Sadeck LSR, Moura AA, Bresolin AC, Miralha AL, Pimentel AM, et al. Diretrizes para o manejo da infecção causada pelo Vírus Sincicial Respiratório (VSR). Soc Bras Pediatr. 2017; 1–20.

29. Haynes AK, Manangan AP, Iwane MK, Sturm-Ramirez K, Homaira N, Brooks WA, et al. Respiratory Syncytial Virus Circulation in Seven Countries With Global Disease Detection Regional Centers. J Infect Dis. 2013;208: S246–S254. doi:10.1093/infdis/jit515

30. Bloom-Feshbach K, Alonso WJ, Charu V, Tamerius J, Simonsen L, Miller MA, et al. Latitudinal Variations in Seasonal Activity of Influenza and Respiratory Syncytial Virus (RSV): A Global Comparative Review. PLoS ONE. 2013;8: e54445. doi:10.1371/journal.pone.0054445

31. Moura FEA, Borges LC, Portes SAR, Ramos EAG, Siqueira MM. Respiratory syncytial virus infections during an epidemic period in Salvador, Brazil: viral antigenic group analysis and description of clinical and epidemiological aspects. Mem Inst Oswaldo Cruz. 2003;98: 739–743. doi:10.1590/S0074-02762003000600005

32. Straliotto SM, Siqueira MM, Muller RL, Fischer GB, Cunha MLT, Nestor SM. Viral etiology of acute respiratory infections among children in Porto Alegre, RS, Brazil. Rev Soc Bras Med Trop. 2002;35: 283–291. doi:10.1590/S0037-86822002000400002

33. Freitas ARR, Donalisio MR. Respiratory syncytial virus seasonality in Brazil: implications for the immunisation policy for at-risk populations. Mem Inst Oswaldo Cruz. 2016;111: 294–301. doi:10.1590/0074-02760150341

34. Yu J, Liu C, Xiao Y, Xiang Z, Zhou H, Chen L, et al. Respiratory Syncytial Virus Seasonality, Beijing, China, 2007–2015. Emerg Infect Dis. 2019;25: 1127–1135. doi:10.3201/eid2506.180532

35. Zhang H, Wen S, Zheng J, Chen X, Lv F, Liu L. Meteorological factors affecting respiratory syncytial virus infection: A time- series analysis. Pediatr Pulmonol. 2020;55: 713–718. doi:10.1002/ppul.24629

36. Gardinassi LG, Simas PVM, Salomão JB, Durigon EL, Trevisan DMZ, Cordeiro JA, et al. Seasonality of viral respiratory infections in Southeast of Brazil: the influence of temperature and air humidity. Braz J Microbiol. 2012;43: 98–108. doi:10.1590/S1517-83822012000100011

37. Bradley JP, Bacharier LB, Bonfiglio J, Schechtman KB, Strunk R, Storch G, et al. Severity of Respiratory Syncytial Virus Bronchiolitis Is Affected by Cigarette Smoke Exposure and Atopy. Pediatrics. 2005;115: e7–e14. doi:10.1542/peds.2004-0059

38. Cardena MMSG, Ribeiro-dos-Santos Â, Santos S, Mansur AJ, Pereira AC, Fridman C. Assessment of the Relationship between Self-Declared Ethnicity, Mitochondrial Haplogroups and Genomic Ancestry in Brazilian Individuals. PLoS ONE. 2013;8: e62005. doi:10.1371/journal.pone.0062005

39. Brasil. Síntese de Indicadores Sociais: uma análise das condições de vida da população brasileira [cited 27 September 2020]. In Instituto Brasileiro de Geografia e Estatística (IBGE) [Internet]. Available from: https://biblioteca.ibge.gov.br/visualizacao/livros/liv101629.pdf.

40. Nair H, Nokes DJ, Gessner BD, Dherani M, Madhi SA, Singleton RJ, et al. Global burden of acute lower respiratory infections due to respiratory syncytial virus in young children: a systematic review and meta-analysis. Lancet. 2010;375: 1545–1555. doi:10.1016/S0140-6736(10)60206-1

41. Espinosa Y, San Martín C, Torres A, Farfán M, Torres J, Avadhanula V, et al. Genomic Loads and Genotypes of Respiratory Syncytial Virus: Viral Factors during Lower Respiratory Tract Infection in Chilean Hospitalized Infants. Int J Mol Sci. 2017;18: 654. doi:10.3390/ijms18030654

42. Rodriguez-Fernandez R, Tapia LI, Yang C-F, Torres JP, Chavez-Bueno S, Garcia C, et al. Respiratory Syncytial Virus Genotypes, Host Immune Profiles, and Disease Severity in Young Children Hospitalized With Bronchiolitis. J Infect Dis. 2018;217: 24–34. doi:10.1093/infdis/jix543

43. Tran DN, Pham TMH, Ha MT, Tran TTL, Dang TKH, Yoshida L-M, et al. Molecular Epidemiology and Disease Severity of Human Respiratory Syncytial Virus in Vietnam. PLoS ONE. 2013;8: e45436. doi:10.1371/journal.pone.0045436

44. Hall CB, Walsh EE, Schnabel KC, Long CE, McConnochie KM, Hildreth SW, et al. Occurrence of Groups A and B of Respiratory Syncytial Virus over 15 Years: Associated Epidemiologic and Clinical Characteristics in Hospitalized and Ambulatory Children. J Infect Dis. 1990;162: 1283–1290. doi:10.1093/infdis/162.6.1283

45. Jafri HS, Wu X, Makari D, Henrickson KJ. Distribution of Respiratory Syncytial Virus Subtypes A and B Among Infants Presenting to the Emergency Department With Lower Respiratory Tract Infection or Apnea. Pediatr Infect Dis J. 2013;32: 335–340. doi:10.1097/INF.0b013e318282603a

46. DeVincenzo JP, Wilkinson T, Vaishnaw A, Cehelsky J, Meyers R, Nochur S, et al. Viral Load Drives Disease in Humans Experimentally Infected with Respiratory

Syncytial Virus. Am J Respir Crit Care Med. 2010;182: 1305–1314. doi:10.1164/rccm.201002-0221OC

47. Gómez- Novo M, Boga JA, Álvarez- Argüelles ME, Rojo- Alba S, Fernández A, Menéndez MJ, et al. Human respiratory syncytial virus load normalized by cell quantification as predictor of acute respiratory tract infection. J Med Virol. 2018;90: 861–866. doi:10.1002/jmv.25020

48. Hijano DR, Brazelton de Cardenas J, Maron G, Garner CD, Ferrolino JA, Dallas RH, et al. Clinical correlation of influenza and respiratory syncytial virus load measured by digital PCR. PLoS ONE. 2019;14: e0220908. doi:10.1371/journal.pone.0220908

49. Moreira FB, Rosario CS, Santos JS, Avanzi VM, Nogueira MB, Vidal LR, et al. Molecular characterization and clinical epidemiology of human respiratory syncytial virus (HRSV) A and B in hospitalized children, Southern Brazil. J Med Virol. 2017;89: 1489–1493. doi:10.1002/jmv.24795

50. Fodha I, Vabret A, Ghedira L, Seboui H, Chouchane S, Dewar J, et al. Respiratory syncytial virus infections in hospitalized infants: Association between viral load, virus subgroup, and disease severity. J Med Virol. 2007;79: 1951–1958. doi:10.1002/jmv.21026

51. Garcia-Mauriño C, Moore-Clingenpeel M, Thomas J, Mertz S, Cohen DM, Ramilo O, et al. Viral Load Dynamics and Clinical Disease Severity in Infants With Respiratory Syncytial Virus Infection. J Infect Dis. 2019;219: 1207–1215. doi:10.1093/infdis/jiy655

52. Piedra F-A, Mei M, Avadhanula V, Mehta R, Aideyan L, Garofalo RP, et al. The interdependencies of viral load, the innate immune response, and clinical outcome in children presenting to the emergency department with respiratory syncytial virus-associated bronchiolitis. PLoS ONE. 2017;12: e0172953. doi:10.1371/journal.pone.0172953

53. Okamoto M, Dapat CP, Sandagon AMD, Batangan-Nacion LP, Lirio IC, Tamaki R, et al. Molecular Characterization of Respiratory Syncytial Virus in Children With Repeated Infections With Subgroup B in the Philippines. J Infect Dis. 2018;218: 1045–1053. doi:10.1093/infdis/jiy256

54. Otieno JR, Kamau EM, Oketch JW, Ngoi JM, Gichuki AM, Binter Š, et al. Whole genome analysis of local Kenyan and global sequences unravels the epidemiological and molecular evolutionary dynamics of RSV genotype ON1 strains. Virus Evol. 2018;4. doi:10.1093/ve/vey027

55. Esposito S, Piralla A, Zampiero A, Bianchini S, Di Pietro G, Scala A, et al. Characteristics and Their Clinical Relevance of Respiratory Syncytial Virus Types and Genotypes Circulating in Northern Italy in Five Consecutive Winter Seasons. PLoS ONE. 2015;10: e0129369. doi:10.1371/journal.pone.0129369

56. Bin Lu, Liu H, Tabor DE, Tovchigrechko A, Qi Y, Ruzin A, et al. Emergence of new antigenic epitopes in the glycoproteins of human respiratory syncytial virus

collected from a US surveillance study, 2015–17. Sci Rep. 2019;9: 3898. doi:10.1038/s41598-019-40387-y

57. Hall CB. Respiratory Syncytial Virus and Parainfluenza Virus. N Engl J Med. 2001;344: 1917–1928. doi:10.1056/NEJM200106213442507

58. Elawar F, Griffiths CD, Zhu D, Bilawchuk LM, Jensen LD, Forss L, et al. A Virological and Phylogenetic Analysis of the Emergence of New Clades of Respiratory Syncytial Virus. Sci Rep. 2017;7: 12232. doi:10.1038/s41598-017-12001-6

59. Leemans A, Boeren M, Van der Gucht W, Martinet W, Caljon G, Maes L, et al. Characterization of the role of N-glycosylation sites in the respiratory syncytial virus fusion protein in virus replication, syncytium formation and antigenicity. Virus Res. 2019;266: 58–68. doi:10.1016/j.virusres.2019.04.006

Formatted: English (United States)

Supporting information captions

Formatted: Font: 18 pt

S1upplementary Figure 1. RSV-A phylogenetic tree based on 336 bp of the HVR-2 of G gene. The tree was built using maximum likelihood method on MEGA 6.0 software from a MUSCLE alignment, with some manual editions. Reference sequences from each described genotype were downloaded from NCBI GenBank and used in the phylogenetic reconstruction. The genotypes were classified by colors and all ES strains grouped within the ON1 genotype.

S2upplementary Figure 2. RSV-A phylogenetic tree based on 318 bp of the HVR-2 of G gene. The tree was built using maximum likelihood method on MEGA 6.0 software from a MUSCLE alignment, with some manual editions. Reference sequences from each described genotype were downloaded from NCBI GenBank and used in the phylogenetic reconstruction. The genotypes were classified by colors and all ES strains grouped within the BA genotype.

S1upplementary Table 1. Primers, probes and DNA fragments used in our study. “F”, “R” and “P”, represent the sequence of the forward and reverse primers and the probe, respectively. Synthetic DNA fragment from RSV was included in a pMA-t vector.

Supplementary S2 Table 2. List of the sequences used to build the phylogeny based on HVR-2 of gene G for both subtypes RSA-A and RSV-B.

S3upplementary Table 3. List of the sequences used to build the phylogeny based on gene G for both subtypes RSV-A and RSV-B. Collection date of some sequences were unavailable.

Supplementary Table 4. Clinical Severity Score: the score varied between 0 and 5. Higher values were assumed to represent more severe illness. Need for ICU, O_2≤95%, length in hospital>5 days and requirement of O_2 therapy accounted for 1 point each. Need for mechanical ventilation accounted for 2 points. Patients infected with RSV-A were most commonly classified into the most severe scores. The difference between viral loads was not related to severity, but there was no statistical significance.

S4upplementary Table 5. Differences in severity among ethnicities showing that children classified as black or brown showed O_2 saturation ≤ 95% and respiratory distress more often than those classified as white. Also they required ventilator support more frequently and stand more time in hospital and in ICU.

S5upplementary Table 6. Duration and climatic characteristics of RSV seasonality in the years studied.

S6~~upplementary~~ Table ~~7~~. List of amino acid changes in RSV-A. Residues in blue and red show potential losses and gains of O-glycosylation sites, respectively.

S7~~upplementary~~ Table ~~8.~~ List of amino acid changes in RSV-B. Residues in blue and red show potential losses and gains of O-glycosylation sites, respectively.

Response to Reviewers

Dear editors and reviewers, thank you for your attention in improving this study. Here are the answers for each observation.

Sincerely,
Lucas Alves Vianna

Editor comments:

Comment 1: Please ensure that your manuscript meets PLOS ONE's style requirements, including those for file naming. The PLOS ONE style templates can be found at:

https://journals.plos.org/plosone/s/file?id=wjVg/PLOSOne_formatting_sample_main_body.pdf and

https://journals.plos.org/plosone/s/file?id=ba62/PLOSOne_formatting_sample_title_authors_affiliations.pdf

Answer: After a careful review, we have modified the formatting of the headings and legends of the supplementary figures and tables to meet PLOS ONE's style requirements. We also corrected some tables that presented values highlighted in red, contrary to the rules of PLOS ONE. Finally, we increased the font size of the Materials and methods, Results and Discussion subheadings to 16 pt, according to rules.

Comment 2: We note that you included minors (age<18) in your study. Please provide additional details regarding minor's consent. In the ethics statement in the Methods and online submission information, please ensure that you have specified whether you obtained consent from parents or guardians. If the need for consent was waived by the ethics committee, please include this information."

Answer: We agree with this observation. The sentence "The need for parents or guardians' consent was waived by the ethics committee." was included in "Ethics Statement" section. Please, check the lines 207-208.

Reviewer #1 comments:

1. **Title - Revise the title to reflect the key findings of the research.**

Answer: To address this comment, we have changed the title to: "Seasonality, molecular epidemiology and virulence of Respiratory Syncytial Virus (RSV): a perspective into the Brazilian Influenza Surveillance Program". However, considering that this study addressed several aspects of RSV, the inclusion of key findings would make the title too large and we have opted to make it shorter and easier to read.

2. **Introduction - Revise the introduction and make it shorter and its current form it distracts the reader.**

   Answer: We have changed the introduction and eliminated sentences that, although interesting, would not affect the understanding of the objectives. However, Introduction size reduction was small. It turns out that this study has addressed multiple aspects of RSV (*e.g.:* prevalence, association between severity and subtypes, viral load, seasonality and association with climatic factors and phylogenetic aspects), as we understand that these are important aspects to discuss.

3. **Methodology - Organize the Methodology - Study population, experimental methods - RT-qPCR, sequencing etc. in a brief form that gives a good understanding of the sequence of events with relevant methodology.**

   Answer: We have changed the way methods are presented, in order to maintain the same pattern presented at the results.

   Thus, methods are now as follows:

   1. Population sampling, study period and location
   2. RSV and Influenza detection and subtyping
   3. Clinical and epidemiological data collection
   4. Viral load quantification
   5. Climate data collection
   6. Partial amplification and sequencing of glycoprotein gene
   7. RSV genotyping and gene G phylogenetic reconstruction
   8. Statistical treatment.
   9. Data availability
   10. Ethics Statement

4. **Results - They need to be organized in the order of appearance as in the Methodology. You do not have to replicate all the information given in the Tables in the texts. Redundancy also distracts the reader and I found it difficult to organize the results to understand the authors' way of cohesion.**

Answer: We think this comment will make the manuscript easier to read. As recommended, we have reorganized the objectives in the same pattern presented in the results, and eliminated redundant information.

5. **Discussion - Again follow the order of your results in Discussion.**

   Answer: We have organized the discussion in the same order presented in Methods and Results and created subsections in the discussion, in order to improve reading.

6. **Overall - You must revise this manuscript shortening certain sections and organizing the manuscript from I to D. Otherwise the results produced cannot be understood by the authors.**

   Answer: To address this recommendation, we have reduced the text as much as possible, without interfering with data presentation quality and consistency. We have reduced redundancies in the results (data being presented in the text and table) and removed some excerpts throughout the manuscript that we consider less relevant.

7. **Language must be clear, correct, and unambiguous. At its current form it is difficult to follow the authors. Please also look into typographical or grammatical errors when your revise the manuscript. You may ask a native speaker to read the manuscript after fixing all the issues indicated.**

   Answer: we have asked a native speaker to thoroughly review the manuscript.

8. **Please follow the PLOS ONE formatting guidelines well before you submit after revision.**

   Answer: We have done so.

Reviewer #2 comments:

1. **The authors present generalized conclusions that are not specific to the Brazilian aspect or timeframe on which this study is based. Key results to support the identified objectives are not highlighted in the abstract or the conclusion (e.g. the influence of climate factors on RSV seasonality and the role of genetic diversity of RSV on disease severity). Clinical severity scores referenced in the abstract and results to support interpretations of the role of**

viral load and genetic diversity of RSV on disease severity, should be presented in the main tables/figures of the manuscript as opposed to supplemental. The authors should revise these areas and sharpen the focus of their Discussion through reduction to improve readability and presentation of key messages of RSV surveillance in Brazil between 2016-2018 relative to previous observations in Brazil or other parts of the world during the similar timeframe.

Answer: We appreciate the observations. However, some results of this study are not related to a specific location or timeframe. The correlation analyzes between viral load, genetic differences and severity are examples. These results possibly transcends the time and place of the study and, therefore, are not specific to the Brazilian aspect or timeframe.

As recommended, we have included the key results in both abstract and conclusion. We also transformed supplementary table 4 into Table 3. Previous table 3, which presented data on viral load, is now part of Table 4.

Given the different approaches taken in the study, we chose to divide the discussion into topics, in the hope of improving the quality of reading and regarding the discussion length, we removed some less important passages in order to improve the readability and presentation of key messages of the study.

2. **The current title (and abstract) fail to address the presented timeframe of RSV surveillance or what aspects of "landscape" or "perspectives" the authors are referring to relative to their objectives and results. The authors should consider revision.**

   Answer: as changing the Title was also a recommendation of Reviewer 1, and to clarify which aspects of the "landscape" the study focused on, we have changed the title to: "Seasonality, molecular epidemiology and virulence of Respiratory Syncytial Virus (RSV): a perspective into the Brazilian Influenza Surveillance Program". We hope this new title is suitable for both Reviewers.

3. **Figure 2 and Figure 3 are out of focus and uninterruptable for review. The authors should revise.**

   Answer: Figures 2 and 3 were redone to improve quality and readability. We decided to change the size and save the file as .EPS.

4. **Line 90-92, 130-133, and 270-276. The authors statement of "seasonal oscillation" (Line 90-92) is not supported by their main observations (Line 270-276) from Fig. 1, and in turn, their statement regarding "creating difficulties for determination of the most appropriate period to start prophylaxis" is not substantiated and is in contrast to their later statement of "recommends the administration of palivizumab from February to July" (Line 359). The authors state (Line 130-133) that "seasonality onset and end were defined as the first and last of 2 consecutive weeks, respectively, when the number of RSV cases exceeded 10% of the number detected during the RSV peak week" and reference Obando-Pacheco et al 2018 [21]. However, Obando-Pacheco et al 2018 states that "the onset of RSV season was defined as the first 2 consecutive weeks when >10% of the total tested samples for respiratory pathogens were positive for RSV. The end of the RSV season was defined similarly as when the proportion of positive RSV tests fell below 10% for 2 consecutive weeks.". Given the impact of molecular testing on determining RSV seasonality, the authors should revise their analysis and adopt a more accepted threshold for seasonality assessment based on %RSV positive cases as opposed to the number of RSV cases to support a potential interpretation of "seasonal oscillation" (see also Midgley et al. 2017 JID 216(3):345-355).**

   Answer: We agree with the Reviewer, and, in fact, there was a misinterpretation of season beginning and end definition by Obando-Pacheco et al. (2018). Therefore, we reviewed the data and corrected the analysis. However, there were no changes in season onset in any year, but there were small changes in season end, as described below:

   1. End in 2016: from EW 33 to EW 32.
   2. End in 2017: from EW 30 to EW 31.
   3. End in 2018: from EW 26 to EW 27.

   Although the reviewer understood that the data do not support the claim that there was a fluctuation in season period during the study, we would like to point out that in 2016 and 2017 the RSV seasonal period started at epidemiological week (EW) 12 and ended at EW 32 and 31, respectively. In contrast, in 2018 the season was anticipated to EW 3, which is 9 weeks before the start in previous years. Season end was also anticipated to EW 27. There are the reasons why we understand there was an oscillation in the seasonal period during our study. As Palivizumab is administered in five consecutive monthly doses and considering that the first dose should be administered one month before season start, this oscillation may have an impact in the administration of prophylactic drugs.

   In order to make this point clearer, we have restructured the discussion paragraphs.

5. **Table 1: The authors should revise this Table to provide both numerators and denominators to allow for readability and logical follow with the main text. This will also allow the reader to appropriately follow the statistical assessment employed of relative proportions. In addition, Influenza prevalence is noted in the main text, but not in the corresponding Table 1. The authors should to revise the Table to include all relevant data for the reader.**

   Answer: we have revised Table 1, making the requested changes.

6. **Line 225-232 and Table 2: The authors should rephrase their statement regarding "clinical features of patients affect by RSV" to better reflect clinical characteristics of patients with SARI, since clinical data are presented for the total 632 patients and the 327 patients with RSV (180 RSV-A and 147 RSV-B). The numbers and percentages in the main text reflect the total population (N=632) and not the population of patients with RSV disease (N=327). The authors should further revise this Table to provide both numerators and denominators to allow for readability and logical follow with the main text. This will also allow the reader to appropriately follow the statistical assessment employed of relative proportions and to distinguish between RSV and everything else. Finally, viral load data in Table 2 is out of place without a (%) and should be included in Table 3 where viral load values are presented.**

   Answer: Table 2 and the associated text present clinical data only of RSV infected patients. This table had an error in the "Sample number" field, which contained the total number of samples studied (632), however, the analyzes were performed only with the 352 RSV positive children. The error has been corrected. We took the opportunity to correct some fields containing three decimal places, standardizing the values to two decimal places. We have also relocated table 2 viral load data to table 3, as suggested. Finally, we included denominators to facilitate data interpretation.

Minor Comments for Author (Required)

7. **Line 17 and 40. The authors are repetitive in their statements in the Background and Conclusion sections of their Abstract regarding "understanding seasonality, genetic features…may support antiviral and vaccine development. The authors should revise the abstract and clarify how the results of this study specifically support antiviral and vaccine development.**

   Answer: We have eliminated the redundant part from the "Background" and briefly discussed how seasonal period, virulence and genetic diversity can assist in the development and application of vaccines and antiviral drugs.

8. **Lines 21, 38, 81, 83, 88, 339-440. Is the Brazilian Influenza Surveillance Program part of WHO's Global Respiratory Syncytial Virus Surveillance Pilot and/or the Global Influenza Surveillance and Response System (GISRS)? The authors should consider revising for clarity; in particular Lines 338-340 at the start of the Discussion section where both programs are discussed in the context of the objectives of the current study. Recommend that the authors be consistent throughout the manuscript in their reference to the Influenza Surveillance Program as to which this study is based on (ie. National, Brazilian, or just Influenza Surveillance Program are used throughout the manuscript; pick one version and capitalize all words).**

   Answer: The Brazilian Influenza Surveillance Program is part of WHO's Global Respiratory Syncytial Virus Surveillance. We chose to use the term "Brazilian Influenza Surveillance Program" with capital words, as suggested.

9. **Line 30 and Line 105: What were the remaining 44% of case caused by, all influenza?**

   Answer: In this study, only RSV and Influenza were tested. In 56% of the cases RSV was detected, the influenza virus was found in 7% of the samples and the remaining 37% cases were undetermined.

10. **Line 48: The authors should clarify in the text the source of the "Influenza and other respiratory virus epidemiological reports" as to whether these are from the Brazilian and/or National Influenza Surveillance Program.**

    Answer: We have followed previous Reviewers' recommendations to shorten the introduction, and because of that we have removed this text, as explained to the other reviewer.

11. **Line 57: The authors should explain the rationale as to why the previously observed significant association between viral load and disease severity should be more carefully studied in the Introduction. The authors later state in the Discussion that the correlation between viral load and disease severity remains controversial (Line 423). The authors are advised to further emphasize that one of the strengths of their study in finding of a lack of correlation between viral load and disease severity is the use of standardized methods for measuring viral load (see Lines 432-442)**

    Answer: To address that, we have rewritten the Introduction as follows:

"Some studies have evaluated the association between viral load and disease severity, with significant associations [6,7]. However, **most of these studies did not use standardized methods of viral load measurement**, therefore, this relationship must be more carefully evaluated."

12. **Line 60: The authors should revise this sentence to clarify that the context by which "the treatment is based" in referring to RSV since this is new paragraph.**

    Answer: We have rephrased the sentence to: "RSV treatment is based only […]".

13. **Line 72: The authors should supplement reference 15 with a reference that defines the multiple genotypes of RSV-B.**

    Answer: We have added the study by Trento et al. (2006)[1] which was already mentioned in reference #17 (now reference #14, since due to the removal of some sections to reduce the text, the corresponding references were also removed).

14. **Line 78: Reference 15 does not support the statement that understanding RSV genetic diversity will help designing antiviral drugs, diagnostic assays, and vaccines. The authors should revise.**

    Answer: It is possible to find in reference 15 (now reordered to reference 13) two excerpts that support this statement: "RSV diversity is an important factor that allows for reinfections to occur throughout life and also has implications for design of diagnostic assays, antiviral therapies, and preventive strategies (passive immunization and vaccines)". (*in the introduction*).

    "Genotype classification and assignment is of importance in order to understand the evolution, epidemiology, and clinical presentation of this virus, and has implications regarding the development of vaccines and other preventive interventions." (*in the discussion*).

15. **Fig 1: The y-axis and X-axis should be labeled within the figure.**

    Answer: Figure 1 has been edited, including caption for the two Y axes and the X axis. Caption is displayed in a text box.

16. **Line 126-127: Location of INCAPER should be provided.**

---

[1] Trento A, Viegas M, Galiano M, Videla C, Carballal G, Mistchenko AS, et al. Natural History of Human Respiratory Syncytial Virus Inferred from Phylogenetic Analysis of the Attachment (G) Glycoprotein with a 60-Nucleotide Duplication. J Virol. 2006;80: 975–984. doi:10.1128/JVI.80.2.975-984.2006

Answer: We have included the city, state and country of INCAPER. Please, check the line 145.

17. **Line 143: The authors should define in Supplemental Table 1 or elsewhere in the main text what RSV gene the primers and probes used to subtype RSV-A and RSV-B were directed against.**

    Answer: In the methodology, we include the requested information as follows: "RSV positive samples (i.e. those with cycle threshold [CT] ≤ 40) were subtyped using specific primers and probes **to N gene** of RSV-A and RSV-B." Please, check the line 105.

18. **Line 161: The authors should clarify what they mean by "partial amplification" and by RSV positive samples with Ct values between 30-40 were not subjected or attempted for sequencing.**

    Answer: Partial amplification in this case refers to the fact only part of the gene was amplified. We have included the approximate sequenced G gene fragment size, as follows:
    "The partial gene G amplification (about 730 bp) was performed at LVRS/IOC/FIOCRUZ"
    We have also included the following sentence in bold: "a) cycle threshold (ct) value less than 30, **due to the difficulty in sequencing samples with higher ct than this**;"

19. **Line 179-180: The authors should provide a reference to the source of their reference sequences.**

    Answer: The requested data is already available in supplementary tables 2 and 3. All reference sequences were taken from NCBI Genbank. These supplementary tables contain access numbers, genotypes and collection locations of each sequence.

20. **Line 37, 74, 194, 294, 334, 421, 444, 466: The authors should correct their documentation of the RSV B genotype from BA to BA1 per the accession number provided and documented.**

    Answer: The classification into the BA cluster is controversial. We prefer classify as BA. More studies are needed to standardize the RSV nomenclature of genotypes into BA and ON1.
