# Supplementary material for: Seasonality, molecular epidemiology, and virulence of Respiratory Syncytial Virus (RSV): A perspective into the Brazilian Influenza Surveillance Program
Source: PLoS One. 2021 May 18;16(5):e0251361. doi: 10.1371/journal.pone.0251361 (PMC8130917; doi:10.1371/journal.pone.0251361)
Supplement: Attachment — Submitted filename: Response to Reviewers.docx [file pone.0251361.s015.docx]

**Response to Reviewers**

Dear editors and reviewers, thank you for your attention in improving this study. Here are the answers for each observation.

Sincerely,

Lucas Alves Vianna

**Editor comments:**

**Comment 1**: Please ensure that your manuscript meets PLOS ONE's style requirements, including those for file naming. The PLOS ONE style templates can be found at:

https://journals.plos.org/plosone/s/file?id=wjVg/PLOSOne_formatting_sample_main_body.pdf and

https://journals.plos.org/plosone/s/file?id=ba62/PLOSOne_formatting_sample_title_authors_affiliations.pdf
 **Answer:** After a careful review, we have modified the formatting of the headings and legends of the supplementary figures and tables to meet PLOS ONE's style requirements. We also corrected some tables that presented values highlighted in red, contrary to the rules of PLOS ONE. Finally, we increased the font size of the Materials and methods, Results and Discussion subheadings to 16 pt, according to rules.

**Comment 2:** We note that you included minors (age<18) in your study. Please provide additional details regarding minor’s consent. In the ethics statement in the Methods and online submission information, please ensure that you have specified whether you obtained consent from parents or guardians. If the need for consent was waived by the ethics committee, please include this information.”

**Answer**: We agree with this observation. The sentence “The need for parents or guardians’ consent was waived by the ethics committee.” was included in “Ethics Statement” section. Please, check the lines 207-208.

**Reviewer #1 comments:**

1. **Title - Revise the title to reflect the key findings of the research.**

**Answer:** To address this comment, we have changed the title to: “Seasonality, molecular epidemiology and virulence of Respiratory Syncytial Virus (RSV): a perspective into the Brazilian Influenza Surveillance Program”. However, considering that this study addressed several aspects of RSV, the inclusion of key findings would make the title too large and we have opted to make it shorter and easier to read.

2. **Introduction - Revise the introduction and make it shorter and its current form it distracts the reader.**

**Answer:** We have changed the introduction and eliminated sentences that, although interesting, would not affect the understanding of the objectives. However, Introduction size reduction was small. It turns out that this study has addressed multiple aspects of RSV (*e.g.:* prevalence, association between severity and subtypes, viral load, seasonality and association with climatic factors and phylogenetic aspects), as we understand that these are important aspects to discuss.

3. **Methodology - Organize the Methodology - Study population, experimental methods - RT-qPCR, sequencing etc. in a brief form that gives a good understanding of the sequence of events with relevant methodology.**

**Answer:** We have changed the way methods are presented, in order to maintain the same pattern presented at the results.

Thus, methods are now as follows:

1. Population sampling, study period and location

2. RSV and Influenza detection and subtyping

3. Clinical and epidemiological data collection

4. Viral load quantification

5. Climate data collection

6. Partial amplification and sequencing of glycoprotein gene

7. RSV genotyping and gene G phylogenetic reconstruction

8. Statistical treatment.

9. Data availability

10. Ethics Statement

4. **Results - They need to be organized in the order of appearance as in the Methodology. You do not have to replicate all the information given in the Tables in the texts. Redundancy also distracts the reader and I found it difficult to organize the results to understand the authors' way of cohesion.**

**Answer**: We think this comment will make the manuscript easier to read. As recommended, we have reorganized the objectives in the same pattern presented in the results, and eliminated redundant information.

5. **Discussion - Again follow the order of your results in Discussion.**

**Answer:** We have organized the discussion in the same order presented in Methods and Results and created subsections in the discussion, in order to improve reading.

6. **Overall - You must revise this manuscript shortening certain sections and organizing the manuscript from I to D. Otherwise the results produced cannot be understood by the authors.**

**Answer:** To address this recommendation, we have reduced the text as much as possible, without interfering with data presentation quality and consistency. We have reduced redundancies in the results (data being presented in the text and table) and removed some excerpts throughout the manuscript that we consider less relevant.

7. **Language must be clear, correct, and unambiguous. At its current form it is difficult to follow the authors. Please also look into typographical or grammatical errors when your revise the manuscript. You may ask a native speaker to read the manuscript after fixing all the issues indicated.**

**Answer:** we have asked a native speaker to thoroughly review the manuscript.

8. **Please follow the PLOS ONE formatting guidelines well before you submit after revision.**

**Answer:** We have done so.

**Reviewer #2 comments:**

1. **The authors present generalized conclusions that are not specific to the Brazilian aspect or timeframe on which this study is based. Key results to support the identified objectives are not highlighted in the abstract or the conclusion (e.g. the influence of climate factors on RSV seasonality and the role of genetic diversity of RSV on disease severity). Clinical severity scores referenced in the abstract and results to support interpretations of the role of viral load and genetic diversity of RSV on disease severity, should be presented in the main tables/figures of the manuscript as opposed to supplemental. The authors should revise these areas and sharpen the focus of their Discussion through reduction to improve readability and presentation of key messages of RSV surveillance in Brazil between 2016-2018 relative to previous observations in Brazil or other parts of the world during the similar timeframe.**

**Answer**: We appreciate the observations. However, some results of this study are not related to a specific location or timeframe. The correlation analyzes between viral load, genetic differences and severity are examples. These results possibly transcends the time and place of the study and, therefore, are not specific to the Brazilian aspect or timeframe.

As recommended, we have included the key results in both abstract and conclusion. We also transformed supplementary table 4 into Table 3. Previous table 3, which presented data on viral load, is now part of Table 4.

Given the different approaches taken in the study, we chose to divide the discussion into topics, in the hope of improving the quality of reading and regarding the discussion length, we removed some less important passages in order to improve the readability and presentation of key messages of the study.

2. **The current title (and abstract) fail to address the presented timeframe of RSV surveillance or what aspects of “landscape” or “perspectives” the authors are referring to relative to their objectives and results. The authors should consider revision.**

**Answer**: as changing the Title was also a recommendation of Reviewer 1, and to clarify which aspects of the “landscape” the study focused on, we have changed the title to: “Seasonality, molecular epidemiology and virulence of Respiratory Syncytial Virus (RSV): a perspective into the Brazilian Influenza Surveillance Program”. We hope this new title is suitable for both Reviewers.

3. **Figure 2 and Figure 3 are out of focus and uninterruptable for review. The authors should revise.**

**Answer:** Figures 2 and 3 were redone to improve quality and readability. We decided to change the size and save the file as .EPS.

4. **Line 90-92, 130-133, and 270-276. The authors statement of “seasonal oscillation” (Line 90-92) is not supported by their main observations (Line 270-276) from Fig. 1, and in turn, their statement regarding “creating difficulties for determination of the most appropriate period to start prophylaxis” is not substantiated and is in contrast to their later statement of “recommends the administration of palivizumab from February to July” (Line 359). The authors state (Line 130-133) that “seasonality onset and end were defined as the first and last of 2 consecutive weeks, respectively, when the number of RSV cases exceeded 10% of the number detected during the RSV peak week” and reference Obando-Pacheco et al 2018 [21]. However, Obando-Pacheco et al 2018 states that “the onset of RSV season was defined as the first 2 consecutive weeks when >10% of the total tested samples for respiratory pathogens were positive for RSV. The end of the RSV season was defined similarly as when the proportion of positive RSV tests fell below 10% for 2 consecutive weeks.”. Given the impact of molecular testing on determining RSV seasonality, the authors should revise their analysis and adopt a more accepted threshold for seasonality assessment based on %RSV positive cases as opposed to the number of RSV cases to support a potential interpretation of “seasonal oscillation” (see also Midgley et al. 2017 JID 216(3):345-355).**

**Answer**: We agree with the Reviewer, and, in fact, there was a misinterpretation of season beginning and end definition by Obando-Pacheco et al. (2018). Therefore, we reviewed the data and corrected the analysis. However, there were no changes in season onset in any year, but there were small changes in season end, as described below:

1. End in 2016: from EW 33 to EW 32.

2. End in 2017: from EW 30 to EW 31.

3. End in 2018: from EW 26 to EW 27.

Although the reviewer understood that the data do not support the claim that there was a fluctuation in season period during the study, we would like to point out that in 2016 and 2017 the RSV seasonal period started at epidemiological week (EW) 12 and ended at EW 32 and 31, respectively. In contrast, in 2018 the season was anticipated to EW 3, which is 9 weeks before the start in previous years. Season end was also anticipated to EW 27. There are the reasons why we understand there was an oscillation in the seasonal period during our study. As Palivizumab is administered in five consecutive monthly doses and considering that the first dose should be administered one month before season start, this oscillation may have an impact in the administration of prophylactic drugs.

In order to make this point clearer, we have restructured the discussion paragraphs.

5. **Table 1: The authors should revise this Table to provide both numerators and denominators to allow for readability and logical follow with the main text. This will also allow the reader to appropriately follow the statistical assessment employed of relative proportions. In addition, Influenza prevalence is noted in the main text, but not in the corresponding Table 1. The authors should to revise the Table to include all relevant data for the reader.**

**Answer**: we have revised Table 1, making the requested changes.

6. **Line 225-232 and Table 2: The authors should rephrase their statement regarding “clinical features of patients affect by RSV” to better reflect clinical characteristics of patients with SARI, since clinical data are presented for the total 632 patients and the 327 patients with RSV (180 RSV-A and 147 RSV-B). The numbers and percentages in the main text reflect the total population (N=632) and not the population of patients with RSV disease (N=327). The authors should further revise this Table to provide both numerators and denominators to allow for readability and logical follow with the main text. This will also allow the reader to appropriately follow the statistical assessment employed of relative proportions and to distinguish between RSV and everything else. Finally, viral load data in Table 2 is out of place without a (%) and should be included in Table 3 where viral load values are presented.**

**Answer**: Table 2 and the associated text present clinical data only of RSV infected patients. This table had an error in the "Sample number" field, which contained the total number of samples studied (632), however, the analyzes were performed only with the 352 RSV positive children. The error has been corrected. We took the opportunity to correct some fields containing three decimal places, standardizing the values to two decimal places. We have also relocated table 2 viral load data to table 3, as suggested. Finally, we included denominators to facilitate data interpretation.

**Minor Comments for Author (Required)**

7. **Line 17 and 40. The authors are repetitive in their statements in the Background and Conclusion sections of their Abstract regarding “understanding seasonality, genetic features…may support antiviral and vaccine development. The authors should revise the abstract and clarify how the results of this study specifically support antiviral and vaccine development.**

**Answer**: We have eliminated the redundant part from the "Background" and briefly discussed how seasonal period, virulence and genetic diversity can assist in the development and application of vaccines and antiviral drugs.

8. **Lines 21, 38, 81, 83, 88, 339-440. Is the Brazilian Influenza Surveillance Program part of WHO’s Global Respiratory Syncytial Virus Surveillance Pilot and/or the Global Influenza Surveillance and Response System (GISRS)? The authors should consider revising for clarity; in particular Lines 338-340 at the start of the Discussion section where both programs are discussed in the context of the objectives of the current study. Recommend that the authors be consistent throughout the manuscript in their reference to the Influenza Surveillance Program as to which this study is based on (ie. National, Brazilian, or just Influenza Surveillance Program are used throughout the manuscript; pick one version and capitalize all words).**

**Answer**: The Brazilian Influenza Surveillance Program is part of WHO’s Global Respiratory Syncytial Virus Surveillance. We chose to use the term “Brazilian Influenza Surveillance Program” with capital words, as suggested.

9. **Line 30 and Line 105: What were the remaining 44% of case caused by, all influenza?**

**Answer**: In this study, only RSV and Influenza were tested. In 56% of the cases RSV was detected, the influenza virus was found in 7% of the samples and the remaining 37% cases were undetermined.

10. **Line 48: The authors should clarify in the text the source of the “Influenza and other respiratory virus epidemiological reports” as to whether these are from the Brazilian and/or National Influenza Surveillance Program.**

**Answer**: We have followed previous Reviewers’ recommendations to shorten the introduction, and because of that we have removed this text, as explained to the other reviewer.

11. **Line 57: The authors should explain the rationale as to why the previously observed significant association between viral load and disease severity should be more carefully studied in the Introduction. The authors later state in the Discussion that the correlation between viral load and disease severity remains controversial (Line 423). The authors are advised to further emphasize that one of the strengths of their study in finding of a lack of correlation between viral load and disease severity is the use of standardized methods for measuring viral load (see Lines 432-442)**

**Answer**: To address that, we have rewritten the Introduction as follows:

“Some studies have evaluated the association between viral load and disease severity, with significant associations [6,7]. However, **most of these studies did not use standardized methods of viral load measurement**, therefore, this relationship must be more carefully evaluated.”

12. **Line 60: The authors should revise this sentence to clarify that the context by which “the treatment is based” in referring to RSV since this is new paragraph.**

**Answer**: We have rephrased the sentence to: “RSV treatment is based only […]”.

13. **Line 72: The authors should supplement reference 15 with a reference that defines the multiple genotypes of RSV-B.**

**Answer**: We have added the study by Trento et al. (2006)^[1]^ which was already mentioned in reference #17 (now reference #14, since due to the removal of some sections to reduce the text, the corresponding references were also removed).

14. **Line 78: Reference 15 does not support the statement that understanding RSV genetic diversity will help designing antiviral drugs, diagnostic assays, and vaccines. The authors should revise.**

**Answer**: It is possible to find in reference 15 (now reordered to reference 13) two excerpts that support this statement: “RSV diversity is an important factor that allows for reinfections to occur throughout life and also has implications for design of diagnostic assays, antiviral therapies, and preventive strategies (passive immunization and vaccines)”. (*in the introduction*).

“Genotype classification and assignment is of importance in order to understand the evolution, epidemiology, and clinical presentation of this virus, and has implications regarding the development of vaccines and other preventive interventions.” (*in the discussion*).

15. **Fig 1: The y-axis and X-axis should be labeled within the figure.**

**Answer**: Figure 1 has been edited, including caption for the two Y axes and the X axis. Caption is displayed in a text box.

16. **Line 126-127: Location of INCAPER should be provided.**

**Answer**: We have included the city, state and country of INCAPER. Please, check the line 145.

17. **Line 143: The authors should define in Supplemental Table 1 or elsewhere in the main text what RSV gene the primers and probes used to subtype RSV-A and RSV-B were directed against.**

**Answer**: In the methodology, we include the requested information as follows: “RSV positive samples (i.e. those with cycle threshold [CT] ≤ 40) were subtyped using specific primers and probes **to N gene** of RSV-A and RSV-B.” Please, check the line 105.

18. **Line 161: The authors should clarify what they mean by “partial amplification” and by RSV positive samples with Ct values between 30-40 were not subjected or attempted for sequencing.**

**Answer**: Partial amplification in this case refers to the fact only part of the gene was amplified. We have included the approximate sequenced G gene fragment size, as follows:

“The partial gene G amplification (about 730 bp) was performed at LVRS/IOC/FIOCRUZ”

We have also included the following sentence in bold: “a) cycle threshold (ct) value less than 30, **due to the difficulty in sequencing samples with higher ct than this**;”

19. **Line 179-180: The authors should provide a reference to the source of their reference sequences.**

**Answer**: The requested data is already available in supplementary tables 2 and 3. All reference sequences were taken from NCBI Genbank. These supplementary tables contain access numbers, genotypes and collection locations of each sequence.

20. **Line 37, 74, 194, 294, 334, 421, 444, 466: The authors should correct their documentation of the RSV B genotype from BA to BA1 per the accession number provided and documented.**

**Answer:** The classification into the BA cluster is controversial. We prefer classify as BA. More studies are needed to standardize the RSV nomenclature of genotypes into BA and ON1.

1. Trento A, Viegas M, Galiano M, Videla C, Carballal G, Mistchenko AS, et al. Natural History of Human Respiratory Syncytial Virus Inferred from Phylogenetic Analysis of the Attachment (G) Glycoprotein with a 60-Nucleotide Duplication. J Virol. 2006;80: 975–984. doi:10.1128/JVI.80.2.975-984.2006
