# Supplementary material for: Seasonality, molecular epidemiology, and virulence of Respiratory Syncytial Virus (RSV): A perspective into the Brazilian Influenza Surveillance Program
Source: PLoS One. 2021 May 18;16(5):e0251361. doi: 10.1371/journal.pone.0251361 (PMC8130917; doi:10.1371/journal.pone.0251361)
Supplement: Attachment — Submitted filename: The authors have made a solid effort at addressing the previous reviewers.docx [file pone.0251361.s016.docx]

The authors have made a solid effort at addressing the previous reviewers’ concerns only to be confronted with a new reviewer who hereby adds his own concerns. I have limited these to the areas less covered by previous reviewer and I acknowledge that the appear to have addressed most of the previous reviewer’s concerns. Still, there are still some problems here, most of which are easy enough to fix, that undermine the hard work that these researchers have done. The science (like most decent science) seems workmanly but competent and the findings are important enough to be worthy of publication even if they lack the glamour sought in other outlets.

**Sample selection**

How were the municipalities selected? Was sampling representative? And if so was a weighting scheme used that would allow improved generalization of the results? Given the international audience of PLoS, I would favor including a map with distance marker, topography and major roads be provided perhaps this could be done as a link to a google map page would be helpful and avoid the impression of being a gimmick to Brazilian readers while helping international ones.

Median length of stay was high at 8 days suggesting this was a sicker than usual group of infants with RSV. The potential for this later result should be signaled in the sampling scheme.

**Genetic analysis**

I defer this to the prior Reviewer #2 who knows much more about this than I do.

**Clinical Outcomes and study definitions**

Dichotomizing 02 saturation, especially at 95% which is not a clinically used threshold seemed odd. 90%, 92% or even 94% would be consistent with variously described clinical thresholds.

CSS score seems very crude. Was it derived and validated before this study? If not, it is simply reframing the data already gathered and would be better analyzed as discrete variables rather than as a score. It would be better to analyze the CSS itself as an ordinal variable. This would also increase the statistical power of the analysis.

Dyspnea (p=0.148) and respiratory distress (p=0.002) seem difficult to distinguish to me. Do the authors mean tachypnea? If so, what rate/age thresholds were used? Invasive versus non-invasive 02 therapies.

**Viral load and clinical outcomes**

Viral load data

“According to age, median viral load was higher in children with 4 to 6 months old (63.0 261 cop/cell, p=0.007). Regarding patient clinical conditions, we found lower viral load I 262 patients with fever (26.15 cop/cell) than those without it (111.29 cop/cell; p=0.00)”

A key point is this: were those children who had no fever treated with antipyretics? If so then maybe this is an effect of the antipyretic which has been shown to increase viral shedding in animal models of RSV and duration of shedding in rhinovirus. If they were not the implication may be that fever was reflecting an antibody response to higher viral loads. (Also, write this as p<0.01.)

(Table 4 and line 265) “Although lacking statistical support (p=0.089), a noteworthy observation is the tendency of lower viral load in patients with elevated CSS.” The viral load analysis was performed regardless of time between symptom onset and date of collection, which, in theory, could alter the interpretation. However later it appears that they did a sensitivity analysis implying Day 7 measures had essentially the same results as other time points? It was unclear to me exactly what they mean here.

The authors also found fever and viral load to be inversely related. This is important because it is counterintuitive at first blush but makes complete sense when interpreted in the context of RSV being capable of infecting via aerosols reaching the alveoli as well as droplets seeding the upper airways. If lung ultrasound data were available that would be an interesting way to address this apparent paradox. (Probably requires another paper though.)

**Race**

The authors’ use of race is baffling to me. Rather than using race which is suspect at the best of times, and per their discussion, especially in Brazil, could the authors use SES? If race is important, and their own discussion suggests otherwise, some classification other than color is needed. If it can’t be explained it should be dropped.

**Climate data**

Line 368 on Precipitation rate and relative humidity percentage have not been shown to influence the distribution of RSV cases by Spearman's correlation test (p = 0.55 and 0.11, respectively). The mean temperature, however, showed a minor and inverse correlation with RSV infections (-0.16; p = 0.05). Please use 'did not' rather 'have not been shown' if these are your findings rather than someone else’s. If they are from someone else, please include the reference.

**Tables**

**Table 1**

Unclear why there are P-values in Table 1 unless the authors are testing a hypothesis that there were important differences between seasons. If they are, then they should say this and incorporate season as a variable in the final model

The authors report (%) implying that percentages are in parentheses however it appears that it is the proportions they are reporting in parentheses.

**Table 2**

This is important. I would have placed death with the outcomes/clinical profile. In demographics it is sufficient to provide the number for one gender only. Indicating the use of a specific test as a Table foot note is needed only if not already in the methods or is less commonly used.

**Table 3**

Table 3 appears to analyze CSS as categorical rather than ordinal data. Please be consistent with format for decimals; either, or. but do not use them interchangeably. I expect PLoS favors the US/UK format of ‘.’.

It would be better to present the components of the CSS individually and the CSS itself as an ordinal variable. This would also increase the statistical power of the analysis.

**Table 4**

Same comments as for prior tables apply. Days in intensive care should be compared using a survival analysis technique. If a p value appears as ‘= 0.000’ in computer software, please report it as p <0.001.

**Figure 1**

Please add a second X -axis below the current one to indicate season or month.

I have not been able to down load the supplemental materials – I just get a pdf of the submission

**Discussion**

An explanation not raised by the authors is that maybe these children had predominantly

lower respiratory tract disease. This could arise where the bulk of the viral transmission

occurred via the airborne rather than the large droplet route and that some of the infection started

in the alveoli rather than the larger airways. This would lead to a more rapid onset and more severe disease.

See above comments for the other areas of concern. This is slightly more nuanced than the previous reviewers who argue the data shows no correlation between viral load and disease. I think the nuance is warranted.
