# Supplementary material for: Seasonality, molecular epidemiology, and virulence of Respiratory Syncytial Virus (RSV): A perspective into the Brazilian Influenza Surveillance Program
Source: PLoS One. 2021 May 18;16(5):e0251361. doi: 10.1371/journal.pone.0251361 (PMC8130917; doi:10.1371/journal.pone.0251361)
Supplement: Attachment — Submitted filename: Answers to Reviewers.docx [file pone.0251361.s018.docx]

**Response to Reviewers**

**Editor’s comments:**

**The reviewers agreed that the revised manuscript showed significant improvement, however, one reviewer still have concern regarding clinical data, which needs to be address carefully.  In addition, I have found quite a few typos and error within the manuscript that need to be corrected (see attached PDF file from editor).**

**Answer:** Dear Editor, thank you for considering our manuscript. We thoroughly checked comments in the PDF file and made all suggested corrections

**Comment 1: About the sentence: "RSV viral load was determined by RT-qPCR using a protocol adapted from Álvarez-Argüelles et al. [20], including a synthetic β-globin dsDNA as a template.”, it was asked if the β-globin dsDNA was used as an internal PCR control.**

**Answer:** β-globin dsDNA was used for sample cell quantification; using the same standard curve construction method applied for measuring RSV copies, we were able to calculate the number of cells in the sample. Applied Biosystems 7500 Real-Time PCR Software is able to use various known concentrations of β-globin (comparing these curves with the sample amplification curve) to estimate number of β-globin copies in the sample. This number can later be used to calculate the number of cells in a sample. In time, the marker used as an internal control was the human RNAse P gene.

**Comment 2: About the sentence: "** **Male gender was 220 slightly more affected by RSV (n=182; 52%) […]” it was asked whether the finding was statistically significant.**

**Answer:** Negative. The difference found in the proportion between male and female patients affected by RSV presented no statistical support. However, as other authors commonly present these data, we decided to show it as well. In addition, we compared viral load between genders.

**Comment 3: About the sentence: “S4 Table shows the difference in severity by ethnicity.”, it was asked for more description.**

**Answer:** To address the request of reviewer #3 (Comment n°9), we decided to eliminate discussions about race from the manuscript, and, therefore, the S4 table was removed.

**Comment 4: Please review your reference list to ensure that it is complete and correct. If you have cited papers that have been retracted, please include the rationale for doing so in the manuscript text, or remove these references and replace them with relevant current references. Any changes to the reference list should be mentioned in the rebuttal letter that accompanies your revised manuscript. If you need to cite a retracted article, indicate the article’s retracted status in the References list and also include a citation and full reference for the retraction notice.**

**Answer:** We use the Zotero software as an indexer of bibliographic references. In this way, all the works cited are listed automatically. Even so, we revised the manuscript to ensure that all and only the papers cited are referenced.

Once the discussion in the context of race was removed from the manuscript, the references used were also eliminated, namely:

- Reference 31 (Cardena et al., 2005);
- Reference 32 (Brasil. Síntese de Indicadores Sociais: uma análise das condições de vida da população brasileira);
- Reference 33 (Nair et al., 2010)

We also added a reference (de-Paris et al., 2014) in order to include data on length of hospital stay in the discussion. This measure aimed to meet the recommendation of reviewer #3 to emphasize the data on duration of hospitalization found in our study.

Two other references (Thomas, 2013 [ref. 30] and Wonderlich et al, 2017 [ref. 31]) were added in order to support the discussion suggested by the reviewer in his comment No. 17.

We did not use papers that have been retracted.

**Reviewer #3 comments:**

**Topic n°1: Sample selection**

**Comment n°1: How were the municipalities selected? Was sampling representative? And if so was a weighting scheme used that would allow improved generalization of the results? Given the international audience of PLoS, I would favor including a map with distance marker, topography and major roads be provided perhaps this could be done as a link to a google map page would be helpful and avoid the impression of being a gimmick to Brazilian readers while helping international ones.**

**Answer:** Most of our study was carried out in the Molecular Biology II sector of the Central Laboratory of Espirito Santo (LACEN/ES). This government agency is part of the national laboratory surveillance network for Influenza and other respiratory viruses and, for this reason, has the duty to investigate all cases that fit the SARI concept adopted in Brazil. Thus, any sample collected in this context, even if it comes from private institutions, must be sent to LACEN for diagnosis of Influenza, Respiratory Syncytial Virus and, most recently, of SARS CoV 2. Therefore, no sampling or weighting scheme was used, for the simple fact that virtually all samples of children up to 3 years old with SARI in Espirito Santo were present in our study. Consequently, the sampled municipalities were all those with suspected cases (61 municipalities). Of these, 46 presented at least one child with a confirmed case of RSV infection.

We have built a map of the state of Espirito Santo (**S1 Fig.**), with the main highways (federal highways), distance marker and topography, as requested. We plotted the number of confirmed RSV cases in each municipality throughout the study period. We are grateful for the idea, which will help the international community better understand the geographic space of the study.

**Comment n°2: Median length of stay was high at 8 days suggesting this was a sicker than usual group of infants with RSV. The potential for this later result should be signaled in the sampling scheme.**

**Answer:** To address this comment, we have included the following text:

*"Although the median length of hospitalization found here is similar to some other studies [28,29], notably most studies report a shorter duration [1,4,26]. One hypothesis that could explain this finding is the fact that all children included in our study were diagnosed with SARI, which makes our study group a cohort with severe RSV infection. Another hypothesis is linked to the possibility that most of the children in the study had an infection in the lower respiratory tract. Aerosol transmission increases the chances of inhaling viral particles in the lower airways, while larger droplets are retained in the upper airways [30]. Naturally, aerosol infections tend to trigger a more severe course of infection [31].*”

We would like to point that there was no sampling scheme, as all the samples in the Espirito Santo State are sent to us for analysis.

**Topic n°2: Clinical Outcomes and study definitions**

**Comment n°3: Dichotomizing 02 saturation, especially at 95% which is not a clinically used threshold seemed odd. 90%, 92% or even 94% would be consistent with variously described clinical thresholds.**

**Answer**: This is a retrospective study in which all clinical data were taken directly from the SARI epidemiological records, used by the Brazilian health system. In these forms, there is a field with the following question: “Oxygen saturation ≤ 95%?”. There is no field in the records for filling with the measured saturation *per se*, but only if it was above, equal or below 95%. It is, therefore, qualitative data. The reviewer very well pointed out that the dichotomization of O_2_ saturation is not commonly clinically used; however, unfortunately, this was the only possible way of analysis, given the data available. However, it is necessary to say that saturation ≤95% is used as an element in the definition of SARI in Brazil and, although it is not the most common form found in studies, it is used as an indicator of severity in respiratory diseases.

**Comment n°4: CSS score seems very crude. Was it derived and validated before this study? If not, it is simply reframing the data already gathered and would be better analyzed as discrete variables rather than as a score. It would be better to analyze the CSS itself as an ordinal variable. This would also increase the statistical power of the analysis.**

**Answer**: The CSS used in our study was adapted from Martinello and colleagues, 2002 (see the comparison in the table below). The adaptations were made due to subtle differences in the way data was obtained. For example, the way of measuring oxygen saturation in our study, as explained in response to comment No. 3, was obtained in a binary way (yes or no) in relation to a saturation lower than 95%. In contrast, Martinello et al., 2002 used the threshold of 87%. CSS was a way that we found to combine the data most associated with disease severity in a single parameter. It is the use of the basic principle of "reduction of dimensionality" of the problem. In our view, CSS was treated as an ordinal categorical variable, and not as a discrete variable. In fact, CSS cannot be used as a discrete value since "score # 2" is not twice as severe as score # 1, for example.

|  | **Martinello et al., 2002** | **This study** |
| --- | --- | --- |
| **Assessed factors** | **Number of points** | |
| Mechanically ventilator support | 2 | 2 |
| Use of supplemental oxygen | 1 | 1 |
| Hospital admission | 1 | - * |
| Hospitalization ≥ 5 days | 1 | 1 |
| Oxygen saturation ≤ 87% | 1 | 1 |
| ICU admission | - | 1 |
| **Range** | 0-6 points | 0-5 points** |

* Since virtually all patients in our study were diagnosed with SARI, they were all hospitalized. In this way, everyone would receive 1 point on the scale used by Martinello et al., 2002. As only the most serious cases required admission to ICU, we modified the score for this situation.

** In our study, a patient who was initially treated with oxygen supplementation, however, due to the worsening of the case, required mechanical ventilation received 2 points and not 3 for the fact that he was, technically, in both categories.

**Comment n°5: Dyspnea (p=0.148) and respiratory distress (p=0.002) seem difficult to distinguish to me. Do the authors mean tachypnea? If so, what rate/age thresholds were used? Invasive versus non-invasive 02 therapies.**

**Answer:** The SARI notification form used by the Brazilian health system includes both parameters: dyspnea and respiratory distress, that should be informed by the doctor or nurse during the patient's care, and yes, these are synonyms. Dyspnea is a more technical term that, according to the American Thoracic Society (ATS), is defined as a subjective experience of respiratory distress. But as people can generally only describe what they can understand, a more lay term is also present in the notification. Our group discussed the possibility of eliminating one term, but opted to keep both terms in the analysis, since this is how data was originally collected and analyzed.

**Topic n°3: Viral load and clinical outcomes**

**Comment n°6: “According to age, median viral load was higher in children with 4 to 6 months old (63.0 261 cop/cell, p=0.007). Regarding patient clinical conditions, we found lower viral load I 262 patients with fever (26.15 cop/cell) than those without it (111.29 cop/cell; p=0.00)”. A key point is this: were those children who had no fever treated with antipyretics? If so then maybe this is an effect of the antipyretic which has been shown to increase viral shedding in animal models of RSV and duration of shedding in rhinovirus. If they were not the implication may be that fever was reflecting an antibody response to higher viral loads. (Also, write this as p<0.01.)**

**Answer:** All clinical information was obtained from SARI notification forms. In these forms, in most cases, the drugs used in the treatment are not listed. That is why it is impossible to retrieve information about which patients used antipyretics or not. Thus, although it was a very interesting recommendation by the reviewer, unfortunately it is not possible to carry out this type of analysis.

**Comment n°7: (Table 4 and line 265) “Although lacking statistical support (p=0.089), a noteworthy observation is the tendency of lower viral load in patients with elevated CSS.” The viral load analysis was performed regardless of time between symptom onset and date of collection, which, in theory, could alter the interpretation. However later it appears that they did a sensitivity analysis implying Day 7 measures had essentially the same results as other time points? It was unclear to me exactly what they mean here.**

**Answer:** The CSS was calculated based on parameters generally associated with the severity of the infection, such as the need for intensive therapies, the use of mechanical ventilation, hospitalization, etc. When comparing the median viral load between the different clinical severity scores, we noticed a tendency for the lowest scores (less severe disease) to have a higher viral load. This observation could indicate an inversely proportional relationship between viral titers and the severity of the disease. Although it seems to be an unexpected relationship, other authors found very similar results (Haynes et al., 2013; Piedra et al., 2017). Knowing that the tendency of viral load is to decrease as the disease progresses, a possible common doubt to the reader would be whether these findings could not be associated with a bias caused by different sample collection dates. Therefore, we performed a serial analysis in which the results were very similar to the result using the sampling indistinctly from the date of collection. However, in our study there was no statistical significance for the relationship found, which limits us to go further in the discussion.

**Comment n°8: The authors also found fever and viral load to be inversely related. This is important because it is counterintuitive at first blush but makes complete sense when interpreted in the context of RSV being capable of infecting via aerosols reaching the alveoli as well as droplets seeding the upper airways. If lung ultrasound data were available that would be an interesting way to address this apparent paradox. (Probably requires another paper though.)**

**Answer:** Once again, we would like to thank the reviewer's brilliant insights. However, unfortunately, we do not have lung ultrasound data on patients. This analysis, nevertheless, can be the focus of a new prospective study for more robust investigations in this and other aspects.

**Topic n°4: Race**

**Comment n°9: The authors’ use of race is baffling to me. Rather than using race which is suspect at the best of times, and per their discussion, especially in Brazil, could the authors use SES? If race is important, and their own discussion suggests otherwise, some classification other than color is needed. If it can’t be explained it should be dropped.**

**Answer:** In fact, the inclusion of this discussion in the study raised doubts even among the authors. The idea would be to bring more data about a topic that has already been addressed in other studies (doi:10.1542/peds.2004-0059) and not to cause discomfort to readers. This comment was important to reinforce that the comparison between races, although in the best intention of evaluating possible differences between ancestry and response to infections, is inappropriate. In addition, as the reviewer rightly stated, our data do not suggest that there is a direct relationship between the severity of the infection and ethnicities. So, we appreciate the feedback and decided to remove this topic from the study.

**Topic n°5: Climate data**

**Comment n°10: Line 368 on Precipitation rate and relative humidity percentage have not been shown to influence the distribution of RSV cases by Spearman's correlation test (p = 0.55 and 0.11, respectively). The mean temperature, however, showed a minor and inverse correlation with RSV infections (-0.16; p = 0.05). Please use 'did not' rather 'have not been shown' if these are your findings rather than someone else’s. If they are from someone else, please include the reference.**

**Answer:** Those are our findings and, therefore, we made the correction as advised.

**Topic n°6: Table 1**

**Comment n°11: Unclear why there are P-values in Table 1 unless the authors are testing a hypothesis that there were important differences between seasons. If they are, then they should say this and incorporate season as a variable in the final model**

**Answer:** The reviewer's comment is pertinent. Yes, the p-value in table 1 resulted from a comparison of the scenarios between the seasons, which shows that some of the difference observed is not accidental. However, due to the length of the manuscript and following the suggestion of the other reviewers to decrease the size of the discussion and sharpen the focus in some areas, we chose not to discuss these results. Taking into account that data without discussion adds little to scientific knowledge, we chose to remove this statistical data from the table, which will make it cleaner and easier to interpret. We are grateful for the observation.

**Comment n°12: (Comment about Table 1) The authors report (%) implying that percentages are in parentheses however it appears that it is the proportions they are reporting in parentheses.**

**Answer:** We would like to thank the reviewer for the observation. Certainly, the header of the tables indicated that the relationship would be presented in percentage, but the data were presented in proportion. We changed the presentation to the percentage format (%) as shown in the table.

**Topic n°7: Table 2**

**Comment n°13: This is important. I would have placed death with the outcomes/clinical profile. In demographics it is sufficient to provide the number for one gender only. Indicating the use of a specific test as a Table foot note is needed only if not already in the methods or is less commonly used.**

**Answer:** As advised, we moved the RSV death data to the "Clinical profile" category, kept the gender data for a single gender and removed the table foot note containing the statistical tests performed, since they were frequently used methods.

**Topic n°7: Table 3**

**Comment n°14: Table 3 appears to analyze CSS as categorical rather than ordinal data. Please be consistent with format for decimals; either, or. but do not use them interchangeably. I expect PLoS favors the US/UK format of ‘.’. It would be better to present the components of the CSS individually and the CSS itself as an ordinal variable. This would also increase the statistical power of the analysis.**

**Answer**: As reported in comment n°4, CSS was analyzed as an ordinal variable. In addition, the components used in the construction of the CSS were treated individually in the comparison between the subtypes RSV-A and RSV-B (Table 2). Finally, we inform that we have corrected the field in which there was a "," instead of a “.” in table 3. All other numbers followed the US / UK format.

**Topic n°8: Table 4**

**Comment n°15: Same comments as for prior tables apply. Days in intensive care should be compared using a survival analysis technique. If a p value appears as ‘= 0.000’ in computer software, please report it as p <0.001.**

**Answer:** As requested, we performed the survival curve of patients who required admission to the ICU and, for this, we used the non-parametric Kaplan-Meier test. However, considering that of the patients with data on ICU entry and exit, only 5 died and 134 had “recovery” as an outcome, we used the cure outcome and not death as usual as "failure". That is, the test revealed that the average time until the recovery of children admitted to ICU was 8 days, from the first day of intensive care. From this, we used the Cox regression (or proportional hazards regression) in order to assess whether the viral load would have a statistically significant effect on the duration of ICU stay. Since the p-value was greater than 0.05, we can infer that there is no such effect. We emphasize that we use Schoenfeld residuals and that the proportional hazards assumption were met. We also modified presentation of the p-values as suggested by the reviewer.

**Topic n°8: Figure 1**

**Comment n°16: Please add a second X -axis below the current one to indicate season or month. I have not been able to download the supplemental materials – I just get a pdf of the submission.**

**Answer:** Below the X-axis we inserted three dividing lines that represent each of the years (seasons) of the study.

**Topic n°9: Discussion**

**Comment n°17: An explanation not raised by the authors is that maybe these children had predominantly lower respiratory tract disease. This could arise where the bulk of the viral transmission occurred via the airborne rather than the large droplet route and that some of the infection started in the alveoli rather than the larger airways. This would lead to a more rapid onset and more severe disease. See above comments for the other areas of concern. This is slightly more nuanced than the previous reviewers who argue the data shows no correlation between viral load and disease. I think the nuance is warranted.**

**Answer:** Initially, we had doubts about which point in the discussion of the results the reviewer wanted to address that question. However, we believe that it fits properly in the discussion regarding the prolonged hospital stay observed in our study. Thus, we included the following text (see answer to comment n°2):

*“Although the median length of hospitalization found here is similar to some other studies [28,29], notably most studies report a shorter duration [1,4,26]. One hypothesis that could explain this finding is the fact that all children included in our study were diagnosed with SARI, which makes our study group a cohort with severe RSV infection. Another hypothesis is linked to the possibility that most of the children in the study had an infection in the lower respiratory tract. Aerosol transmission increases the chances of inhaling viral particles in the lower airways, while larger droplets are retained in the upper airways [30]. Naturally, aerosol infections tend to trigger a more severe course of infection [31].”*

**Topic n°9: Clinical dataset**

**Comment n°18: The clinical dataset does not appear to have been uploaded in the way the genetic data has. Some of the statistical choices are suboptimal but overall this is decent if unglamorous science that fits the PLoS model.**

**Answer:** In order to meet the reviewer's requirements, we decided to upload the table with general data (including clinical data) as a supplementary table (**S4 Table**) in the materials and methods section. In this way, clinical data are also accessible as suggested by the reviewer. Regarding the statistical analysis, we performed the survival analysis recommended in comment n°15.
