# Supplementary material for: Seasonality, molecular epidemiology, and virulence of Respiratory Syncytial Virus (RSV): A perspective into the Brazilian Influenza Surveillance Program
Source: PLoS One. 2021 May 18;16(5):e0251361. doi: 10.1371/journal.pone.0251361 (PMC8130917; doi:10.1371/journal.pone.0251361)
Supplement: Attachment — Submitted filename: Response to Reviewers.docx [file pone.0251361.s019.docx]

**Response to Reviewers**

We thank the editor for the critical assessment of our manuscript. In the following we address the concerns point by point.

**Editor’s comments:**

**While the revised manuscript is scientifically sound, I have a few comments that need to be addressed.**

**Comment n°1:** Line 61-62, only one study is cited, so please change "..., other studies..." to "..., other study..."

**Reply:** We have done so.

**Comment n°2:** S1 Table, please include PCR conditions.

**Reply**: As suggested, we include thermocycling conditions for each set of primers and probes used.

**Comment n°3:** Line 171, there is no need to cite reference 22, suggest delete.  Please change "...Sequencher 5.1 [22]." to "...Sequencher 5.1 (Gene Codes Corporation, Ann Arbor, MI, USA).

**Reply**: We removed the reference and included the recommended excerpt.

**Comment n°4**: Line 224, please delete the statement "Male gender was slightly more affected by RSV", since it is not statistically significant and not supported by the data from 2016 (Table 1).

**Reply**: We have done so.

**Comment n°5**: Line 232 - 235, are the number of 341, 307, 336, 342 and 252 out of the total 632 patients? I am trying to clarify whether the authors mean that out of 632 patients, 341 experienced cough and 318 (93%) are RSV+ etc.?

**Reply:** We are grateful for the editor's observation, as this would possibly be a common question for readers. Therefore, we decided to include the following excerpt in the caption of Table 2:

“Although the study included 352 patients with RSV, it is possible to observe that the denominators in the clinical profile differ from this number. This occurred because not all clinical data were recorded for all patients.”

**Comment n°6**: Line 272 - 274, not sure what the authors wish to convey, this sentence needs rephrasing for clarity.

**Reply:** We rephrased the sentence as follows**:**

*“However, a segmented analysis (0-3; 4-7 and >7 days between symptom onset and sample collection) revealed very similar results. Furthermore, of the 156 samples used to measure viral titers, only 26 (16%) were collected 7 days after symptoms onset. Therefore, we prefer to maintain full sampling for viral load analysis.”*

**Comment n°7**: Figure legends for figures 2&3 need correction since no highlighted in bold in the figures.

**Reply:** Fixed.

**Comment n°8:** Line 516 - 518, delete this sentence or move to the very beginning of the conclusion.

**Reply:** The section was deleted.

**Comment n°9:** Additionally the quality of language still needs improvement.  We suggest you thoroughly copyedit your manuscript for language usage, spelling, and grammar. If you do not know anyone who can help you do this, you may wish to consider employing a professional scientific editing service.

**Reply:** Manuscript editing was performed by other two researchers with proficiency in the English language and also by a professional reviewer with experience in scientific journals. We hope that this time the spelling, grammar, and language are in perfect harmony with the formal language.
